# An Organometallic [2]Catenane With Pt‒(di‐NHC)‒Pt Units: A Topology‐Driven Strategy for Enhanced Phosphorescence in Discrete Aggregates

**DOI:** 10.1002/anie.1981919

**Published:** 2026-03-23

**Authors:** Ye Lu, Xi‐Dong Wu, Zi‐Yan Min, Lu An, Shi‐Ping Yang, F. Ekkehardt Hahn

**Affiliations:** ^1^ The Education Ministry Key Lab of Resource Chemistry Shanghai Frontiers Science Center of Biomimetic Catalysis Shanghai Normal University Shanghai P.R. China; ^2^ Institut Für Anorganische und Analytische Chemie Universität Münster Münster Germany

**Keywords:** aggregation‐induced phosphorescence enhancement, catenanes, N‐heterocyclic carbenes, self‐assembly, supramolecular chemistry

## Abstract

Mechanically interlocked molecules (MIMs) can be conceptualized as a distinct class of discrete and soluble aggregates, where the mechanical bond restricts selected intramolecular motions, thereby triggering emission enhancement analogous to aggregation‐induced emission (AIE). Herein, we present phosphorescent MIMs based on a benzobiscarbene bridged dinuclear Pt^II^ complex (NHC = *N*‐heterocyclic carbene ligand). Tailoring the *N*‐substituents of the benzobiscarbene ligand allowed selective access to the *syn‐*configured dinuclear complex *syn‐*[Pt_2_(**1b**)X_2_] (X = I, OTf). Self‐assembly of *syn‐*[Pt_2_(**1b**)(OTf)_2_] with bipyridyl ligands **L^2^** and **L^3^** yielded two discrete MIMs: the Borromean rings [**2‐BRs**](OTf)_12_ stabilized by π···π interactions and the [2]catenane [**3b‐IL**](OTf)_4_, generated and stabilized by solvophobic effects. While the π···π interactions in the Borromean rings lead to severe phosphorescence quenching, the solvophobically assembled [2]catenane effectively circumvents such quenching, while simultaneously restricting intramolecular rotations and vibrations. Consequently, the [2]catenane displays a drastic phosphorescence enhancement compared to the Borromean rings.

## Graphical abstract

The mechanically interlocked Borromean rings are stabilized by π···π stacking interactions while the [2]catenane is stabilized by solvophobic effects. While the π···π interactions in the Borromean rings lead to severe luminescence quenching, the solvophobically assembled [2]catenane effectively circumvents these interactions, simultaneously restricting intramolecular rotations and leading to a drastic phosphorescence enhancement.

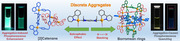

## 1 Introduction

Coordination‐driven self‐assembly has emerged as the preeminent strategy for the constructing of mechanically interlocked molecules (MIMs) owing to its high efficiency and synthetic variability [1, 2]. Various metal ions or metal‐containing building blocks have been utilized to construct these sophisticated architectures, including half‐sandwich Cp^*^Ir^III^ and Cp^*^Rh^III^ units (Cp^*^ = pentamethylcyclopentadienyl) [3, 4, 5, 6, 7, 8, 9], (cymene)Ru^II^ units [10, 11, 12], (ethylenediamine)Pd^II^ units or (alkyl phosphine)Pt^II^ units [13, 14, 15, 16, 17], Pd^II^ centres coordinated by banana‐shaped pyridyl ligands [18, 19, 20, 21], metal‐NHC motifs (NHCs = *N*‐heterocyclic carbene ligands) [22, 23, 24, 25, 26, 27, 28] and others [29, 30, 31, 32, 33]. However, the metal centres in these examples primarily serve as structural nodes, with their intrinsic properties largely overlooked [34]. For instance, although both Ir^III^ and Pt^II^ ions possess excellent phosphorescent properties, the presence of the half‐sandwich Cp^*^ ligand typically leads to severe luminescence quenching [35]. Similarly, while alkylphosphine ligands do not quench emissions, they fail often to efficiently sensitize the Pt^II^ centre [36]. Consequently, emission from such assemblies typically originates from the fluorescence of the organic ligands rather than the desired phosphorescence from the metal centre [37].

Distinctly different from fluorescence, which arises from the radiative decay of a singlet excited state (*S*_1_) to the ground state (*S*_0_), phosphorescence is the spin‐forbidden radiative decay from a triplet excited state (*T*_1_) back to the ground state (*S*_0_) [38]. Owing to this unique mechanism and the associated long lifetimes of the exited state, phosphorescent molecules are ideal for specialized applications, such as time‐resolved luminescence imaging and Organic Light‐Emitting Diodes (OLEDs) [39, 40, 41]. However, the extended *T*_1_ lifetime simultaneously renders the excited state highly susceptible to non‐radiative deactivation, making phosphorescence significantly more prone to quenching than fluorescence [42].

Over the past two decades, Aggregation‐Induced Emission (AIE), a phenomenon characterized by enhanced luminescence upon restriction of intramolecular motion in the aggregated solid state, has attracted significant attention [43, 44]. Rigidifying molecular scaffolds via coordination bonds to suppress intramolecular rotation represents an alternative effective strategy for achieving emission enhancement, particularly within discrete and soluble molecules [45, 46, 47, 48]. However, while the vast majority of reported systems based on AIE or confinement‐induced emission are limited to fluorescence, strategies to achieve Aggregation‐Induced Phosphorescence Enhancement (AIPE) remain rare [49]. MIMs can be viewed as a unique form of “discrete aggregates” wherein, the interlocking topology inherently restricts intramolecular rotation and vibration [50, 51]. Consequently, generating metallosupramolecular MIM structures and activating the phosphorescent properties of the metal centres in these structures may lead to discrete AIPE systems. Unlike traditional AIE systems that rely on nanoparticle formation or precipitation, MIMs offer a distinct advantage as they can achieve emission enhancement while maintaining a discrete and soluble state of the metal‐ligand assemblies involved [52].

In order to achieve AIPE with MIMs, two key issues need to be addressed. First, π···π stacking in the MIM assemblies must be prevented as such interactions are known to significantly weaken or even quench luminescence [53]. However, π···π stacking is a common feature in MIMs and often even the dominant driving force for their formation. Second, ligands suitable for sensitizing the metal centre for phosphorescent emission should be employed. In the best scenario, these ligands simultaneously serve as building blocks for the mechanically interlocked structures.

Bis‐*N*‐heterocyclic carbene (bis‐NHC) ligands are ideal candidates to satisfy both of these requirements [54, 55, 56]. As demonstrated previously, binuclear complexes based on bis‐NHC ligands can be utilized to construct mechanically interlocked structures driven solely by solvophobic effects [23, 24]. This approach effectively circumvents the reliance on π···π stacking for the formation of MIMs, thereby avoiding its detrimental impact on phosphorescence [57]. Furthermore, NHC ligands in combination with Pt^II^ or Ir^III^ metal centres are highly advantageous for enhancing metal phosphorescence, as the strong σ‐donating ability of NHC ligands significantly enhance the ligand field strengths around the metal centre. This effect destabilizes the non‐radiative d‐d states, thereby effectively supressing thermal quenching and boosting phosphorescence efficiency [58].

**SCHEME 1:**
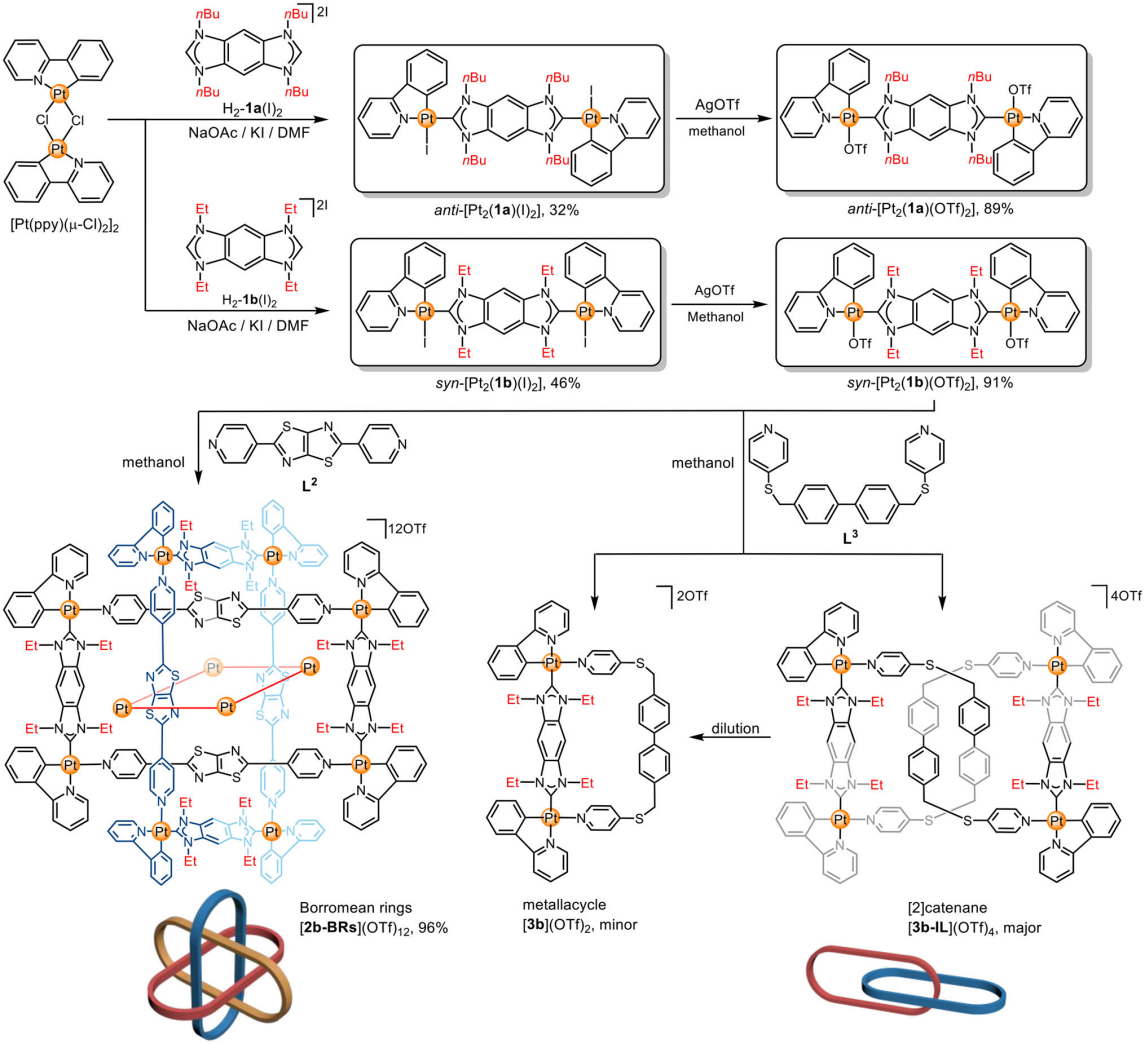
Synthesis of *anti*‐[Pt_2_(**1a**)](I)_2_ *anti*‐[Pt_2_(**1a**)](OTf)_2_, *syn*‐[Pt_2_(**1b**)](I)_2_, *syn*‐[Pt_2_(**1b**)](OTf)_2_, [**2b‐BRs**](OTf)_12_ and of the mixture [**3b**](OTf)_2_ and [**3b‐IL**](OTf)_4_.

Herein, we report discrete and soluble Borromean rings [**2b‐BRs**] (OTf)_12_ and a [2]catenane [**3b‐IL**] (OTf)_4_. Both architectures are assembled from a dinuclear Pt^II^ complex featuring a benzobiscarbene ligand between the metal centers *syn‐*[Pt_2_(**1b**)(OTf)_2_] and dipyridyl ligands **L^2^** or **L^3^** (Scheme 1). The combination of Pt^II^ with NHC donors leads to a high‐energy triplet state upon irradiation. However, the formation of the Borromean rings is dominated by π···π stacking those results in severe phosphorescence quenching. Conversely, the [2]catenane is assembled via solvophobic effects that simultaneously restrict intramolecular vibrations through mechanical interlocking. Thereby non‐radiative decay associated with π···π stacking interactions is avoided and topology‐driven phosphorescence enhancement is realized.

## 2 Results and Discussion

We have previously described a strategy for the construction of organometallic interlocked architectures using dinuclear CpNi‐dicarbene‐NiCp (Cp = cyclopentadienyl) building blocks along with additional dipyridyl ligands [23]. Motivated by the potential of organometallic MIMs as functional luminescent materials and aiming to elucidate the relationship between phosphorescent properties and topological structure, we envisioned incorporating Pt^II^(ppy)‐dicarbene‐Pt(ppy) (ppy = 2‐phenylpyridinate) units into this system. Pioneering work on this kind of binuclear Pt^II^‐NHC complexes was conducted by Poyatos and coworkers. They prepared a binuclear Pt^II^ complex featuring a pyrene‐bridged bis‐NHC ligand and Pt^II^(ppy) units (Figure S57) [59]. Nevertheless, this complex was found to be non‐emissive. One possible reason could be the low triplet excited state (*T*_1_) energy of the pyrene bridge, which functions as an energy sink. As the subsequent radiative decay from the organic moiety is spin‐forbidden and inefficient, the trapped energy is dissipated primarily through non‐radiative pathways, effectively quenching potential phosphorescence (Figure S57). Inspired by this, we hypothesized that phosphorescence could be activated by blocking this quenching channel. Specifically, we proposed that by replacing the pyrene ‘energy sink’ with a high‐*T*_1_‐energy linker, such as a phenyl‐bridge, the deleterious energy transfer could be mitigated. This would confine the excitation energy on the Pt^II^ centre, thereby promoting decay via the desired phosphorescent pathway (Figure S57).

**FIGURE 1:**
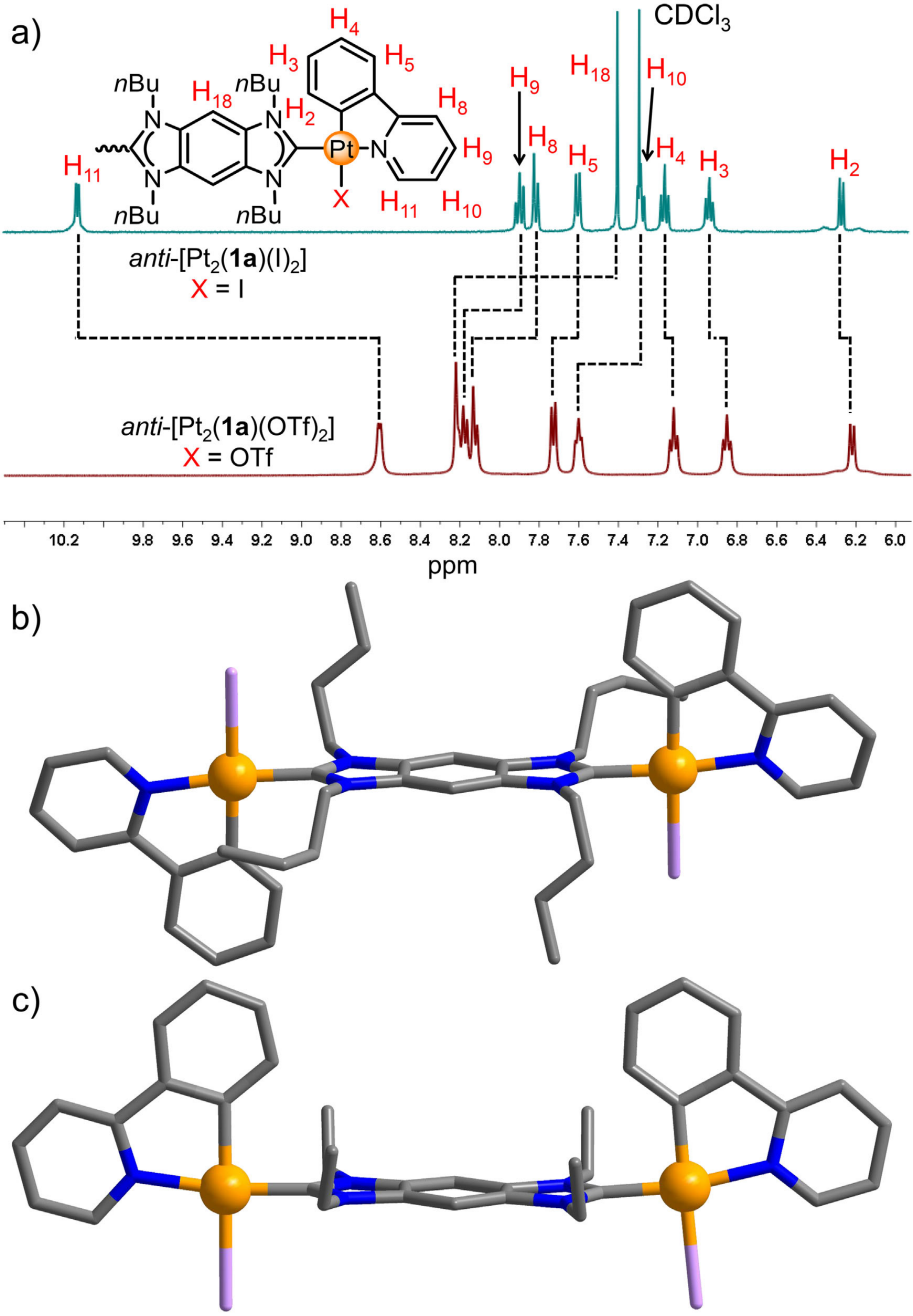
(a) Partial ^1^H NMR spectra of *anti*‐[Pt_2_(**1a**)(I)_2_] (CDCl_3_, 400 MHz, [2.0 mM]) and *anti*‐[Pt_2_(**1a**)(OTf)_2_] (CD_3_OD, 400 MHz, [8.0 mM]). (b) Molecular structure of *anti*‐[Pt_2_(**1a**)(I)_2_] (c) Molecular structure of *syn‐*[Pt_2_(**1b**)(I)_2_] (color code: C, grey; N, blue; Pt, orange; I, violet, hydrogen atoms are omitted).

To achieve this, the bis‐imidazolium salt H_2_‐**1a**(I)_2_ featuring a phenyl‐bridge and *n*‐butyl substituents, was reacted with [Pt(ppy)(µ‐Cl)]_2_ in the presence of sodium acetate and sodium iodide (Scheme 1). The crude products were further purified by column chromatography and crystallization, affording the dinuclear Pt^II^ complex *anti*‐[Pt_2_(**1a**)(I)_2_] as yellow crystals in moderate yield of 32%. The ^1^H NMR spectrum of *anti*‐[Pt_2_(**1a**)(I)_2_] displayed only a single set of signals, which was further confirmed by ^13^C{^1^H}, ^1^H‐^1^H COSY, ^1^H‐^13^C HSQC, and ^1^H‐^13^C HMBC spectroscopy (Figures 1a, S2‒S5). Notably, the resonance for proton H11, adjacent to the pyridyl nitrogen atoms appears at *δ* = 10.13 ppm, likely due to hydrogen bonding with the iodide ligand (Figure S1). In addition, the HRMS (ESI, positive ions) spectrum featured prominent peaks at *m/z* = 1207.2749 (calcd. for [Pt_2_(**1a**)(I)]^+^ 1207.2740) and 540.1844 (calcd. for [Pt_2_(**1a**)]^2+^ 540.1845) with matching isotope distribution (Figures S6 and S7).

Yellow crystals of composition *anti*‐[Pt_2_(**1a**)(I)_2_] suitable for an X‐ray diffraction analysis were obtained by slow diffusion of diethyl ether into a dichloromethane/DMSO (v:v = 4:1) solution of the compound. The x‐ray diffraction analysis established the *anti*‐configuration of the two Pt^II^(ppy) moieties (Figure 1b, Table S1) [60]. Each Pt^II^ centre is coordinated in a chelating fashion by the ppy ligand, the NHC ligand and a iodide. Specifically, the pyridyl group is located in *trans*‐position to the NHC ligand, while the phenyl group is placed in *trans*‐position to the iodide. This geometric arrangement is attributed to the strong *trans*‐influence of the carbon donors (carbene ligand and phenyl group), which favors the placement of thermodynamically weaker ligands (pyridyl group and iodide) in the *trans*‐positions. In addition, the distance between the iodide ligand and the pyridyl proton H11 was determined to be 2.96 Å (Figure S58). This distance falls well within the range of hydrogen bonding interactions, thereby rationalizing the significant downfield shift observed for H11 in the ^1^H NMR spectrum.

In previous studies we found, that the steric bulk of *N*‐alkyl substituents of benzobiscarbene ligands can play a pivotal role in determining the stereochemical outcome in their metalation reaction with Pt^II^ or Ni^II^ [61, 62]. We thus extended our investigation to ligand precursor H_2_‐**1b**(I)_2_ featuring *N*‐ethyl substituents instead of the *n*‐butyl groups in H_2_‐**1a**(I)_2_ (Scheme 1). It was hoped that, reduction of the steric bulk of the *N*‐substituents might lead to dinuclear Pt^II^ complexes with *syn‐*configuration of two Pt^II^(ppy) moieties.

The reaction of H_2_‐**1b**(I)_2_ with [Pt(ppy)(µ‐Cl)]_2_, carried out under identical reaction and purification conditions (including crystallization) as those used for the preparation of *anti*‐[Pt_2_(**1a**)(I)_2_], yielded in 46% the binuclear complex *syn*‐[Pt_2_(**1b**)(I)_2_] as yellow crystals (Scheme 1). Formation of the dinuclear complex was confirmed by NMR spectroscopy and HRMS (ESI, positive ions) spectrometry (Figures S14‒S19). Similarly to *anti*‐[Pt_2_(**1a**)(I)_2_], the ^1^H NMR spectrum of *syn‐*[Pt_2_(**1b**)(I)_2_] displayed a single set of signals. The resonance for the proton H11 was recorded downfield at *δ* = 10.13 ppm, which we take as an indication for hydrogen bonding interactions between the iodide ligand and the pyridyl proton H11. The x‐ray diffraction analysis revealed that, the two Pt^II^(ppy) moieties are arranged in *syn‐*conformation (Figures 1c, S61, and Table S2) [60]. We attribute the formation of these distinct isomers to specific crystal packing interactions governed by the size of the alkyl substituents. As evidenced by the packing diagrams, the smaller ethyl groups in *syn*‐[Pt_2_(**1b**)(I)_2_] allow for significant intermolecular π···π stacking between the benzobiscarbene planes, where two molecules adopt an orthogonal crisscross arrangement (Figure S62). This arrangement likely stabilizes the *syn*‐configuration. Due to the *anti*‐conformation observed for *anti*‐[Pt_2_(**1a**)(I)_2_], such π···π stacking interactions are not possible (Figure S60).

In addition, we calculated a structural model where the two Pt(ppy) moieties in cation *anti*‐[Pt_2_(**1a**)(I)_2_] are rotated by 90° to a coplanar arrangement with the benzobiscarbene core. Such rotation results in a prohibitively short distance (∼1.5 Å) between the ppy carbon and alkyl carbon atoms (Figure S59). While due to the flexibility of the *N*‐substituents some rotation about the Pt‒C bond might be possible, the steric clash implies a high energy barrier for such a rotation. We propose that although both *syn‐* and *anti*‐isomers likely coexist in the crude reaction mixture, the crystallization process drives the conversion of the mixtures into a single configuration. The lattice energy and packing forces during the crystallization promote the formation of the most favorable packing mode (*anti* for *anti*‐[Pt_2_(**1a**)(I)_2_] and *syn* for *syn‐*[Pt_2_(**1b**)(I)_2_]). Once formed, the rotational barrier “locks” the configuration, preventing spontaneous isomerization under ambient conditions. This kinetic stability explains the observation of a single, clean set of NMR signals for both the *syn‐* and *anti*‐isomers in solution.

As anticipated, incorporating a phenyl‐bridge into the dicarbene ligands allows both *anti*‐[Pt_2_(**1a**)(I)_2_] and *syn*‐[Pt_2_(**1b**)(I)_2_] to exhibit characteristic green Pt^II^ phosphorescence emission. Their emission spectra both display a maximum emission peak at 514 nm (Figures S48 and S50). While both complexes exhibit broad excitation bands, the excitation maximum for *syn*‐[Pt_2_(**1b**)(I)_2_] (350 nm) is slightly red‐shifted compared to that of *anti‐*[Pt_2_(**1a**)(I)_2_] (345 nm), which can be attributed to the intermolecular π···π stacking interactions observed in the solid state (Figures S48 and S50). The absolute quantum yields (QY) measured for the solid state in air were determined to be 5.09% and 5.24% for *anti*‐[Pt_2_(**1a**)(I)_2_] and *syn*‐[Pt_2_(**1b**)(I)_2_], respectively.

Since iodo ligands are often detrimental to emission efficiency by facilitating non‐radiative decay pathways via the heavy‐atom effect, they were replaced with triflates. Stirring suspensions of *anti*‐[Pt_2_(**1a**)(I)_2_] or *syn*‐[Pt_2_(**1b**)(I)_2_] with 2 equiv. of AgOTf in methanol at ambient temperature afforded *anti*‐[Pt_2_(**1a**)(OTf)_2_] and *syn*‐[Pt_2_(**1b**)(OTf)_2_] in 89% and 91% yield, respectively. Upon substitution of the iodo ligands, the dinuclear complexes became readily soluble in methanol. The formation of *anti*‐[Pt_2_(**1a**)(OTf)_2_] and *syn‐*[Pt_2_(**1b**)(OTf)_2_] was confirmed by NMR spectroscopy and HRMS (ESI, positive ions) spectrometry (Figures S8‒S13 for *anti*‐[Pt_2_(**1a**)(OTf)_2_], Figures S20‒S26 for *syn*‐[Pt_2_(**1b**)(OTf)_2_]). Both complexes display a single set of NMR signals in methanol. The substitution of the iodo ligands eliminated the intramolecular hydrogen bonding. Consequently, the resonance for pyridyl hydrogen atom H11 shifted significantly up field from *δ* = 10.13 to 8.61 ppm (Figures 1a and S8 for *anti*‐[Pt_2_(**1a**)(OTf)_2_], and *δ* = 10.13 to 8.59 ppm for *syn*‐[Pt_2_(**1b**)(OTf)_2_], Figure S20), providing additional evidence for the existence of hydrogen bonding in the iodo complexes.

After removal of the iodo ligands, the emission spectra of *anti*‐[Pt_2_(**1a**)(OTf)_2_] and *syn*‐[Pt_2_(**1b**)(OTf)_2_] are very similar to the spectra of the corresponding iodo complexes, retaining the characteristic Pt^II^ phosphorescence with an emission maximum at 514 nm (Figures S49 and S51). However, the excitation spectra (monitored at *λ*_em_ = 514 nm) showed a red‐shift of the excitation maximum to 400 nm (Figures S49 and S51). Benefiting from the removal of the iodo ligands, the emission intensity was significantly enhanced, and the absolute quantum yields (QY) of *anti*‐[Pt_2_(**1a**)(OTf)_2_] and *syn*‐[Pt_2_(**1b**)(OTf)_2_] increased relative to the iodo complexes to 14.56% and 15.31%, respectively.

**FIGURE 2:**
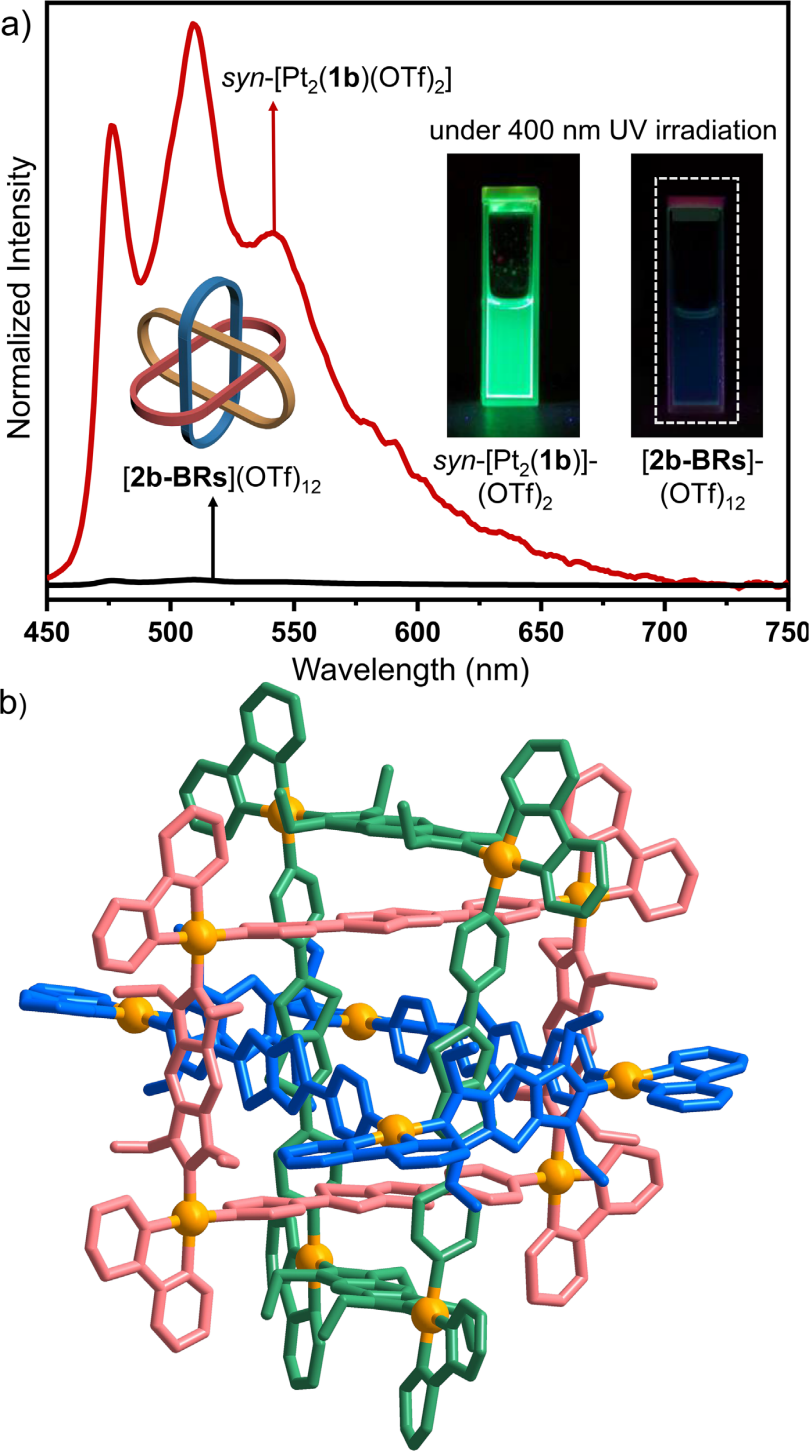
(a) Emission spectra (*λ*_ex_ = 400 nm) of *syn‐*[Pt_2_(**1b**)(OTf)_2_] (CH_3_OH [3.0 mM]) and of [**2b‐BRs**](OTf)_12_ (CH_3_OH, [1.0 mM]). The insets picture the emission of the compounds under 400 nm UV irradiation. (b) Molecular structure of [**2b‐BRs**](OTf)_12_ (the 12 triflate anions and hydrogen atoms have been omitted for clarity and the three tetranuclear metalla rectangles are depicted in different colors).

Although air‐saturated methanol is typically an unfavorable medium for Pt^II^ phosphorescence due to potential oxygen quenching and non‐radiative decay, the methanol solutions of *anti*‐[Pt_2_(**1a**)(OTf)_2_] and *syn*‐[Pt_2_(**1b**)(OTf)_2_] still exhibited a bright green emission under ambient conditions upon excitation at 400 nm (Figures 2a, S52, and S53). However, their excitation bands in methanol both became significantly sharper compared to those in the solid state (still centered at 400 nm), which can be attributed to the disruption of intermolecular packing in solution. In addition, a comparative experiment in degassed methanol revealed a significant enhancement of the emission intensity (Figure S53), thereby further confirming the phosphorescent nature of the emission.

The *syn*‐configuration observed for *syn*‐[Pt_2_(**1b**)(OTf)_2_] is geometrically predisposed for the formation of discrete metalla macrocycles via coordination driven self‐assembly. Together with a second bidentate ligand, potentially phosphorescent mechanically interlocked structures might thus be obtained. We selected the linear ligand **L^2^** (**L^2^** = 2,5‐di(pyridin‐4‐yl) thiazolo[5,4‐*d*] thiazole) for the reaction with *syn*‐[Pt_2_(**1b**)(OTf)_2_]. The rigid thiazolothiazole bridging unit facilitates strong π···π stacking interactions required for interlocking. The removal of the iodo ligands renders the coordination sites of *syn*‐[Pt_2_(**1b**)(OTf)_2_] highly accessible to bipyridyl donors. Consequently, the self‐assembly between *syn*‐[Pt_2_(**1b**)(OTf)_2_] and **L^2^** can be performed by stirring an equimolar mixture of the compounds in methanol overnight. The result of this reaction is an assembly composed of three metalla rectangles interlocked to form the Borromean structure [63, 64] [**2b‐BRs**](OTf)_12_ in 96% yield (Scheme 1).

Formation of [**2b‐BRs**](OTf)_12_ was confirmed by ^1^H, ^13^C, DEPT‐135, ^1^H‐^1^H COSY ^1^H‐^13^C HSQC, and ^1^H‐^13^C HMBC NMR spectroscopy (Figures S27‒S32). In addition, the HRMS spectrum (ESI, positive ions) clearly indicated the formation of the [**2b‐BRs**](OTf)_12_ structure as a trimer of tetranuclear metalla rectangles. The most intense peaks were observed at *m/z* = 1727.0908 (calcd. for [**2b‐BRs**(7OTf)]^5+^ 1727.0500) and 1191.0458 (calcd. for [**2b‐BRs**(5OTf)]^7+^ 1191.0493) with perfectly matching calculated and observed isotopic distribution (Figures S33, S34).

Gratifyingly, yellow crystals of [**2b‐BRs**](OTf)_12_·50MeOH·25H_2_O were obtained in high yield by slow diffusion of isopropyl ether into a methanol/DMSO/DMF solution (ca. v:v:v = 5:1:1) of the compound. The X‐ray diffraction analysis unambiguously confirmed that [**2b‐BRs**](OTf)_12_ adopts the discrete Borromean architecture (Figures 2b, S63, and Table S3) [60]. Three chemically independent rings are arranged in such a way that no two rings are directly interlocked, but the scission of any single ring results in the dissociation of the entire assembly. The planes of the benzobiscarbene ligands are stacked with the planes of the thiazolothiazole moieties of the bipyridyl ligands. The distance between stacked planes measures approximately 3.5 Å, consistent with the conventional distance of π···π stacking interactions (Figure S64). This type of π···π stacking also rationalized the remarkable chemical high‐filed shifts of the pyridyl protons (*δ* = 9.27 and 8.37 ppm, Figure S27) in the ^1^H NMR spectrum of [**2b‐BRs**](OTf)_12_.

Interestingly, although *syn*‐[Pt_2_(**1b**)(OTf)_2_] exhibited intense emission in methanol, [**2b‐BRs**](OTf)_12_ displayed no emission under the same excitation at 400 nm (Figure 2a), indicating significant quenching. This suggests that the strong intramolecular π···π stacking interactions within the Borromean architecture effectively quenched the emission. UV–vis absorption spectroscopy confirmed that [**2b‐BRs**](OTf)_12_ retains significant absorption at 400 nm, thereby ruling out poor excitation efficiency and attributing the quenching solely to the intrinsic π···π interactions (Figure S54). This finding implies that eliminating strong inter‐rings π···π interactions is essential for constructing phosphorescent MIMs.

We have previously demonstrated that solvophobic effects alone are sufficient to drive the formation of mechanically interlocked architectures featuring di‐NHC ligands [23, 24]. To achieve this with *syn*‐[Pt_2_(**1b**)(OTf)_2_], we selected the flexible bipyridyl ligand **L^3^** (Scheme 1) to replace **L^2^**, as the former is less prone to form intramolecular π···π interactions with the Pt^II^ benzobiscarbene building block. Stirring a mixture of *syn*‐[Pt_2_(**1b**)(OTf)_2_] and **L^3^** (1:1) in CD_3_OD/DMSO‐*d*_6_ (v:v = 4:1) for 12 h at ambient temperature yielded a clear yellow solution. The ^1^H NMR spectrum [4.0 mM] revealed a mixture comprising the [2]catenane [**3b‐IL**](OTf)_4_ (major) and the metalla macrocycle [**3b**](OTf)_2_ (minor) (Scheme 1, Figure S35). The coexistence of these two species was further confirmed by ^13^C{^1^H}, DEPT‐135, ^1^H DOSY, ^1^H‐^1^H COSY ^1^H‐^13^C HSQC, and ^1^H‐^13^C HMBC NMR spectroscopy (Figures S36‒S44).

**FIGURE 3:**
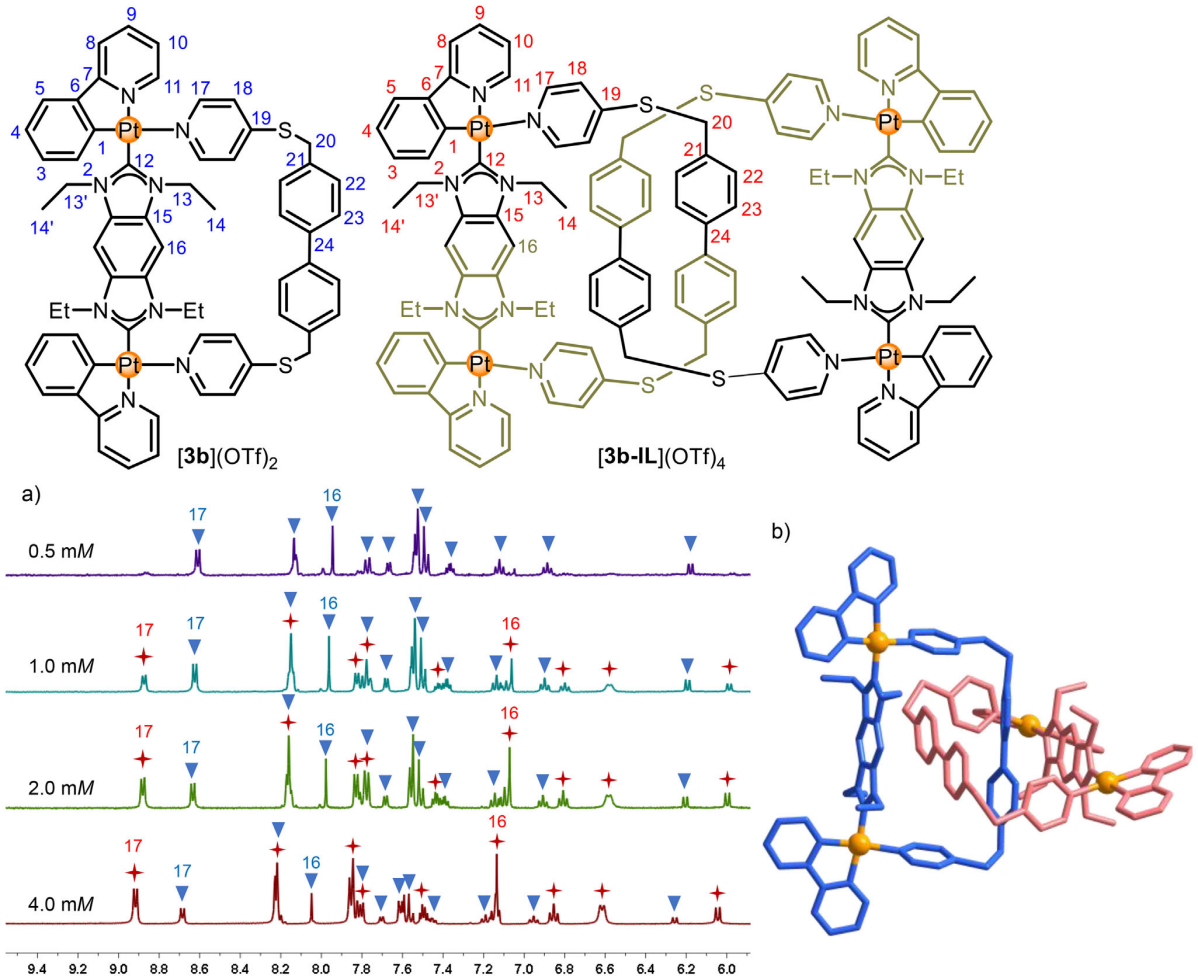
(a) Partial ^1^H NMR spectra of mixtures of [**3b**](OTf)_2_ (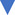) + [**3b‐IL**](OTf)_4_(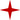) (CD_3_OD/DMSO‐*d*_6_, v:v = 4:1, 400 MHz, [0.5, 1.0, 2.0, 4.0 mM]), showing an increasing amount of [**3b‐IL**](OTf)_4_ in the mixture with increasing concentration (selected resonances are labeled). (b) Molecular structure [**3b‐IL**](OTf)_4_ with each ring depicted in a different color (hydrogen atoms and anions have been omitted).

The chemical shifts of the aromatic protons in [**3b**](OTf)_2_ are similar to those observed for the aromatic protons in the free ligand **L^3^** and *syn*‐[Pt_2_(**1b**)(OTf)_2_], indicating the presence of a metalla macrocycle [**3b**](OTf)_2_. In contrast, selected aromatic protons of [**3b‐IL**](OTf)_4_ exhibit significant shielding effects. For instance, the resonance of the benzobiscarbene protons (H16) shifted from *δ* = 8.05 (in [**3b**](OTf)_2_) to 7.13 (in [**3b‐IL**](OTf)_4_). Even stronger up field shifts were observed for the phenyl protons (H22 and H23) of the bipyridyl ligand (*δ* = 7.59 and 7.57 for [**3b**](OTf)_2_; 6.62 and 5.27 for [**3b‐IL**](OTf)_4_, Figure S35). These pronounced shielding effects are diagnostic for the interlocked topology of [**3b‐IL**](OTf)_4_ [23]. Furthermore, the product distribution in the mixture is dependent on the concentration. The ^1^H NMR spectrum at the low concentration of 0.5 mM shows exclusively the metalla macrocycle **3b**(OTf)_2_, whereas increasing the concentration (1.0, 2.0, and 4.0 mM) progressively favors the formation of the [2]catenane [**3b‐IL**](OTf)_4_ (Figures 3a and S47).

Yellow single crystals of [**3b‐IL**](OTf)_4_·10MeOH·6H_2_O were obtained by slow diffusion of isopropyl ether into a methanol/DMSO/DMF solution (ca. v:v:v = 5:1:1) of the mixture. The X‐ray diffraction analysis unambiguously confirmed the discrete interlocked [2]catenane topology of [**3b‐IL**](OTf)_4_ (Figure 3b, S65, and Table S4), [60] which is further supported by HRMS spectroscopy (ESI, positive ions, Figures S45 and S46). As expected, no intramolecular π···π stacking interactions were observed in the molecular structure of [**3b‐IL**](OTf)_4_. The biphenyl units from the two bipyridyl ligands adopt a crossed arrangement that precludes mutual π···π interactions (Figures 3b and S65). In addition, the benzobiscarbene planes are not oriented parallel to the biphenyl groups (Figure S66). Given the apparent absence of π···π stacking, the main driving forces for formation of the [2]catenane are attributed primarily to solvophobic effects where, insertion of a biphenyl ring into one macrocycle each leads to the interlocked structure and prevents interactions with the polar solvents MeOH and DMSO‐*d*_6_ [23].

**FIGURE 4:**
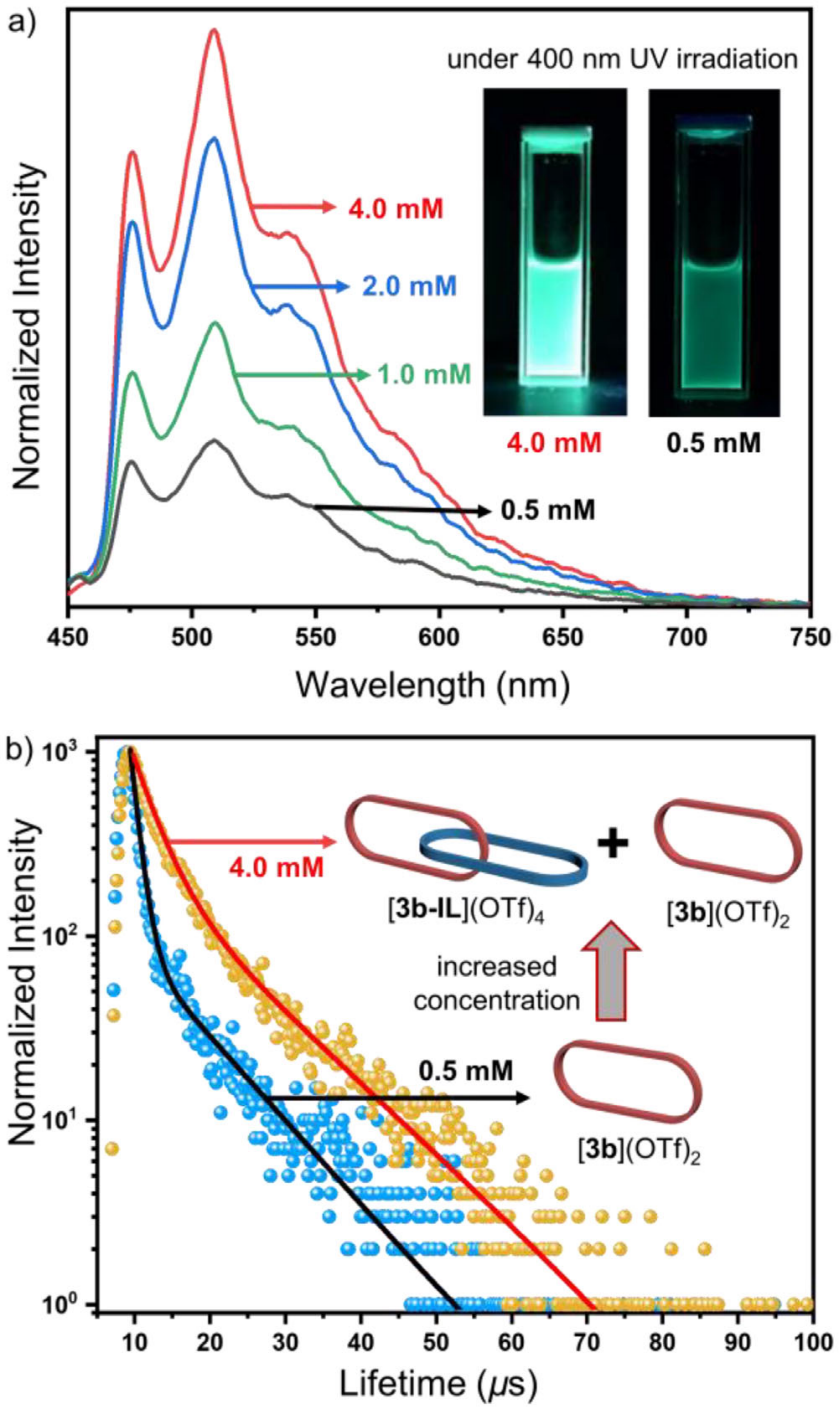
(a) Emission spectra (*λ*_ex_ = 400 nm) of the mixture [**3b**](OTf)_2_ + [**3b‐IL**](OTf)_2_ in CH_3_OH/DMSO (v/v = 4:1, [4.0, 2.0, 1.0, and 0.5 mM]. The insets picture the emission of the compounds under 400 nm UV irradiation. (b) Decay curve (*λ*_ex_ = 400 nm) of [**3b**](OTf)_2_ + [**3b‐IL**](OTf)_4_ (CH_3_OH/DMSO, v:v = 4:1, [4.0 and 0.5 mM].

To elucidate the impact of the topological structure on the photophysical properties, we performed luminescence studies on the mixture [**3b**](OTf)_2_ and [**3b‐IL**](OTf)_4_. At low concentrations (0.5 mM), the mixture contains almost exclusively the metalla macrocycle [**3b**](OTf)_2_, which exhibits characteristic Pt^II^ phosphorescence. Upon excitation at 400 nm, its emission profile features a broad band centered at 514 nm (Figure 4a), closely resembling that of the building block *syn*‐[Pt_2_(**1b**)](OTf)_2_ (Figure S53). The excitation spectrum (monitored at *λ*_em_ = 514 nm) also displays a maximum at 400 nm (Figure S55), confirming that the luminescence primarily originates from the Pt^II^ phosphorescence. As the concentration increases, [**3b**](OTf)_2_ gradually transforms into the interlocked [2]catenane [**3b‐IL**](OTf)_4_, accompanied by a dramatic enhancement in emission intensity. At the concentration of 4.0 mM, the solution remains clear (no precipitation) and emits intense green light clearly visible to the naked eye (Figure 4a). In contrast, the luminescent building block *syn*‐[Pt_2_(**1b**)(OTf)_2_] exhibits typical aggregation‐caused quenching (ACQ) in methanol, where higher concentrations lead to diminished emission (Figure S56). This underscores that the formation of the mechanically interlocked structure is pivotal for phosphorescence enhancement. This finding is further supported by the luminescence lifetimes, which extend from 4.2 µs (for 0.5 mM [**3b**](OTf)_2_) to 7.5 µs (for 4.0 mM [**3b‐IL**](OTf)_4_ + [**3b**](OTf)_2_, Figure 4b). These results suggest that the mechanical connection in [**3b‐IL**](OTf)_4_ effectively restricts intramolecular rotations and vibrations. Moreover, since the formation of the mechanical interlocked structure [**3b‐IL**](OTf)_4_ is driven by solvophobic effects rather than π···π interactions, the stacking‐induced quenching observed in the Borromean rings [**2b‐BRs**](OTf)_12_ is avoided and significant phosphorescence enhancement is realized. Crucially and in contrast to many reported Pt^II^‐based AIPE systems that, relay on the formation of insoluble aggregates or nanoparticles, the strategy presented here achieves emission enhancement within a discrete, soluble molecular architecture.

## 3 Conclusion

We have prepared discrete, soluble, and highly phosphorescent MIMs by employing dinuclear benzobiscarbene Pt^II^ building blocks. The *N*‐substituents on the benzobiscarbene ligand dictate the stereochemical outcome of the metalation reaction with Pt^II^, allowing selective access to the *syn*‐configured complex *syn*‐[Pt_2_(**1b**)(OTf)_2_] essential for use in supramolecular self‐assembly reactions. In addition, the fundamental relationship between supramolecular driving forces, topological structures, and phosphorescent properties of Borromean rings and [2]catenanes obtained from benzobiscarbene bridged dinuclear platinum complex *syn*‐[Pt_2_(**1b**)(OTf)_2_] and specifically tailored bipyridyl ligands has been elucidated. By utilizing distinct driving forces, two types of discrete MIMs were constructed. Borromean rings [**2b‐BRs**](OTf)_12_ were obtained and stabilized by π···π stacking interactions, while the driving force for the formation of the [2]catenane [**3b‐IL**](OTf)_4_ were solvophobic effects. While the π···π stacking interactions in the Borromean rings lead to severe luminescence quenching, the solvophobically assembled [2]catenane effectively circumvents these deleterious interactions while simultaneously restricting intramolecular rotations and vibrations. Consequently, the [2]catenane exhibits dramatic phosphorescence enhancement compared to the Borromean rings. This study not only provides new insights into the design of functional MIMs, but also offers a novel strategy for AIE enhancement.

## Conflicts of Interest

The authors declare no conflict of interest.

## Supporting information




**Supporting File 1**: anie71846‐sup‐0001‐SuppMat.pdf.

## Data Availability

The data that support the findings of this study are available in the Supporting Information of this article.
