# Supplementary material for: An Organometallic [2]Catenane With Pt‒(di‐NHC)‒Pt Units: A Topology‐Driven Strategy for Enhanced Phosphorescence in Discrete Aggregates
Source: Angew Chem Int Ed Engl. 2026 Mar 23;65(19):e1981919. doi: 10.1002/anie.1981919 (PMC13134634; doi:10.1002/anie.1981919)
Supplement: Supplementary file 1 — Supporting File 1: anie71846‐sup‐0001‐SuppMat.pdf. [file ANIE-65-e1981919-s001.pdf]

Table of Contents

1. Experimental procedures S1

2. NMR and ESI HRMS spectra S7

3. Photophysical performance S31

4. X-ray diffraction studies S36

5. References S47

1. Experimental procedures

General Procedures. All manipulations were carried out under an argon atmosphere unless stated otherwise. ^{1}H and $^{13}C\{^{1}H\}$ NMR spectra were measured on a Bruker ASCEND-TM 400 spectrometer at ambient temperature (295 K). Chemical shifts (δ) are expressed in ppm relative to $SiMe_4$ using the residual protonated solvent signal as an internal standard. For the assignments of the NMR resonances see the numbering at the molecular plots. Coupling constants are expressed in Hz. Mass spectra were obtained with reflex an Orbitrap LTQ XL spectrometer (Thermo Scientific). Absorption spectra were recorded on a Lambd650 UV/vis spectrophotometer. Luminescence excitation and emission spectra were obtained on an Edinburgh FLS980 spectrophotometer. Luminescence lifetime measurements were carried out on an Edinburgh FLS920 phosphorimeter using a microsecond pulse lamp as excitation source. The life time data were obtained from fitting the experiment luminescent decay. Absolute quantum yield (QY) measurements were made by exciting the solid samples with diffuse light within an integrating sphere. [Pt(ppy)(μ-Cl)$_2$]$_2$,[S1] H_2-**1a**(I)$_2$,[S2] H_2-**1b**(I)$_2$, [S2] **L²** (**L²** = 2,5-di(pyridin-4-yl)thiazolo[5,4-*d*]thiazole)[S3] and **L³** (**L³** = 4,4'-bis((1*H*-imidazol-1-yl)methyl)-1,1'-biphenyl)[S2] were prepared as previously described.

Preparation of *anti*-[Pt$_2$(1a)(I)$_2$]

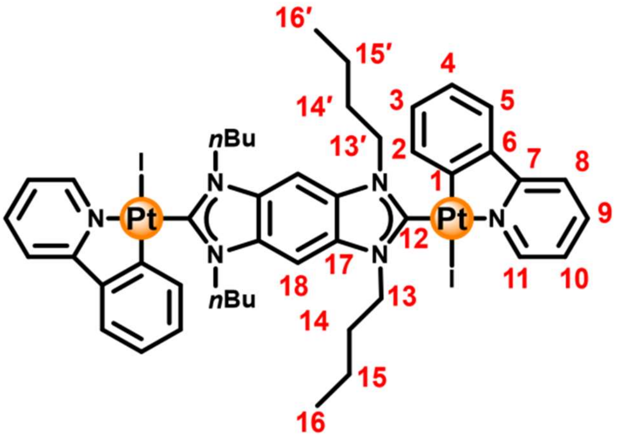

A solution of benzimidazolium salt H_2-**1a**(I)$_2$ (319 mg, 0.5 mmol), [Pt(ppy)(μ-Cl)]$_2$ (385 mg, 0.5 mmol), NaOAc (82 mg, 1.0 mmol) and NaI (600 mg, 4.0 mmol) in DMF (50 mL) was heated at 105℃ overnight. The solvents were removed *in vacuo*. The solid residue was extracted with dichloromethane. The raw product was further purified by column chromatography (SiO_2, dichloromethane) and crystallization by vapor diffusion of diethyl ether into a dichloromethane solution. The dinuclear complex *anti*-[Pt$_2$(**1a**)(I)$_2$] was obtained as yellow crystalline solid. Yield: 213 mg (0.16 mmol, 32%). ^{1}H NMR (400 MHz, $CDCl_3$): δ =

10.13 (d, J = 8.0 Hz, 2H, H11), 7.89 (t, J = 8.0 Hz, 2H, H9), 7.81 (d, J = 8.0 Hz, 2H, H8), 7.60 (d, J = 7.2 Hz, 2H, H5), 7.40 (s, 2H, H18), 7.28 (t, J = 8.0 Hz, 2H, H10), 7.16 (t, J = 7.2 Hz, 2H, H4), 6.93 (t, J = 7.2 Hz, 2H, H3), 6.26 (d, J = 7.2 Hz, 2H, H2), 4.81 (m, 4H, H13), 4.66 (m, 4H, H13'), 2.16 (m, 4H, H14), 1.99 (m, 4H, H14'), 1.45 (m, 8H, H15, H15') 0.93 (t, J = 7.2 Hz, 12H, H16, H16'). $^{13}C\{^1H\}$ NMR (100 MHz, $CDCl_3$): δ = 172.14 (C12), 165.26 (C7), 154.16 (C11), 146.43 (C1), 144.87 (C6), 138.38 (C9), 134.09 (C2), 131.65 (C17), 130.74 (C3), 123.90 (C5), 123.49 (C4), 123.20 (C10), 118.44 (C8), 91.78 (C18), 48.57 (C13, C13'), 30.50 (C14, C14'), 20.40 (C15, C15'), 13.79 (C16, C16'). HRMS (ESI, positive ions): m/z = 1207.2749 (calcd for *anti*-$[Pt_2(\mathbf{1a})(I)]^+$ 1207.2740), 540.1844 (calcd for *anti*-$[Pt_2(\mathbf{1a})]^{2+}$ 540.1845).

Preparation of *anti*-[Pt₂(1a)(OTf)₂]

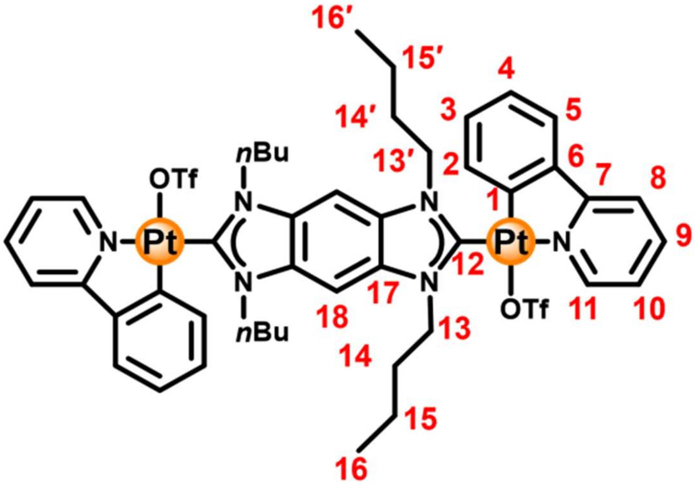

A solution of *anti*-$[Pt_2(\mathbf{1a})(I)_2]$ (133.5 mg, 0.10 mmol) and AgOTf (51.4 mg, 0.20 mmol) in CH_2Cl_2 (30 mL) was stirred at ambient temperature for 12 h. Subsequently, insoluble compounds (AgI) were removed by filtration. The filtrate was concentrated to a volume of 3 mL under reduced pressure. Slow diffusion of diethyl ether into the concentrated filtrate yielded *anti*-$[Pt_2(\mathbf{1a})(OTf)_2]$ as a white powder. Yield: 122.7 mg (0.089 mmol, 89%). ^1H NMR (400 MHz, CD_3OD): δ = 8.61 (d, J = 8.0 Hz, 2H, H11), 8.22 (s, 2H, H18), 8.18 (t, J = 8.0 Hz, 2H, H9), 8.12 (d, J = 8.0 Hz, 2H, H8), 7.73 (d, J = 7.6 Hz, 2H, H5), 7.60 (t, J = 8.0 Hz, 2H, H10), 7.12 (t, J = 7.6 Hz, 2H, H4), 6.86 (t, J = 7.6 Hz, 2H, H3), 6.22 (d, J = 7.6 Hz, 2H, H2), 5.02 (m, 4H, H13), 4.82 (m, 4H, H13'), 2.20 (m, 4H, H14), 1.99 (m, 4H, H14'), 1.52 (m, 8H, H15, H15'), 0.93 (t, J = 8.0 Hz, 12H, H16, H16'). $^{13}C\{^1H\}$ NMR (100 MHz, CD_3OD): δ = 171.17 (C12), 164.02 (C7), 146.34 (C11), 144.55 (C6), 140.67 (C9), 135.99 (C2), 131.91 (C17), 130.11 (C3), 128.54 (C1), 124.59 (C4), 124.10 (C5), 123.10 (C10), 121.96 (OTf^-),119.28 (C8), 118.79 (OTf^-), 93.66 (C18), 48.49 (C13, C13'), 31.31 (C14, C14'), 19.73 (C15, C15'), 12.65

(C16, C16’). HRMS (ESI, positive ions): *m/z* = 1229.3164 (calcd for *anti*-[Pt_2(**1a**)(OTf)]$^+$ 1229.3215).

Preparation of *syn*-[Pt_2(1b)(I)$_2$]

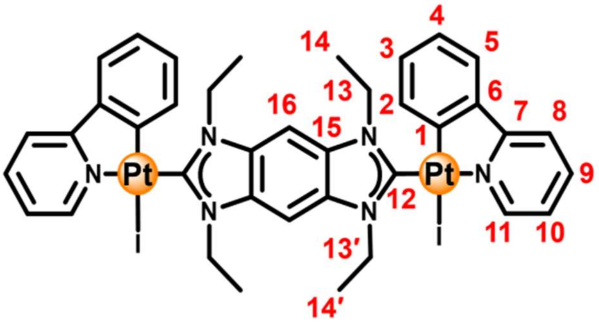

A solution of benzimidazolium salt H_2-**1b**(I)$_2$ (263 mg, 0.5 mmol), [Pt(ppy)(μ-Cl)]$_2$ (385 mg, 0.5 mmol), NaOAc (82 mg, 1.0 mmol) and NaI (600 mg, 4.0 mmol) in DMF (50 mL) was heated at 105 ℃ overnight. The solvents were removed *in vacuo*. The solid residue was extracted with dichloromethane. The raw product was further purified by column chromatography (SiO_2, dichloromethane). Crystallization by vapor diffusion of diethyl ether into a dichloromethane solution yielded the dinuclear complex *syn*-[Pt_2(**1b**)(I)$_2$] as a yellow crystalline solid. Yield: 281 mg (0.23 mmol, 46%). ^{1}H NMR (400 MHz, $CDCl_3$): δ = 10.13 (d, *J* = 8.0 Hz, 2H, H11), 7.86 (t, *J* = 8.0 Hz, 2H, H9), 7.78 (d, *J* = 8.0 Hz, 2H, H8), 7.73 (s, 2H, H16), 7.57 (d, *J* = 7.6 Hz, 2H, H5), 7.25 (t, *J* = 8.0 Hz, 2H, H10), 7.12 (t, *J* = 7.6 Hz, 2H, H4), 6.87 (t, *J* = 7.6 Hz, 2H, H3), 6.24 (d, *J* = 7.6 Hz, 2H, H2), 5.06 (m, 4H, H13), 4.71 (m, 4H, H13’), 1.48 (t, *J* = 7.2 Hz, 12H, H14, H14’). ^{13}C{^{1}H} NMR (100 MHz, $CDCl_3$): δ = 170.65 (C12), 165.27 (C7), 154.07 (C11), 146.10 (C1), 145.13(C6), 138.46 (C9), 134.92 (C2), 130.77 (C15), 130.31 (C3), 123.98 (C5), 123.51 (C4), 123.27 (C10), 118.51 (C8), 92.06 (C16), 44.00 (C13, C13’), 13.68 (C14, C14’). HRMS (ESI, positive ions): *m/z* = 1095.1493 (calcd for *syn*-[Pt_2(**1b**)(I)]$^+$ 1095.1487).

Preparation of *syn*-[Pt_2(1b)(OTf)$_2$]

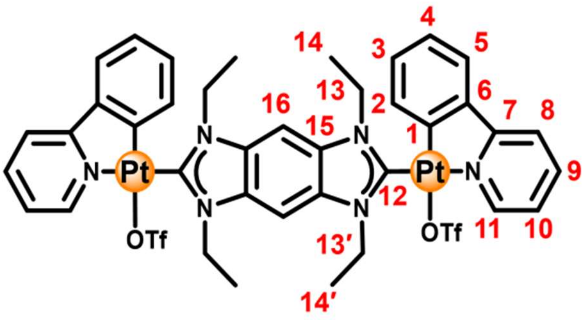

A solution of *syn*-[Pt_2(**1b**)(I)$_2$] (122.3 mg, 0.10 mmol) and AgOTf (51.4 mg, 0.20 mmol) in dichloromethane (30 mL) stirred at ambient temperature for 12h. Subsequently, insoluble compounds (AgI) were removed by filtration. The filtrate was concentrated to a volume of 3 mL under reduced pressure. Slow diffusion of diethyl ether into the concentrated filtrate yielded

syn-[Pt_2(**1b**)$(OTf)_2$] as a white powder. Yield: 115.3 mg (0.091 mmol, 91%). ^{1}H NMR (400 MHz, CD_3OD): δ = 8.59 (d, J = 8.0 Hz, 2H, H11), 8.27 (s, 2H, H16), 8.16 (t, J = 8.0 Hz, 2H, H9), 8.10 (d, J = 8.0 Hz, 2H, H8), 7.70 (d, J = 7.6 Hz, 2H, H5), 7.58 (t, J = 8.0 Hz, 2H, H10), 7.09 (t, J = 7.6 Hz, 2H, H4), 6.81 (t, J = 7.6 Hz, 2H, H3), 6.14 (d, J = 7.6 Hz, 2H, H2), 5.12 (m, 4H, H13), 4.92 (m, 4H, H13'), 1.61 (t, J = 7.2 Hz, 12H, H14, H14'). ^{13}C{^{1}H} NMR (100 MHz, CD_3OD): δ = 170.37 (C12), 163.99 (C7), 146.28 (C11), 144.66 (C6), 140.67(C9), 136.16 (C2), 131.46 (C15), 130.07 (C3), 128.36 (C1), 124.58 (C4), 124.14 (C5), 123.09 (C10), 121.98 (OTf^-), 119.26 (C8), 118.81 (OTf^-), 93.69 (C16), 43.91 (C13, C13'), 13.41 (C14, C14'). HRMS (ESI, positive ions): m/z = 1117.1886 (calcd for *syn*-[Pt_2(**1b**)(OTf)]$^+$ 1117.1961), 484.1060 (calcd for *syn*-[Pt_2(**1b**)]$^{2+}$ 484.1218).

Preparation of Borromean rings [2b-BRs]$(OTf)_{12}$

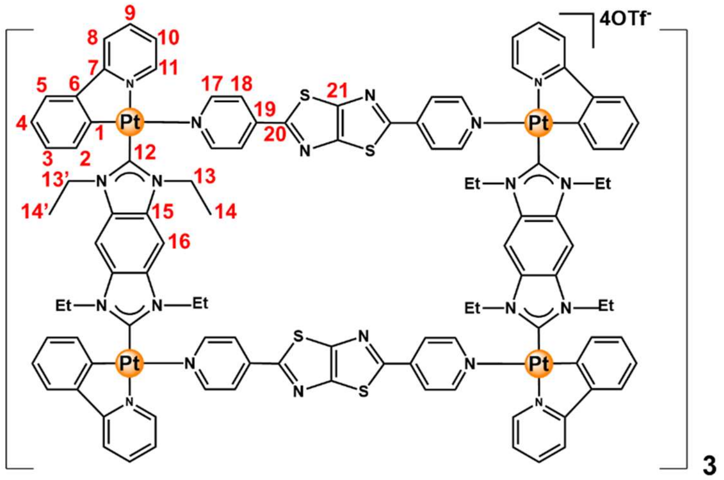

To a solution of *syn*-[Pt_2(**1b**)$(OTf)_2$] (50.68 mg, 0.04 mmol) in methanol (20 mL) was added 2,5 di(pyridin-4-yl)thiazolo- [5,4-d]thiazole (**L^2**) (11.84 mg, 0.04 mmol) suspended in dichloromethane (5 mL). The mixture was stirred at ambient temperature for 12 h. Subsequently, the solution was concentrated to a volume of 3 mL under reduced pressure. Slow diffusion of diethyl ether into the concentrated filtrate yielded Borromean rings [**2b-BRs**]$(OTf)_{12}$ as a yellow powder. Yield: 60.04 mg (0.0064 mmol, 96%). ^{1}H NMR (400 MHz, CD_3OD:DMSO-d_6 v:v = 4:1): δ = 9.27 (d, J = 7.2 Hz, 24H, H17), 8.37 (d, J = 7.2 Hz, 24H, H18), 8.26–8.21 (m, 12H, H11; 12H, H16; 12H, H8), 7.89 (d, J = 7.6 Hz, 12H, H5), 7.83 (m, 12H, H9), 7.43 (t, J = 5.6 Hz, 12H, H10), 7.23 (t, J = 7.6 Hz, 12H, H4), 7.00 (t, J = 7.6 Hz, 12H, H3), 6.32 (d, J = 7.6 Hz, 12H, H2), 5.30 (q, J = 8.0 Hz, 24H, H13), 4.99 (q, J = 8.0 Hz, 24H, H13'), 1.56 (t, J = 8.0 Hz, 72H, H14, H14'). ^{13}C{^{1}H} NMR (100 MHz, CD_3OD/DMSO-d_6 v:v = 4:1): δ = 169.20 (C12), 166.28 (C20), 165.44 (C7), 153.84 (C21), 152.93 (C17), 147.49 (C9), 145.88 (C6), 142.46 (C19), 140.85 (C11), 138.23 (C1), 137.07 (C2), 131.26 (C15), 130.33 (C3), 124.97 (C4), 124.49 (C5), 123.80 (C10),

123.54 (C18), 119.85 (C8), 94.04 (C16), 44.06 (C13, C13’), 13.67 (C14, C14’). HRMS (ESI, positive ions): *m/z* = 1727.0908 (calcd for [**2b-BRs**(7OTf)]$^{5+}$ 1727.0500), 1191.0458 (calcd for [**2b-BRs**(5OTf)]$^{7+}$ 1191.0493).

Preparation of the mixture of metallarectange [3b](OTf)$_2$ and [2]catenane [3b-IL](OTf)$_4$

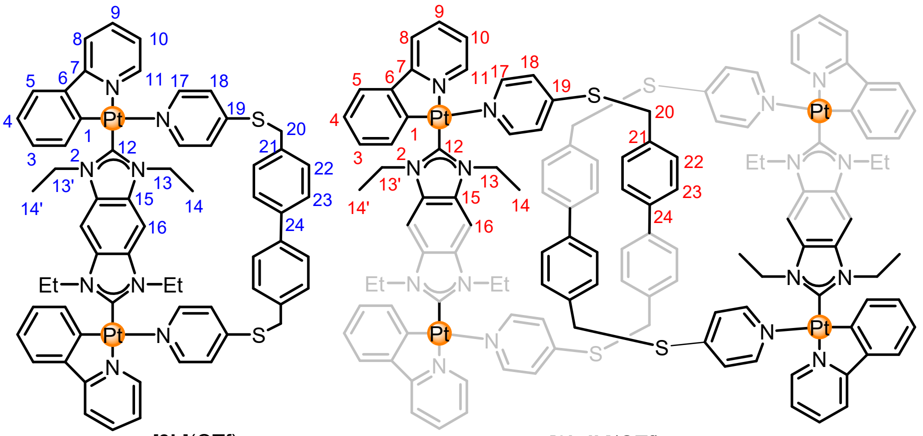

[**3b**](OTf)$_2$ [**3b-IL**](OTf)$_4$

To a solution of *syn*-[Pt$_2$(**1b**)(OTf)$_2$] (50.68 mg, 0.04 mmol) in methanol (20 mL) was added 4,4’-bis((pyridin-4-ylthio)-methyl)-1,1’-biphenyl (**L^3**) (16.0mg, 0.04 mmol) suspended in CH$_2$Cl$_2$ (5 mL or DMSO-*d*$_6$). The suspension was stirred at ambient temperature for 12 h. Subsequently, the solution was concentrated to a volume of 3 mL under reduced pressure. Slow diffusion of diethyl ether into the concentrated solution yielded a mixture of [2]catenane [**3b-IL**](OTf)$_4$ and the metallarectangle [**3b**](OTf)$_2$ as a white powder. Yield: 62.70 mg (0.0375 mmol, 94% relative to metallarectangle [**3b**](OTf)$_2$).

^{1}H NMR (400 MHz, CD$_3$OD/DMSO-*d*$_6$ v:v = 4:1): *δ* (for [**3b**](OTf)$_2$) = 8.69 (d, *J* = 8.0 Hz, 4H, H17), 8.23–8.20 (m, 4H, H8; 4H, H11), 8.05 (s, 2H, H16), 7.82 (d, *J* = 4.0 Hz, 2H, H5), 7.70 (m, 2H, H9), 7.62–7.56 (m, 8H, H18; 8H, H22; 8H, H23), 7.46 (m, 2H, H10), 7.20 (m, 2H, H4), 6.95 (t, *J* = 8.0 Hz, 2H, H3), 6.26 (d, *J* = 8.0 Hz, 2H, H2), 5.13 (m, 4H, H13), 4.83 (m, 4H, H13’), 4.52 (s, 4H, H20), 1.46 (t, *J* = 8.0 Hz, 12H, H14, H14’); *δ* (for [**3b-IL**](OTf)$_4$) = 8.92 (d, *J* = 8.0 Hz, 8H, H17), 8.23 (m, 4H, H8; 4H, H11), 7.86–7.84 (m, 8H, H18; 4H, H5), 7.81 (m, 4H, H9), 7.50 (t, *J* = 4.0 Hz, 4H, H10), 7.17–7.12 (m, 4H, H4; 4H, H16), 6.85 (t, *J* = 8.0 Hz, 4H, H3), 6.62 (d, *J* = 7.6 Hz, 8H, H22), 6.05 (d, *J* = 8.0 Hz, 4H, H2), 5.27 (d, *J* = 7.6 Hz, 8H, H23), 4.95 (m, 8H, H13),4.46 (s, 8H, H20), 4.30 (m, 8H, H13’), 1.34 (t, *J* = 8.0 Hz, 24H, H14, H14’).

^{13}C{^{1}H} NMR (100 MHz, CD$_3$OD/*d*$_6$-DMSO v:v = 4:1): *δ* (for [**3b**](OTf)$_2$) = 169.12 (C12), 165.49 (C7), 153.94 (C19), 150.47 (C17), 147.36 (C9), 145.87 (C6), 140.72 (C11), 138.74

(C1), 137.11 (C2), 135.85 (C21), 135.75 (C24), 131.11 (C15), 130.17 (C3), 129.03 (C22), 126.61 (C23), 124.76 (C4), 124.38 (C5), 123.84 (C18), 123.65 (C10), 119.84 (C8), 119.18 (OTf^-), 94.00 (C16), 43.83 (C13, C13'), 33.23 (C20), 13.54 (C14, C14'); δ (for [**3b-IL**]$(OTf)_4$) = 168.53 (C12), 165.49 (C7), 154.58 (C19), 150.58 (C17), 147.36 (C9), 145.87 (C6), 140.80 (C11), 138.80 (C1), 137.11 (C2), 135.75 (C24), 134.63 (C21), 131.11 (C15), 130.40 (C3), 127.62 (C22), 124.76 (C4), 124.38 (C5), 124.22 (C23), 123.84 (C18), 123.65 (C10), 122.36 (OTf^-), 119.84 (C8), 92.77 (C16), 43.83 (C13, C13'), 33.08 (C20), 13.81 (C14, C14').

HRMS (ESI, positive ions): *m/z* = 1518.3289 (calcd for [**3b**(OTf)]$^+$, 1518.3045 or [**3b-IL**(2OTf)]$^{2+}$ 1518.3042), 962.5541 (calcd for [**3b-IL**(OTf)]$^{3+}$ 962.5520). The peak at *m/z* = 962.5541 clearly shows the presence of the [2]catenane [**3b-IL**]$(OTf)_4$ in the mixture.

2. NMR and ESI-MS spectra

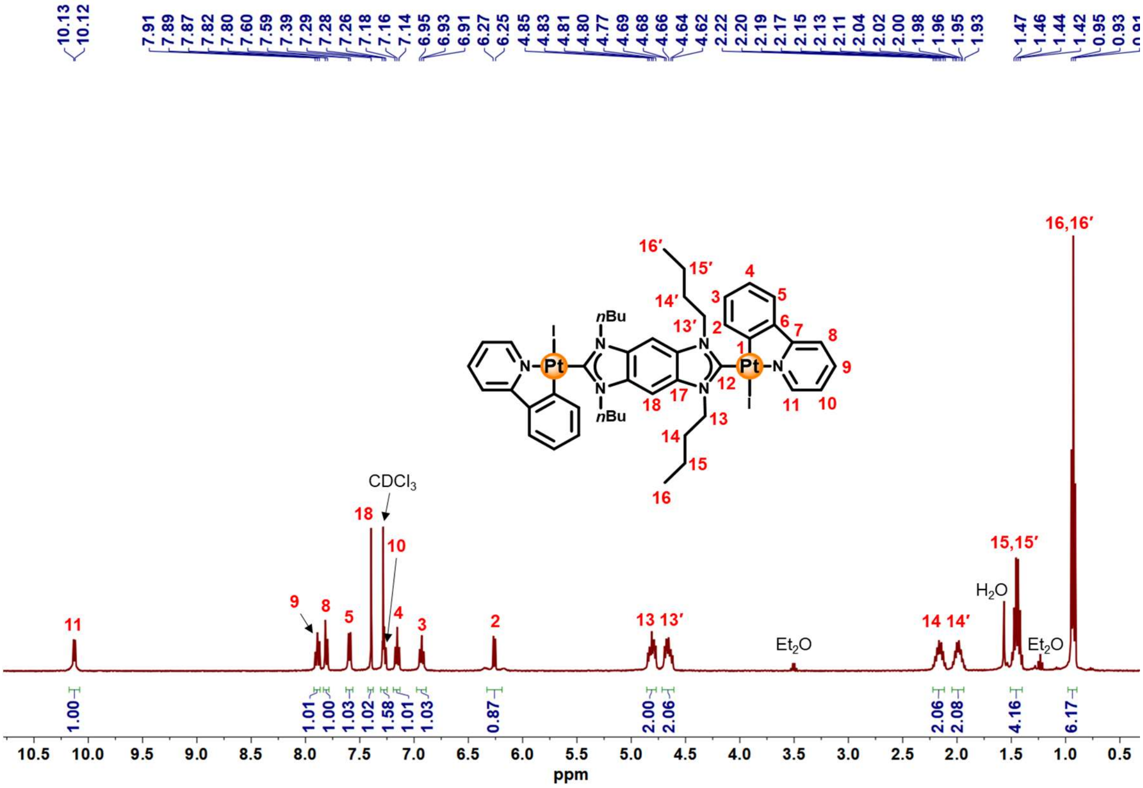

Figure S1. ^{1}H NMR spectrum of *anti*-[Pt$_2$(**1a**)(I)$_2$] ($CDCl_3$, 400 MHz, [2.0 m*M*]).

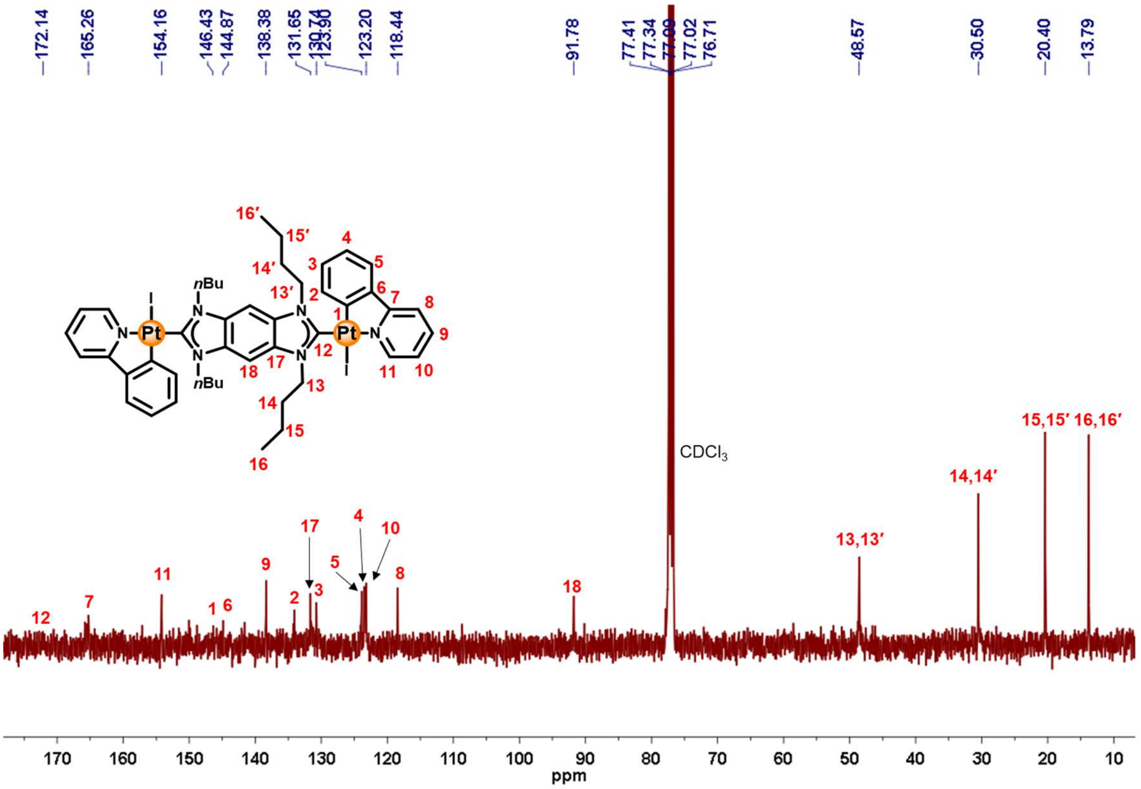

Figure S2. ^{13}C{^{1}H} NMR spectrum of *anti*-[Pt$_2$(**1a**)(I)$_2$] ($CDCl_3$, 100 MHz, [2.0 m*M*]). Due to poor solubility, the ^{13}C signals of C12 and C1 were difficult to observe directly. However, their chemical shifts were unambiguously assigned via ^{1}H-^{13}C HMBC spectroscopy (Figure S5).

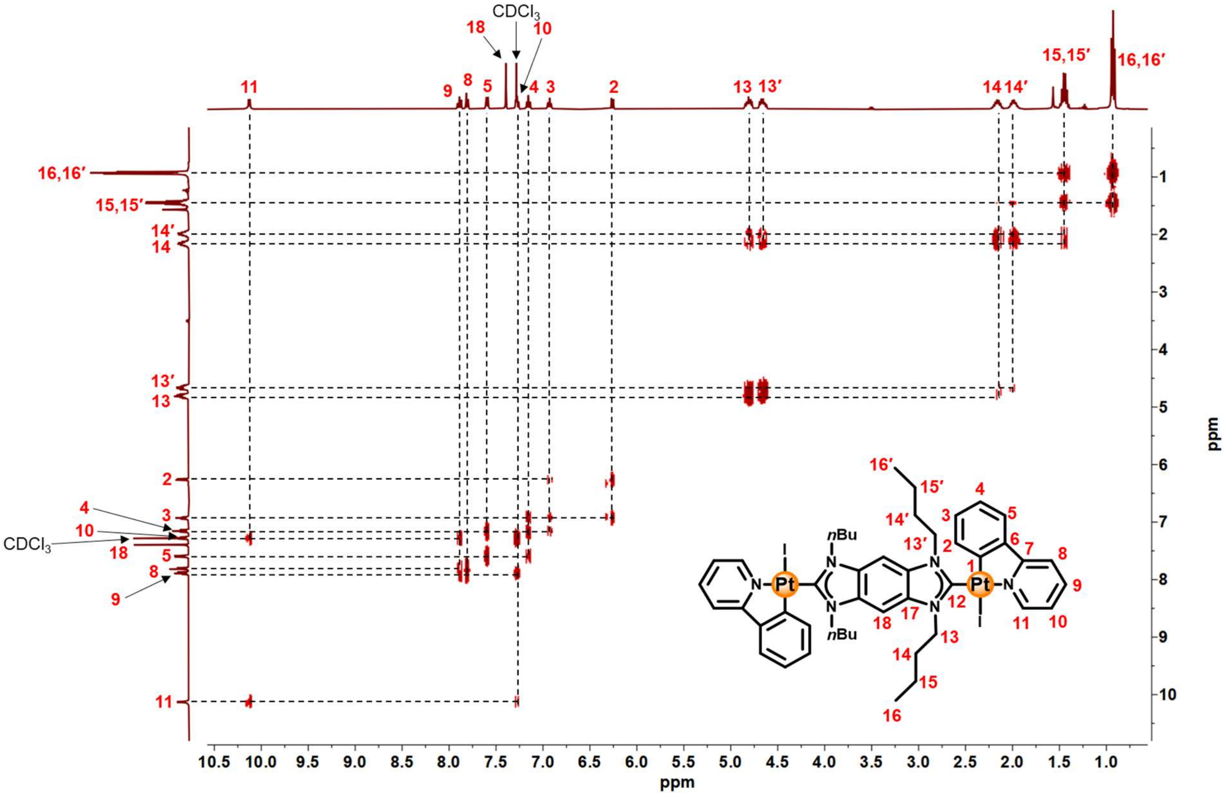

Figure S3. ^{1}H-^{1}H COSY NMR spectrum of *anti*-[Pt$_2$(**1a**)(I)$_2$] (CDCl$_3$, 400 MHz, [2.0 m*M*]).

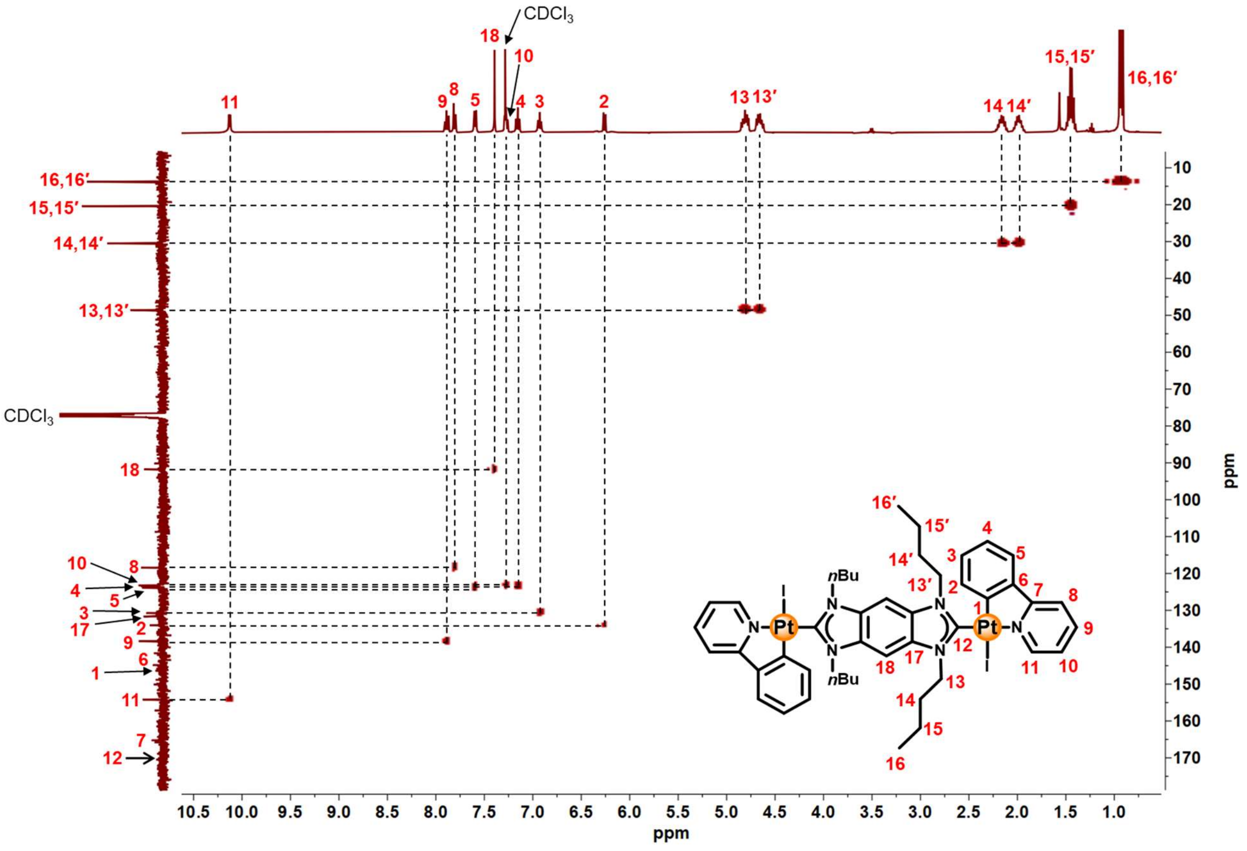

Figure S4. ^{1}H-^{13}C HSQC spectrum of *anti*-[Pt$_2$(**1a**)(I)$_2$] (CDCl$_3$, [2.0 m*M*].

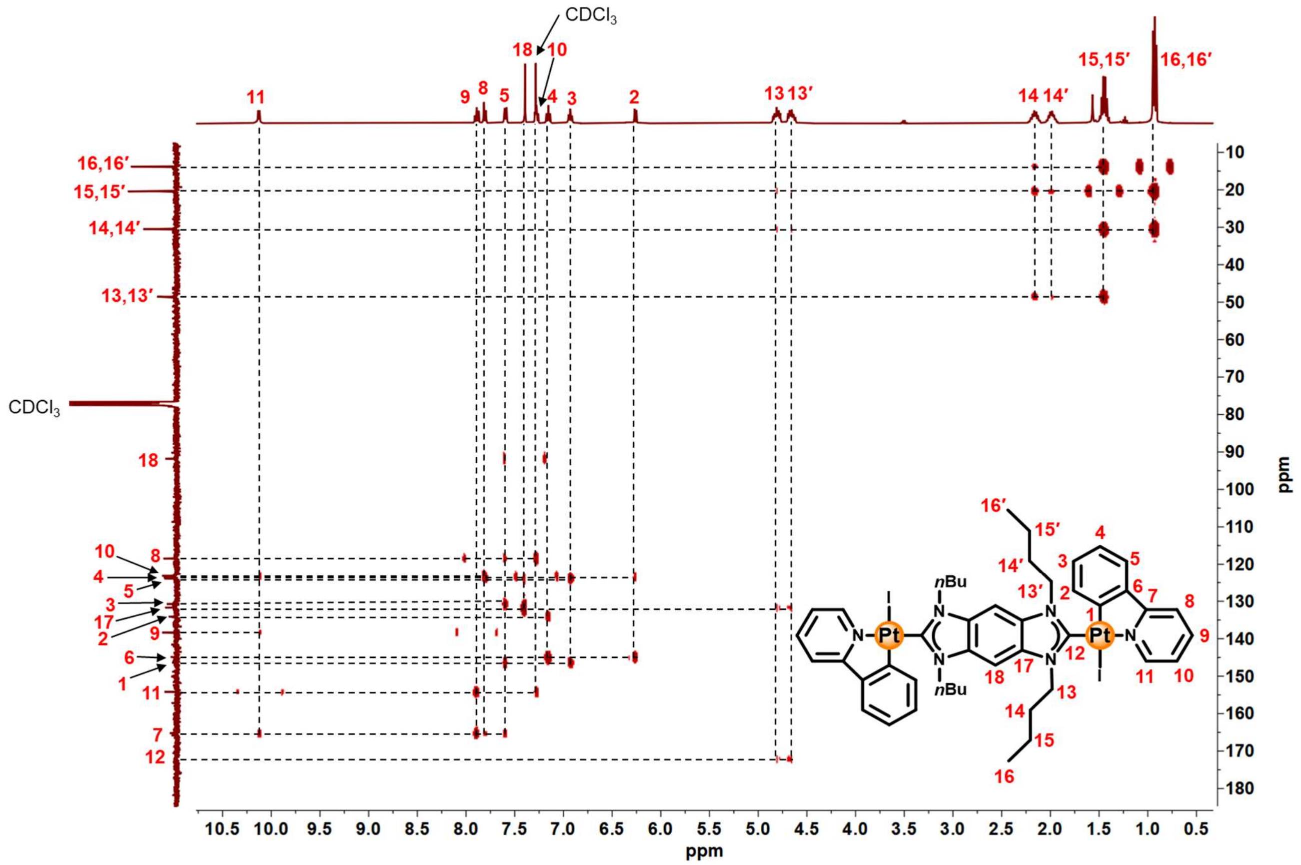

Figure S5. ^{1}H-^{13}C HMBC spectrum of *anti*-[Pt$_2$(**1a**)(I)$_2$] ($CDCl_3$, [2.0 m*M*]).

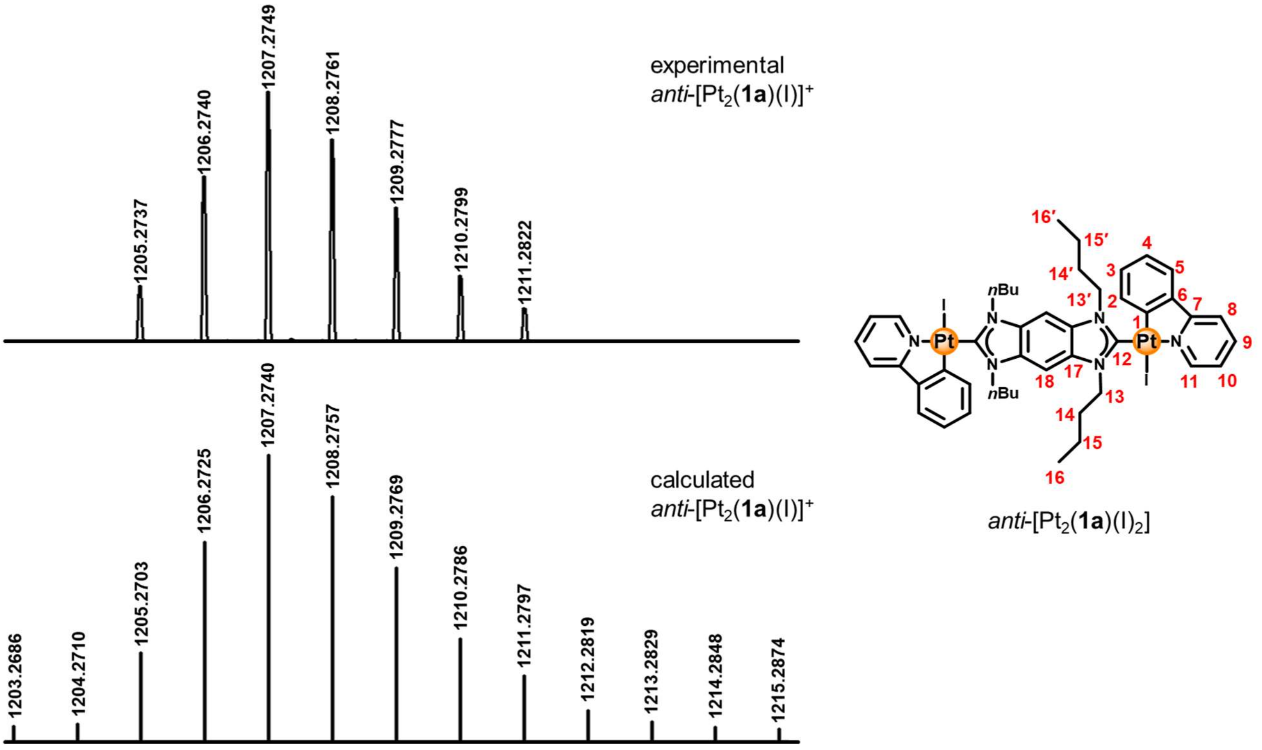

Figure S6. Section of the experimental (top) and calculated (bottom) HRMS (ESI positive ions) spectrum (positive ions) of *anti*-[Pt$_2$(**1a**)(I)$_2$].

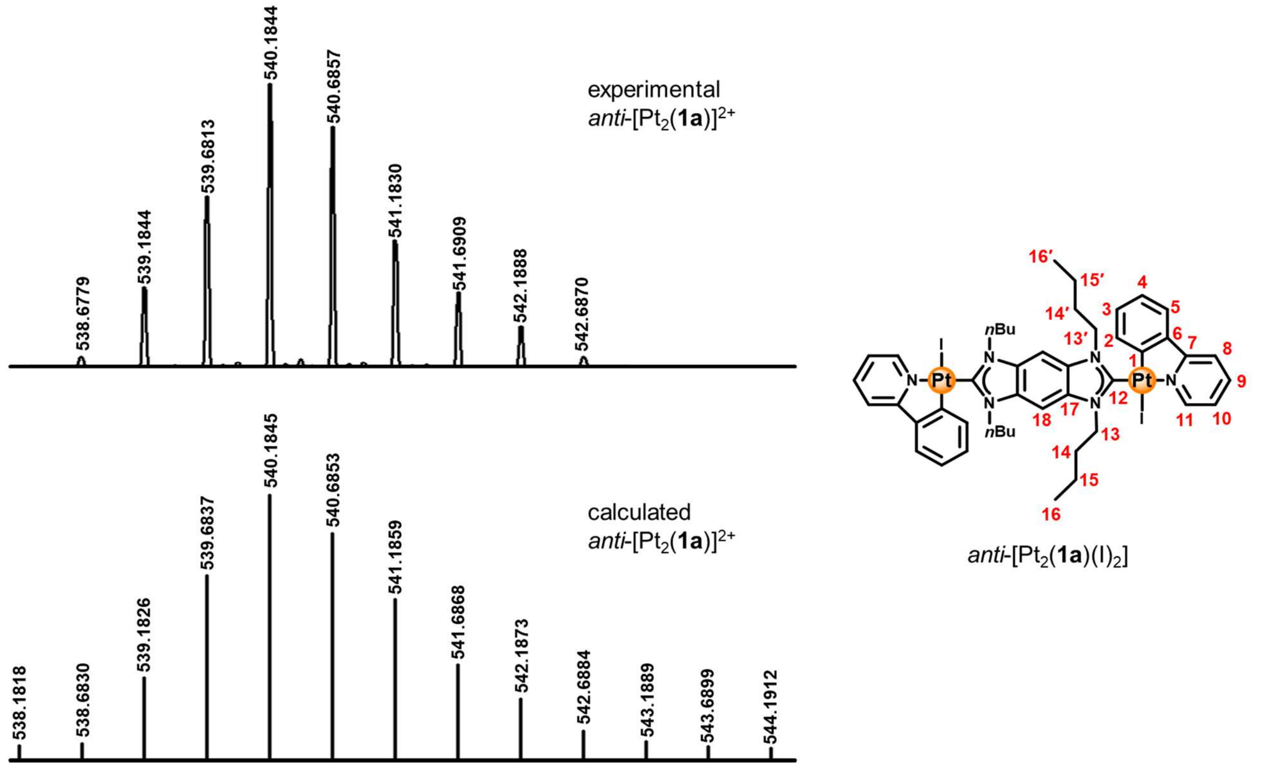

Figure S7. Section of the experimental (top) and calculated (bottom) HRMS (ESI positive ions) spectrum (positive ions) of *anti*-[Pt_2(**1a**)(**I**)$_2$].

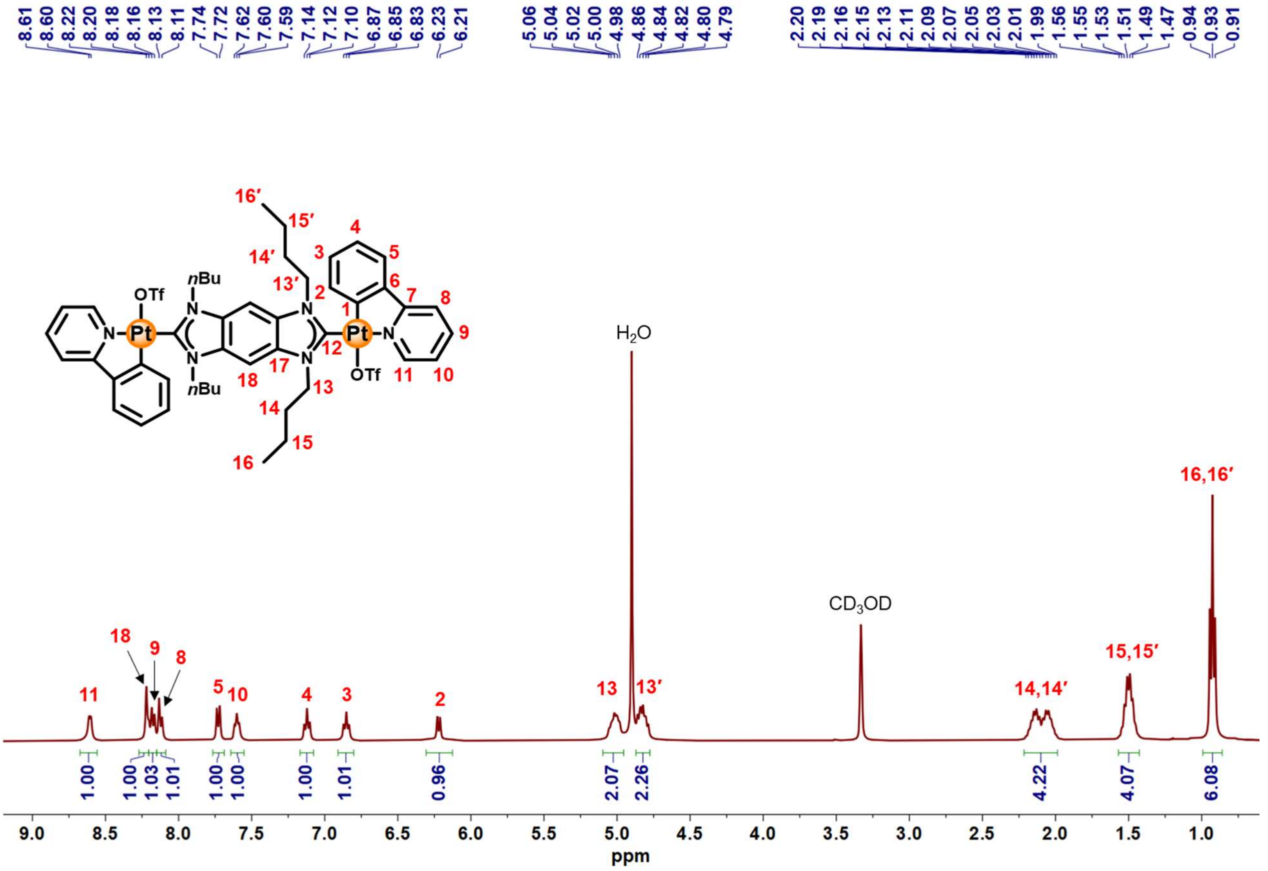

Figure S8. ^{1}H NMR spectrum of *anti*-[Pt_2(**1a**)(OTf)$_2$] (CD_3OD, 400 MHz, [8.0 m*M*]).

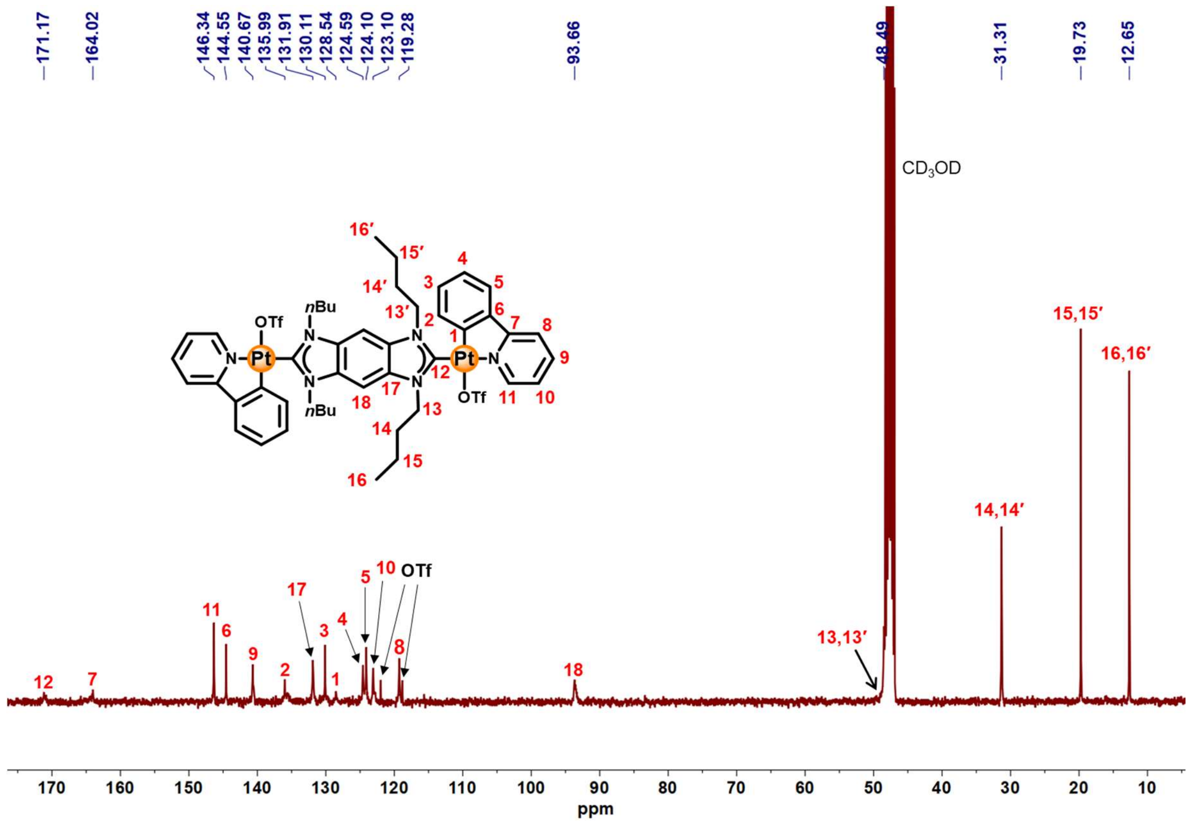

Figure S9. $^{13}C\{^1H\}$ NMR spectrum of *anti*-[Pt_2(**1a**)$(OTf)_2$] (CD_3OD, 100 MHz, [8.0 m*M*]).

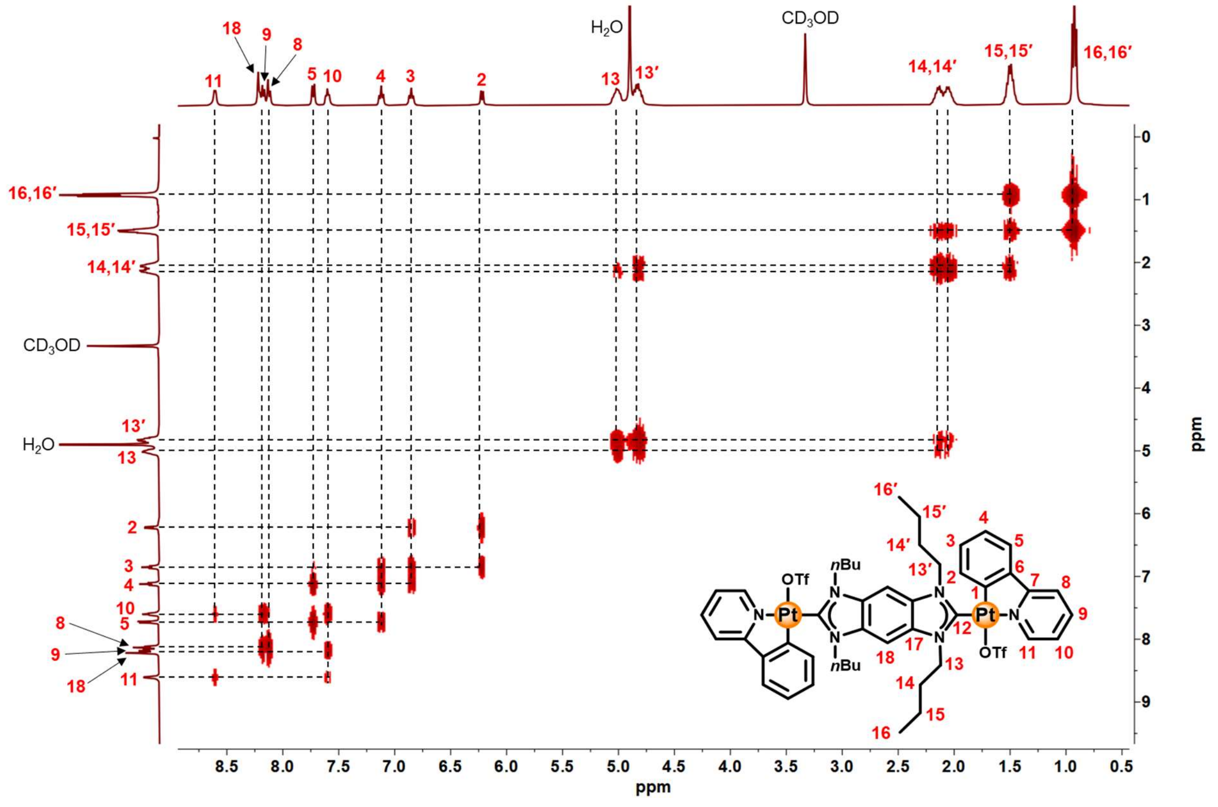

Figure S10. ^1H-^1H COSY NMR spectrum of *anti*-[Pt_2(**1a**)$(OTf)_2$] (CD_3OD, 400 MHz, [8.0 m*M*]).

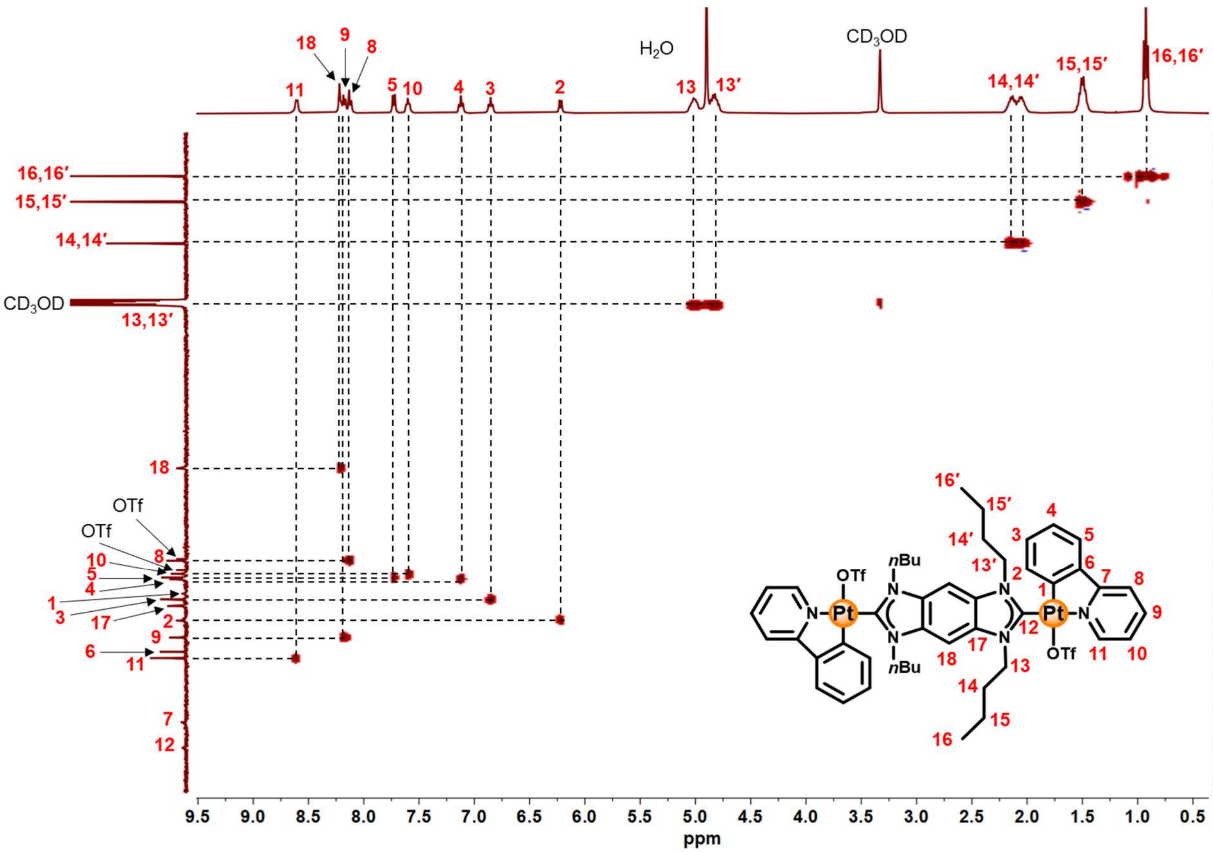

Figure S11. ^{1}H-^{13}C HSQC spectrum of *anti*-[Pt$_2$(**1a**)(OTf)$_2$] (CD$_3$OD, [8.0 m*M*]).

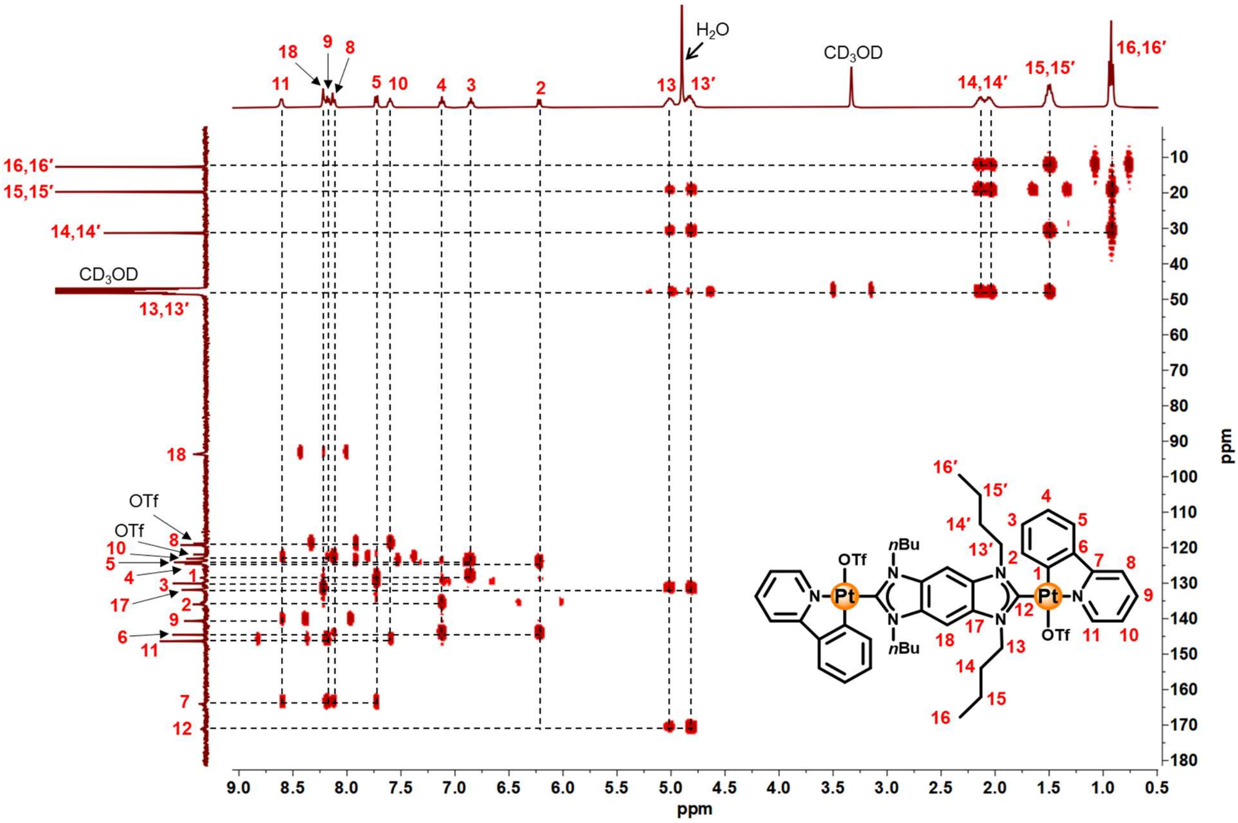

Figure S12. ^{1}H-^{13}C HMBC spectrum of *anti*-[Pt$_2$(**1a**)(OTf)$_2$] (CD$_3$OD, [8.0 m*M*]).

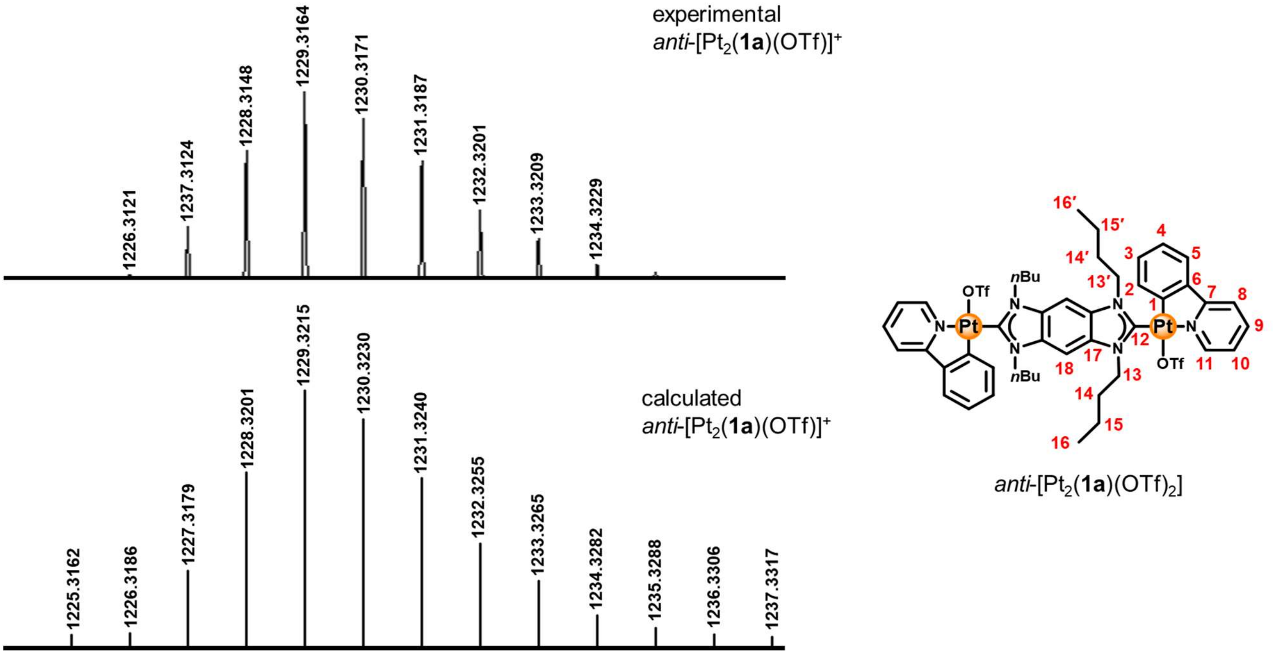

Figure S13. Section of the experimental (top) and calculated (bottom) HRMS (ESI positive ions) spectrum (positive ions) of *anti*-[Pt$_2$(**1a**)(OTf)$_2$].

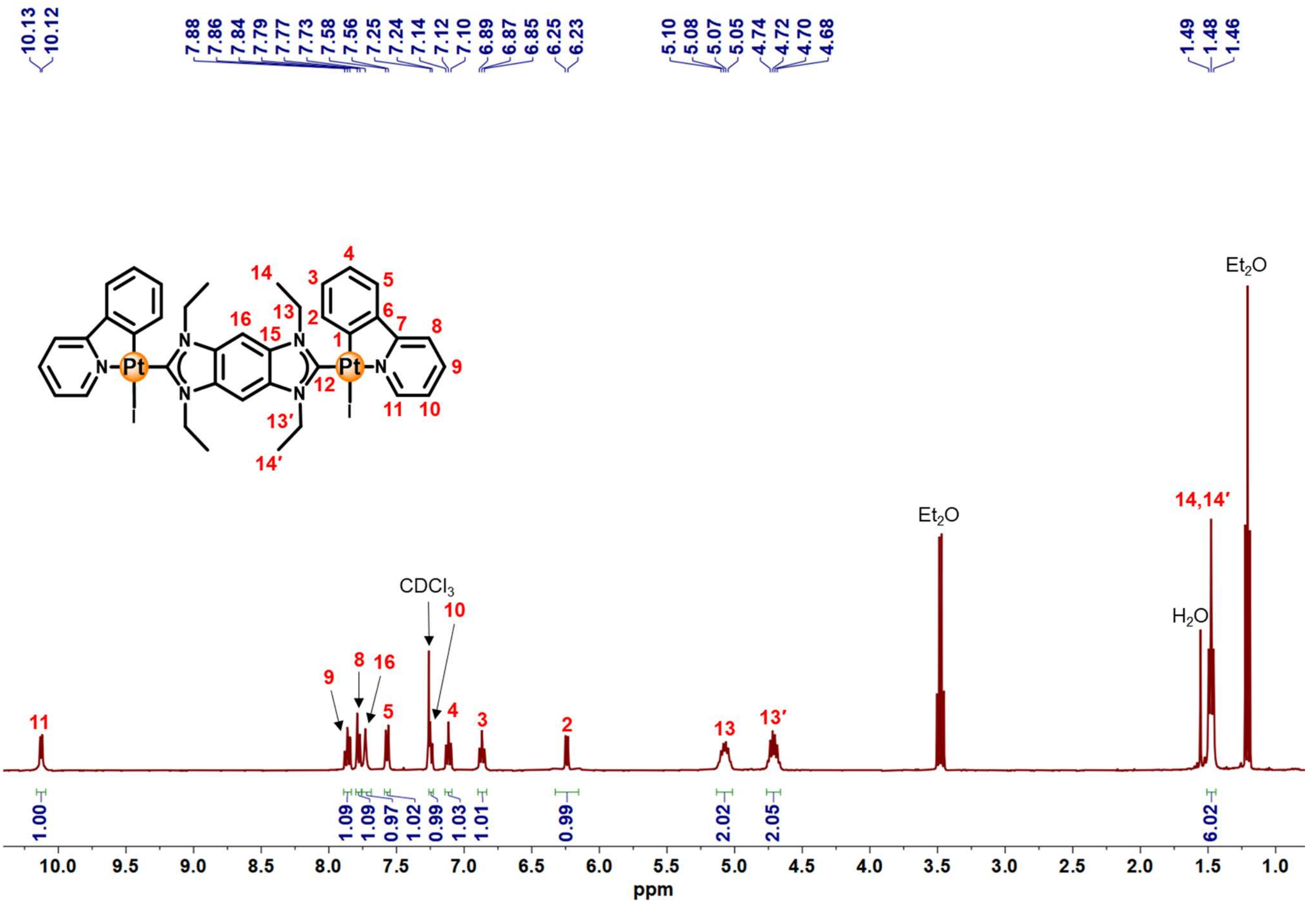

Figure S14. ^{1}H NMR spectrum of *syn*-[Pt$_2$(**1b**)(I)$_2$] ($CDCl_3$, 400 MHz, [3.0 m*M*]).

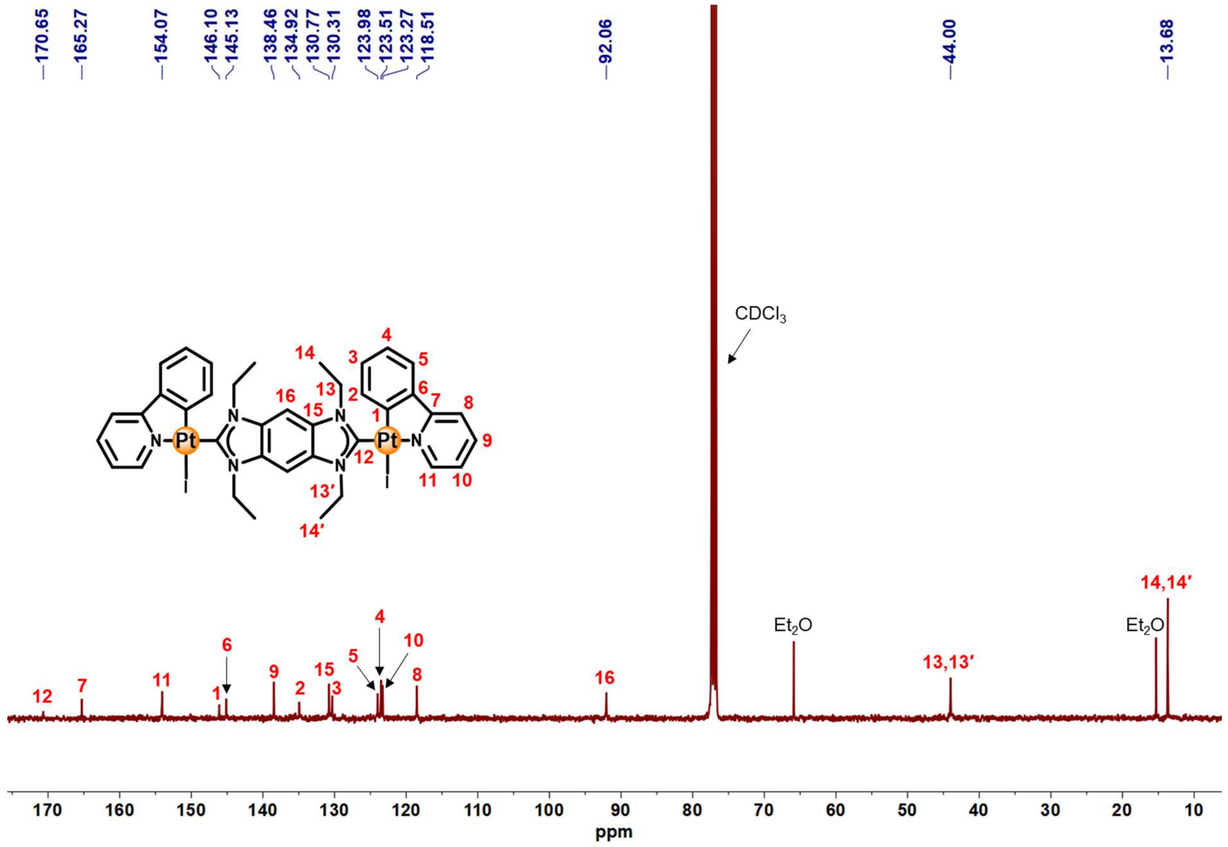

Figure S15. $^{13}C\{^1H\}$ NMR spectrum of *syn*-[Pt_2(**1b**)$(I)_2$] ($CDCl_3$, 100 MHz, [3.0 m*M*]).

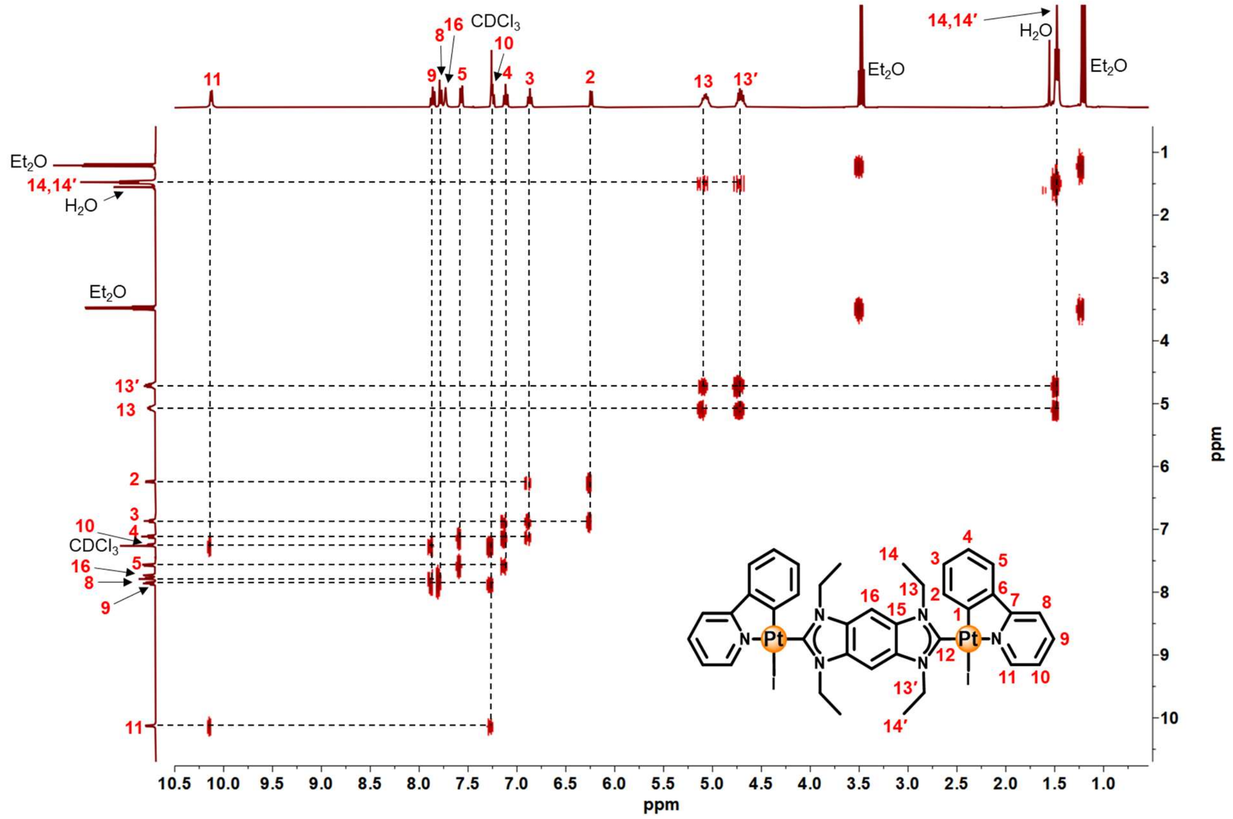

Figure S16. ^1H-^1H COSY NMR spectrum of *syn*-[Pt_2(**1b**)$(I)_2$] ($CDCl_3$, 400 MHz, [3.0 m*M*]).

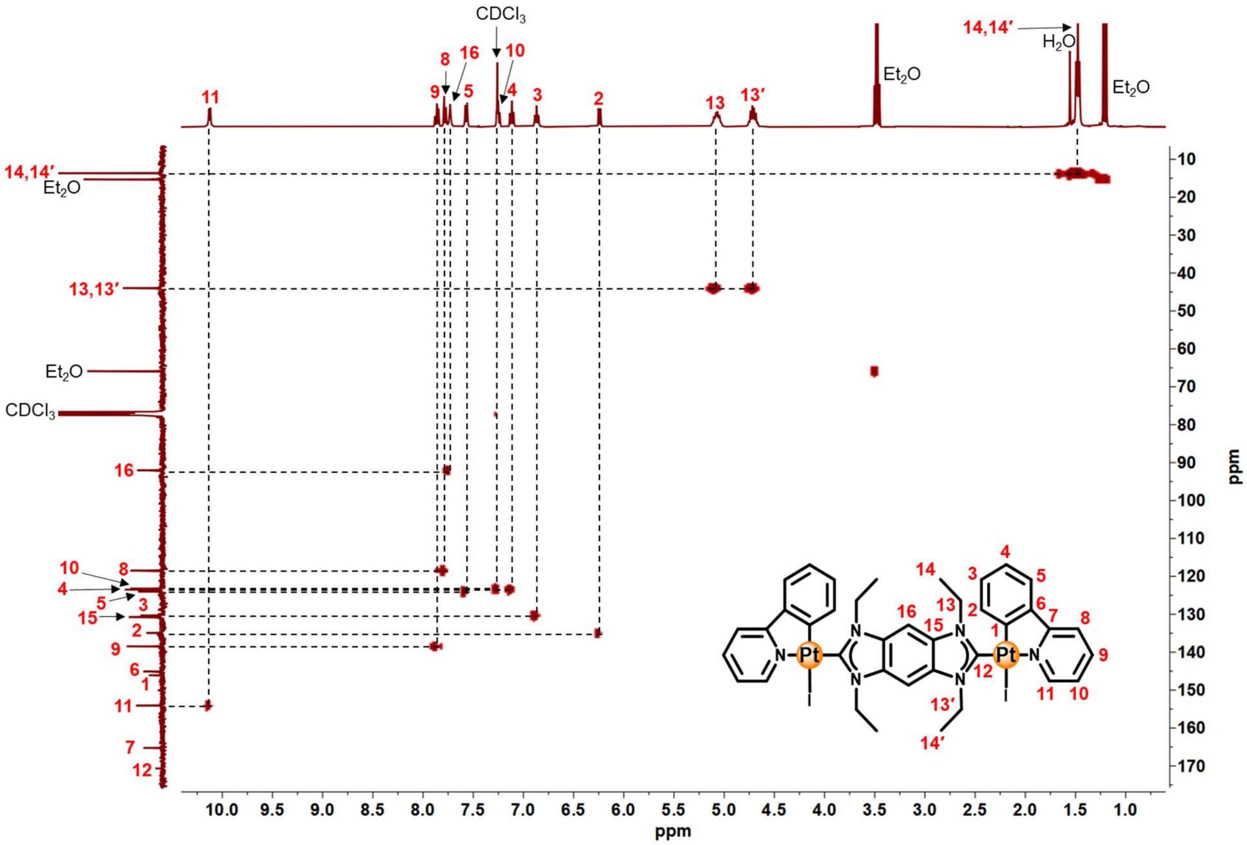

Figure S17. ^{1}H-^{13}C HSQC spectrum of *syn*-[Pt$_2$(**1b**)(I)$_2$] ($CDCl_3$, [3.0 m*M*]).

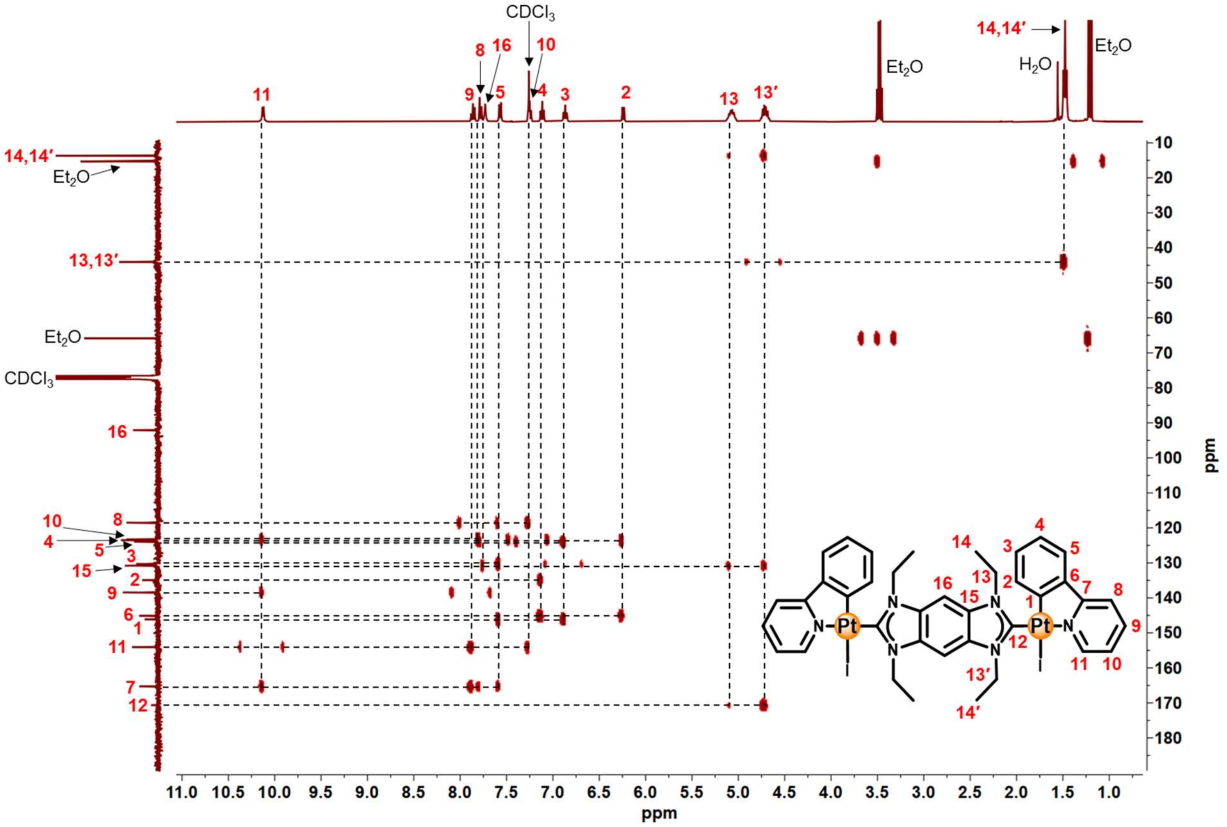

Figure S18. ^{1}H-^{13}C HMBC spectrum of *syn*-[Pt$_2$(**1b**)(I)$_2$] ($CDCl_3$, [3.0 m*M*]).

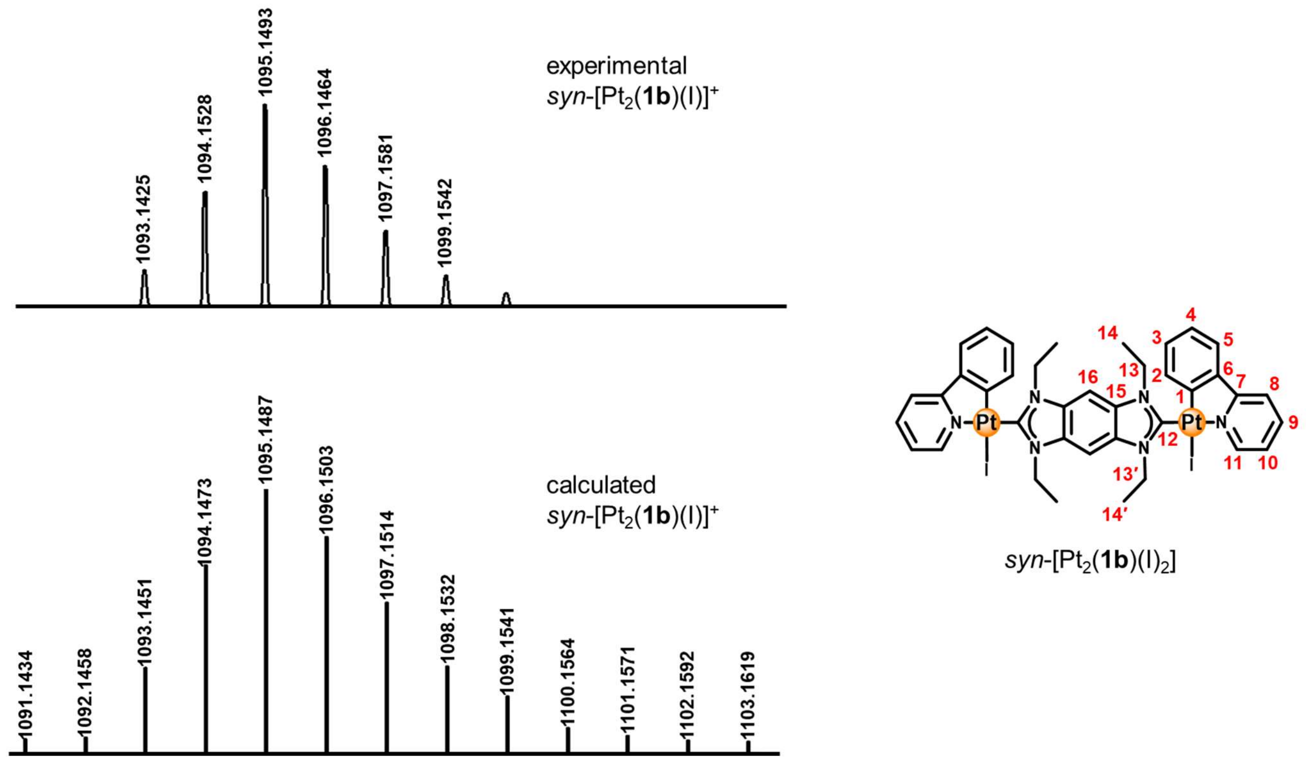

Figure S19. Section of the experimental (top) and calculated (bottom) HRMS (ESI positive ions) spectrum (positive ions) of *syn*-[Pt_2(**1b**)(I)$_2$].

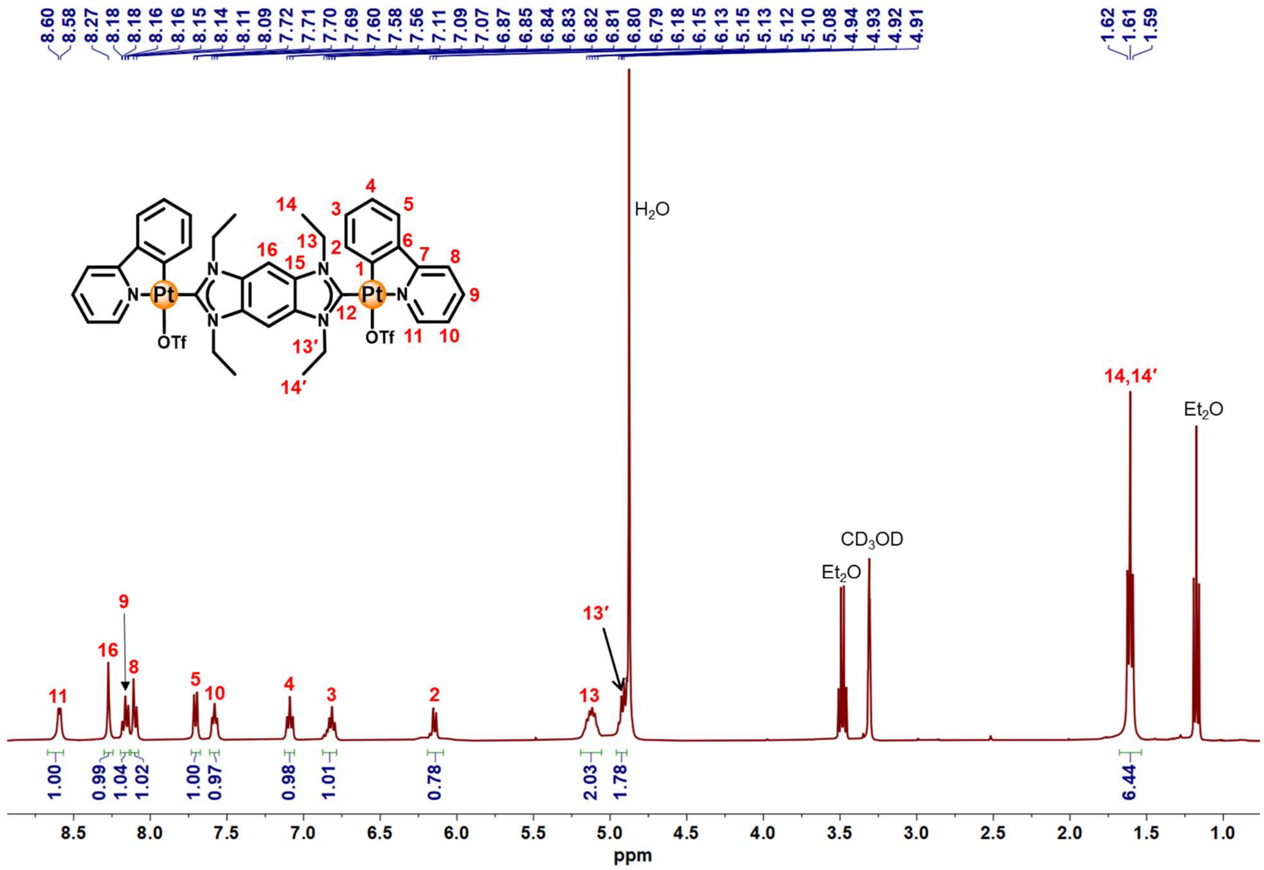

Figure S20. ^{1}H NMR spectrum of *syn*-[Pt_2(**1b**)(OTf)$_2$] (CD_3OD, 400 MHz, [8.0 m*M*]).

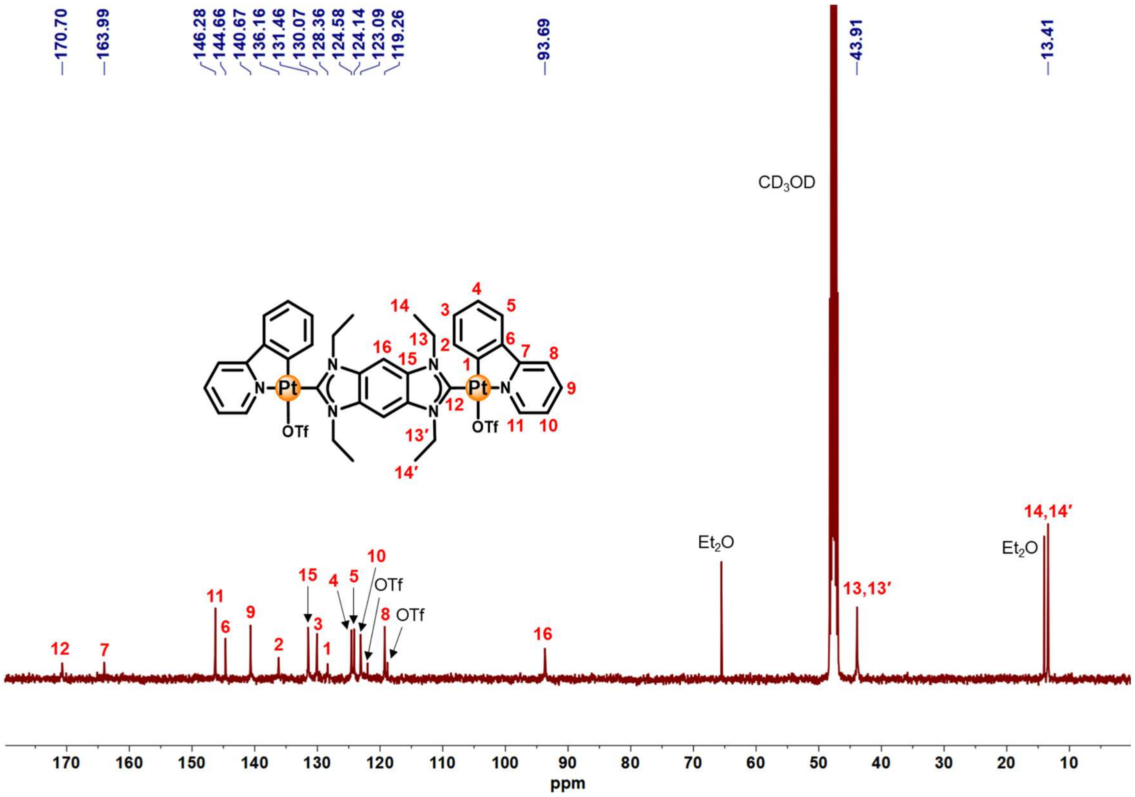

Figure S21. $^{13}C\{^{1}H\}$ NMR spectrum of *syn*-[Pt_2(**1b**)$(OTf)_2$] (CD_3OD, 100 MHz, [8.0 m*M*]).

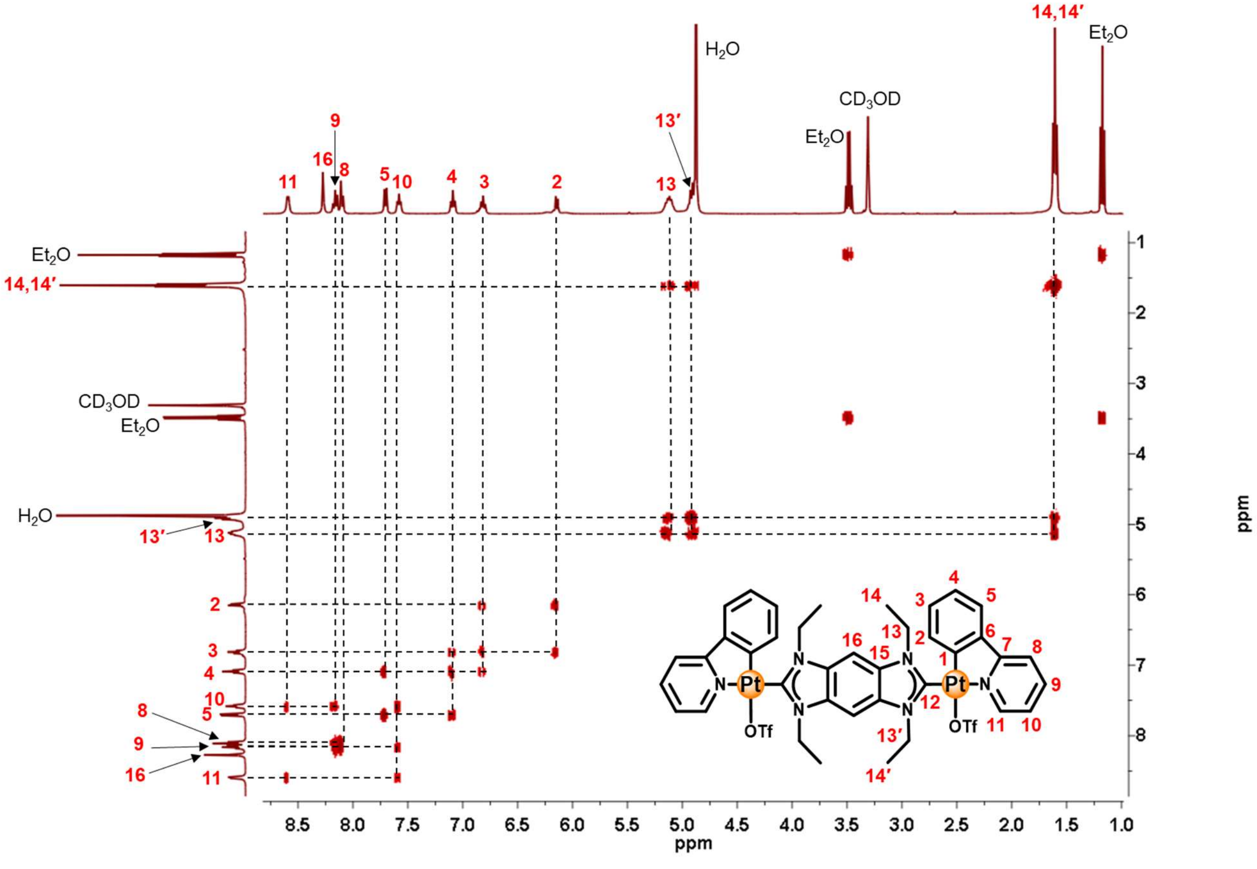

Figure S22. ^{1}H-^{1}H COSY NMR spectrum of *syn*-[Pt_2(**1b**)$(OTf)_2$] (CD_3OD, 400 MHz, [8.0 m*M*]).

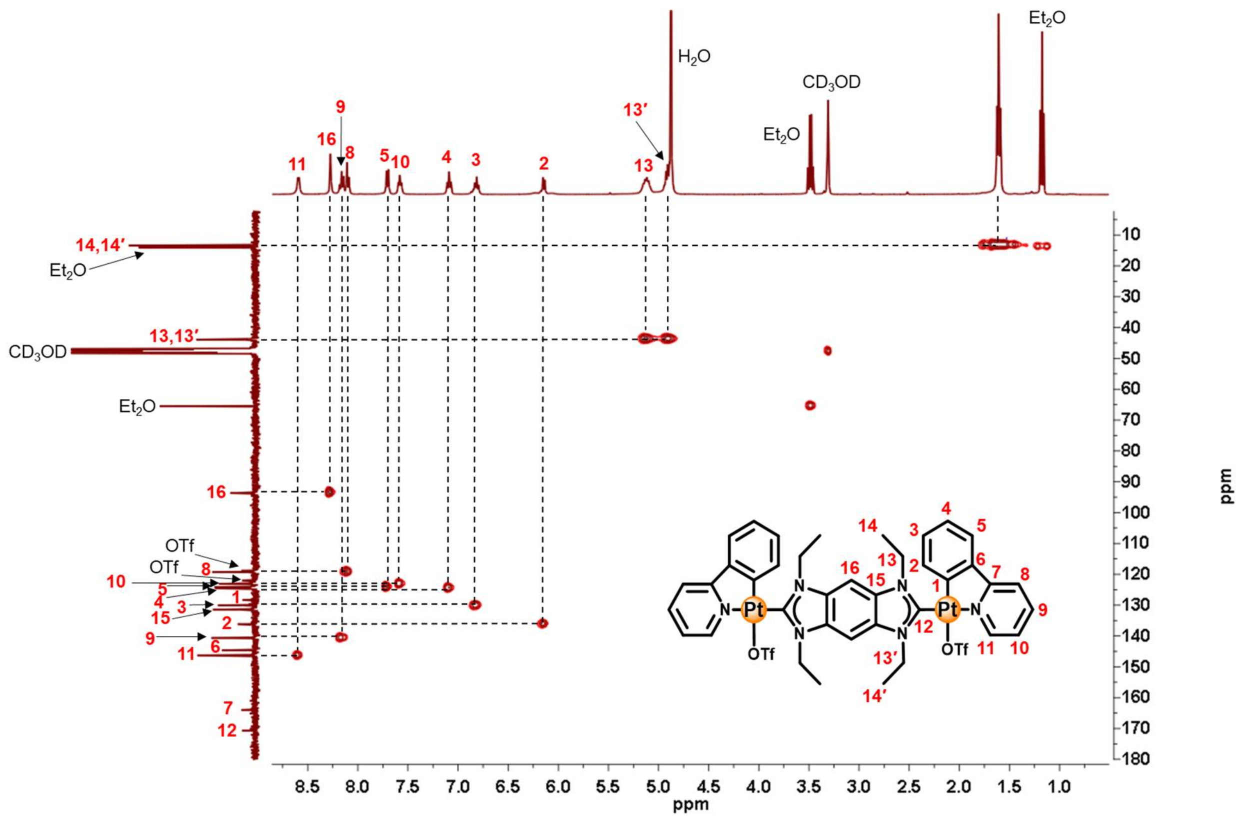

Figure S23. ^{1}H-^{13}C HSQC spectrum of *syn*-[Pt_2(**1b**)$(OTf)_2$] (CD_3OD, [8.0 m*M*]).

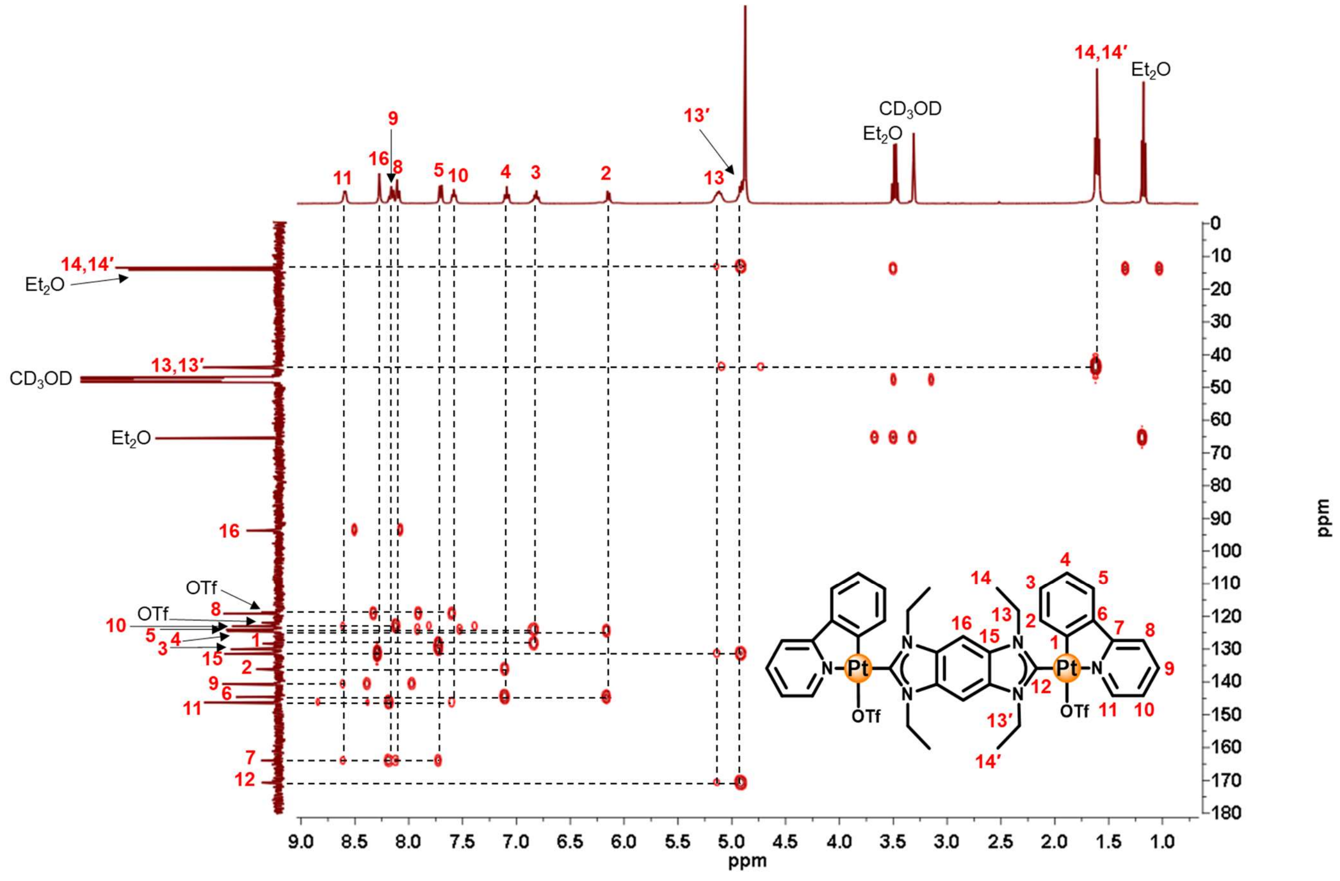

Figure S24. ^{1}H-^{13}C HMBC spectrum of *syn*-[Pt_2(**1b**)$(OTf)_2$] (CD_3OD, [8.0 m*M*]).

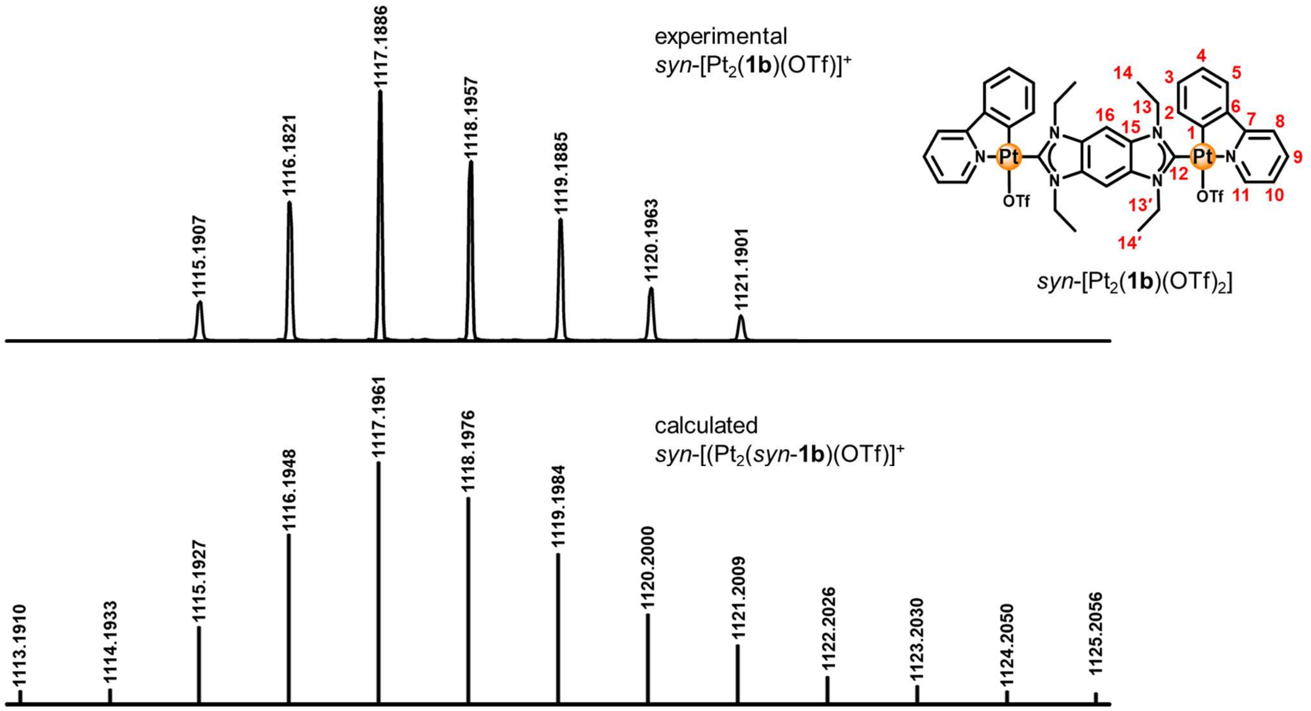

Figure S25. Section of the experimental (top) and calculated (bottom) HRMS (ESI positive ions) spectrum (positive ions) of *syn*-[Pt$_2$(**1b**)(OTf)$_2$].

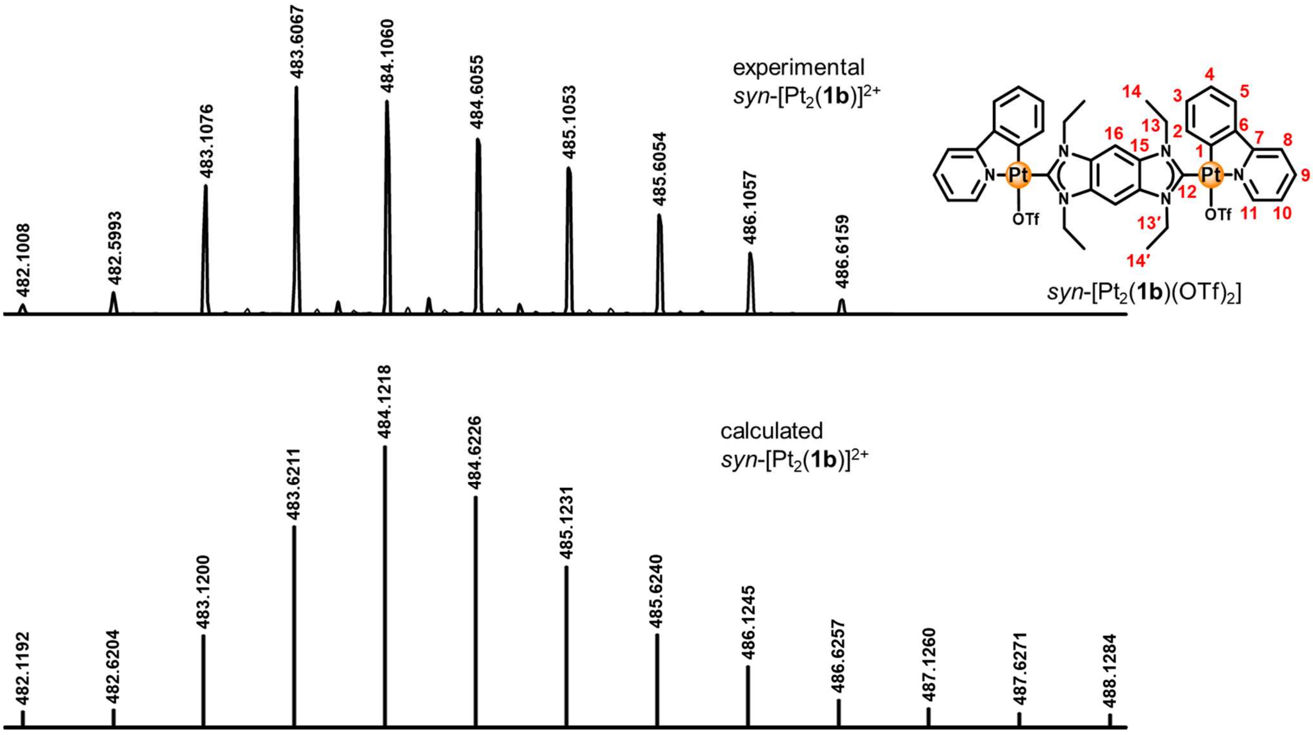

Figure S26. Section of the experimental (top) and calculated (bottom) HRMS (ESI positive ions) spectrum (positive ions) of *syn*-[Pt$_2$(**1b**)(OTf)$_2$].

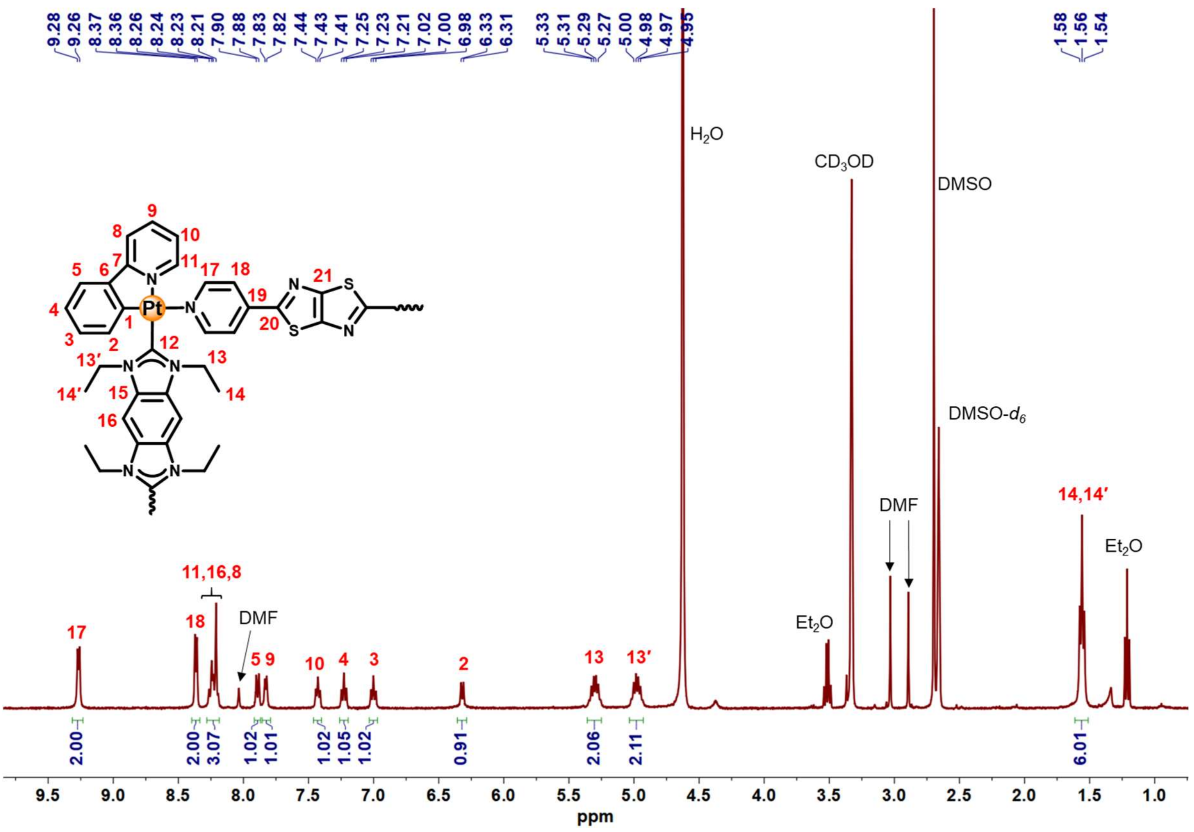

Figure S27. ^{1}H NMR spectrum of [**2b-BRs**](OTf)$_{12}$ (CD$_3$OD/DMSO-*d*$_6$, v:v = 4:1, 400 MHz, [2.0 m*M*]).

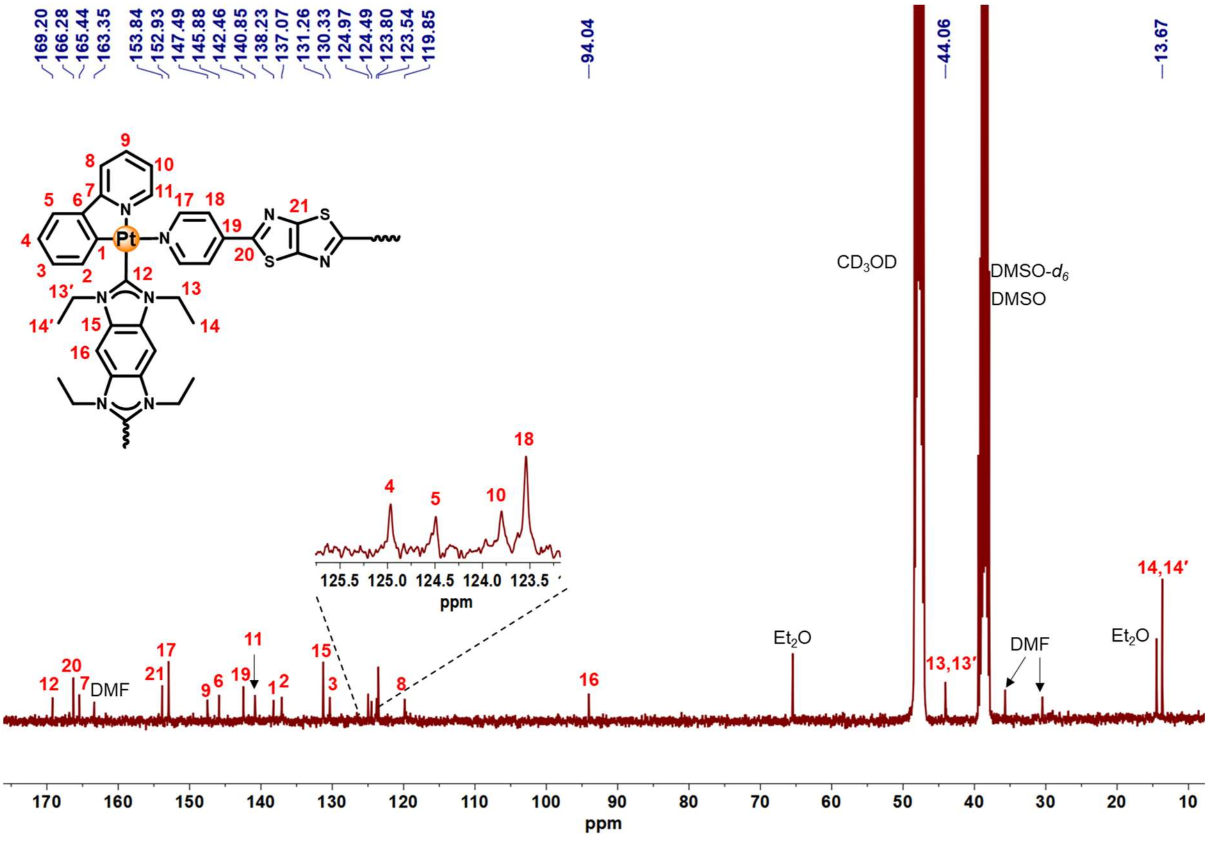

Figure S28. ^{13}C{^{1}H} NMR spectrum of [**2b-BRs**](OTf)$_{12}$ (CD$_3$OD/DMSO-*d*$_6$, v:v = 4:1, 100 MHz, [2.0 m*M*]).

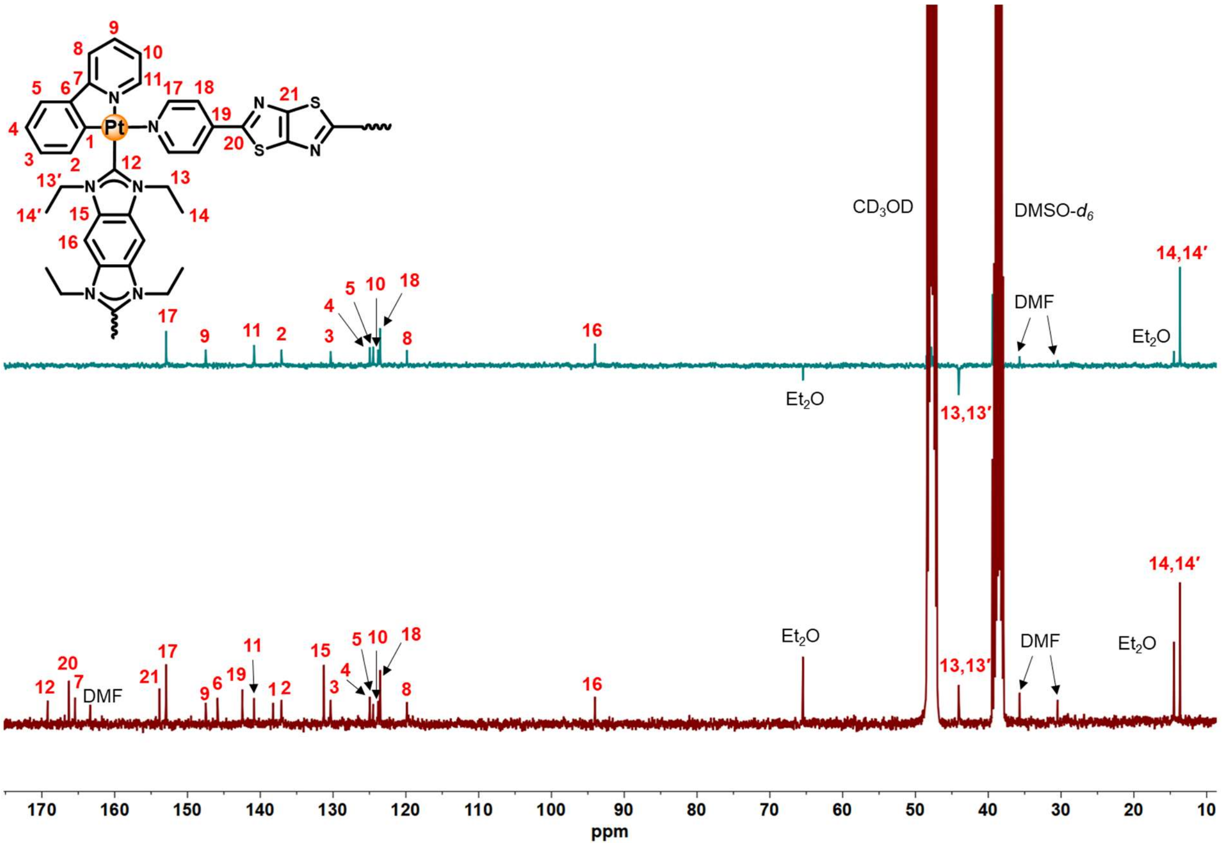

Figure S29. DEPT-135 (top) and $^{13}C\{^1H\}$ NMR spectra (bottom) of [**2b-BRs**](OTf)$_{12}$ (CD_3OD/DMSO-d_6, v:v = 4:1, 100 MHz, [2.0 m*M*]).

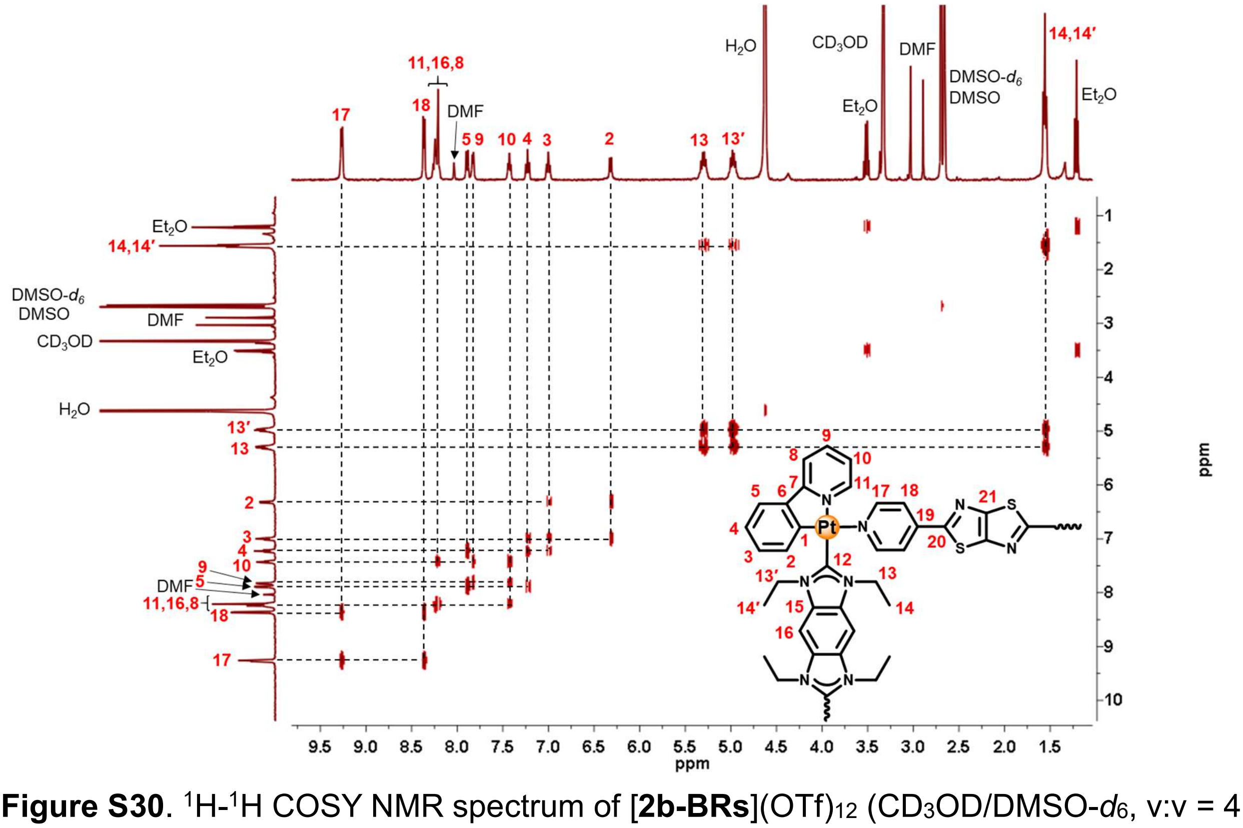

Figure S30. ^1H-^1H COSY NMR spectrum of [**2b-BRs**](OTf)$_{12}$ (CD_3OD/DMSO-d_6, v:v = 4:1, 400 MHz, [2.0 m*M*]).

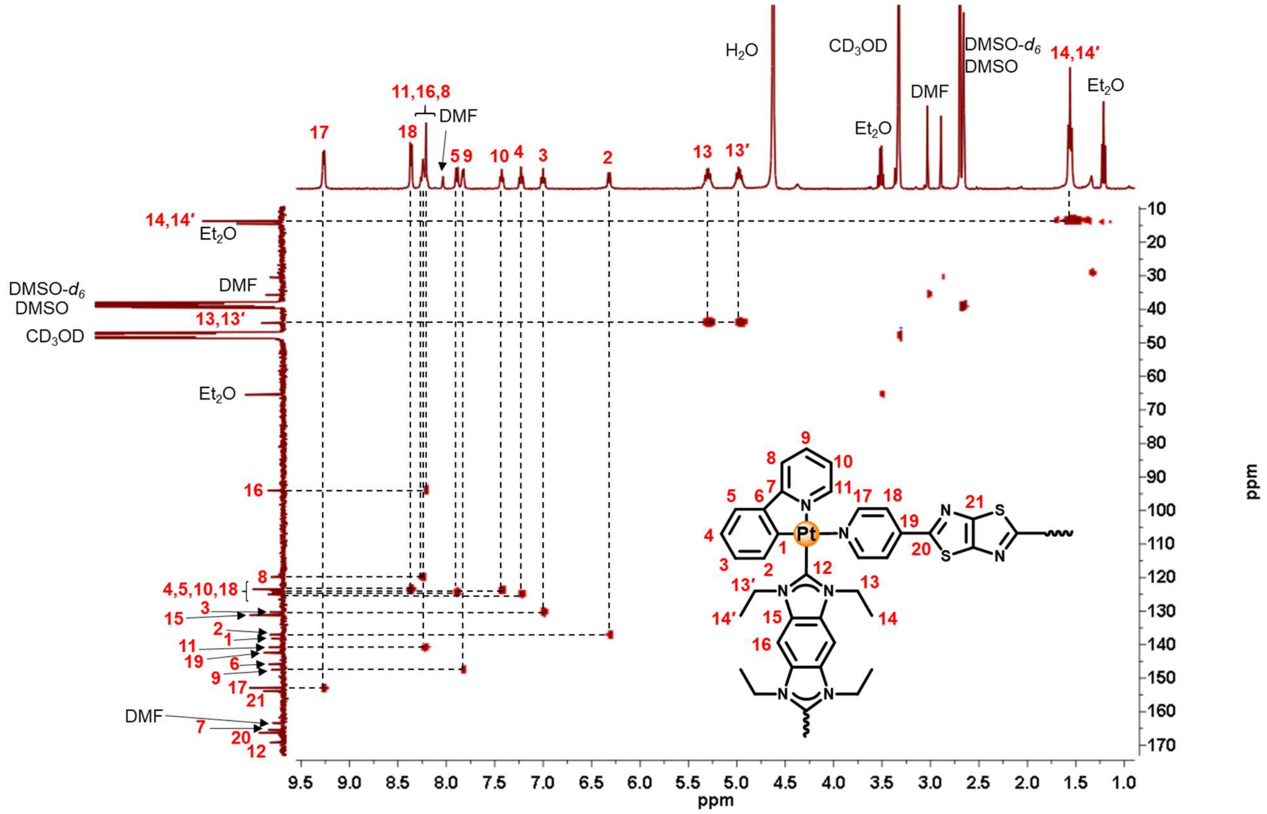

Figure S31. ^{1}H-^{13}C HSQC spectrum of [**2b-BRs**](OTf)$_{12}$ (CD_3OD/DMSO-d_6, v:v = 4:1, [2.0 m*M*]).

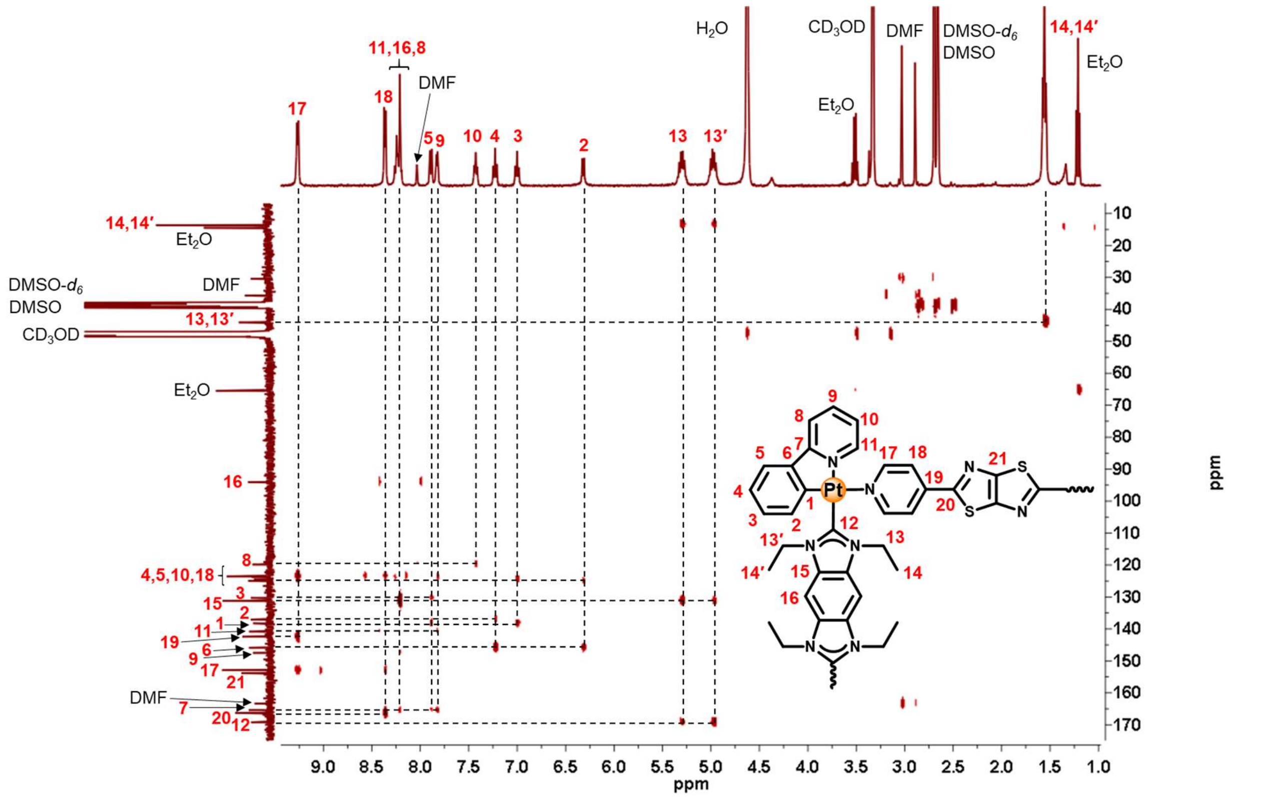

Figure S32. ^{1}H-^{13}C HMBC spectrum of [**2b-BRs**](OTf)$_{12}$ (CD_3OD/DMSO-d_6, v:v = 4:1, [2.0 m*M*]).

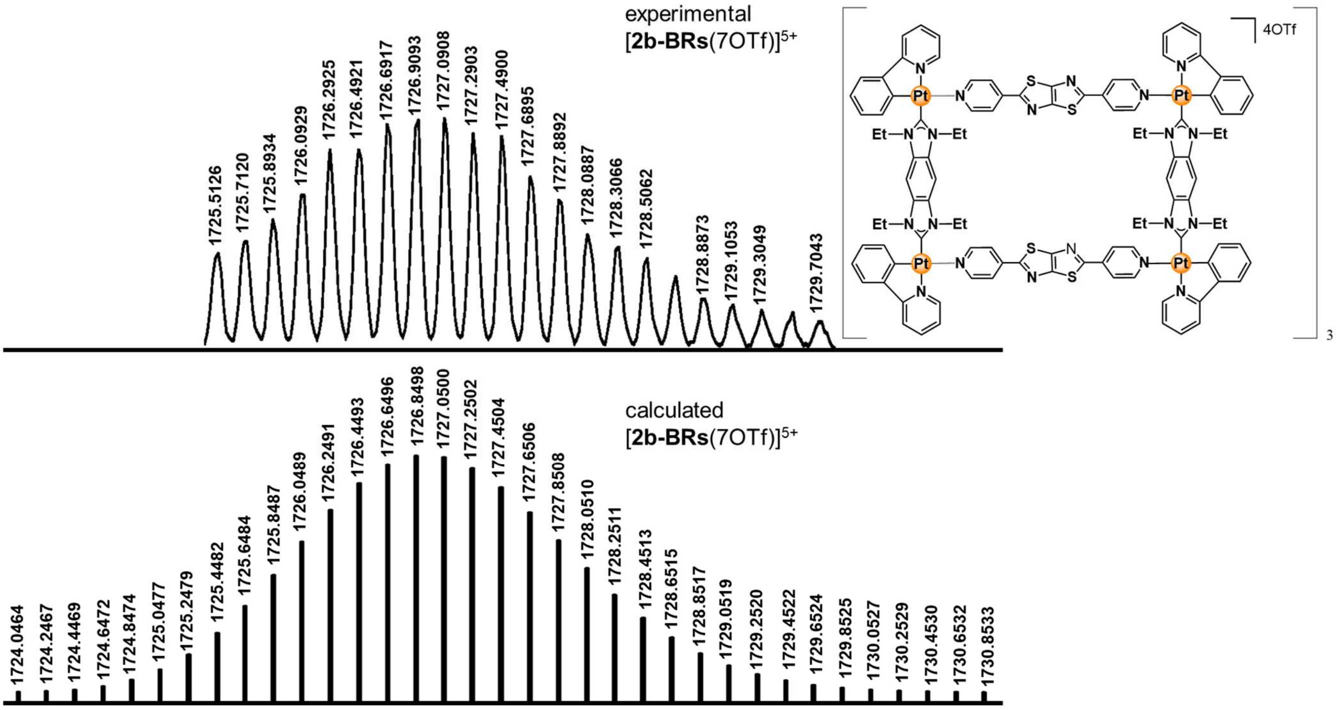

Figure S33. Section of the experimental (top) and calculated (bottom) HRMS (ESI positive ions) spectrum (positive ions) of [**2b-BRs**](OTf)$_{12}$.

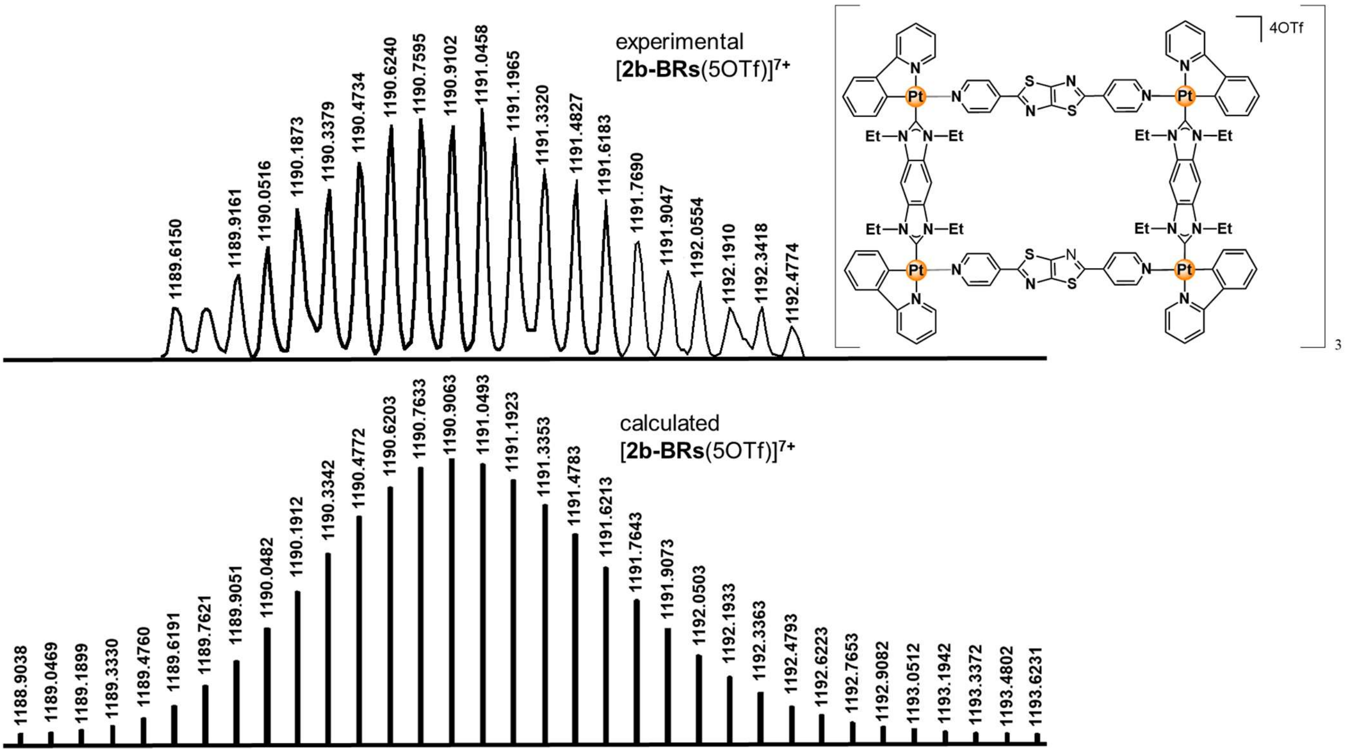

Figure S34. Section of the experimental (top) and calculated (bottom) HRMS (ESI positive ions) spectrum (positive ions) of [**2b-BRs**](OTf)$_{12}$.

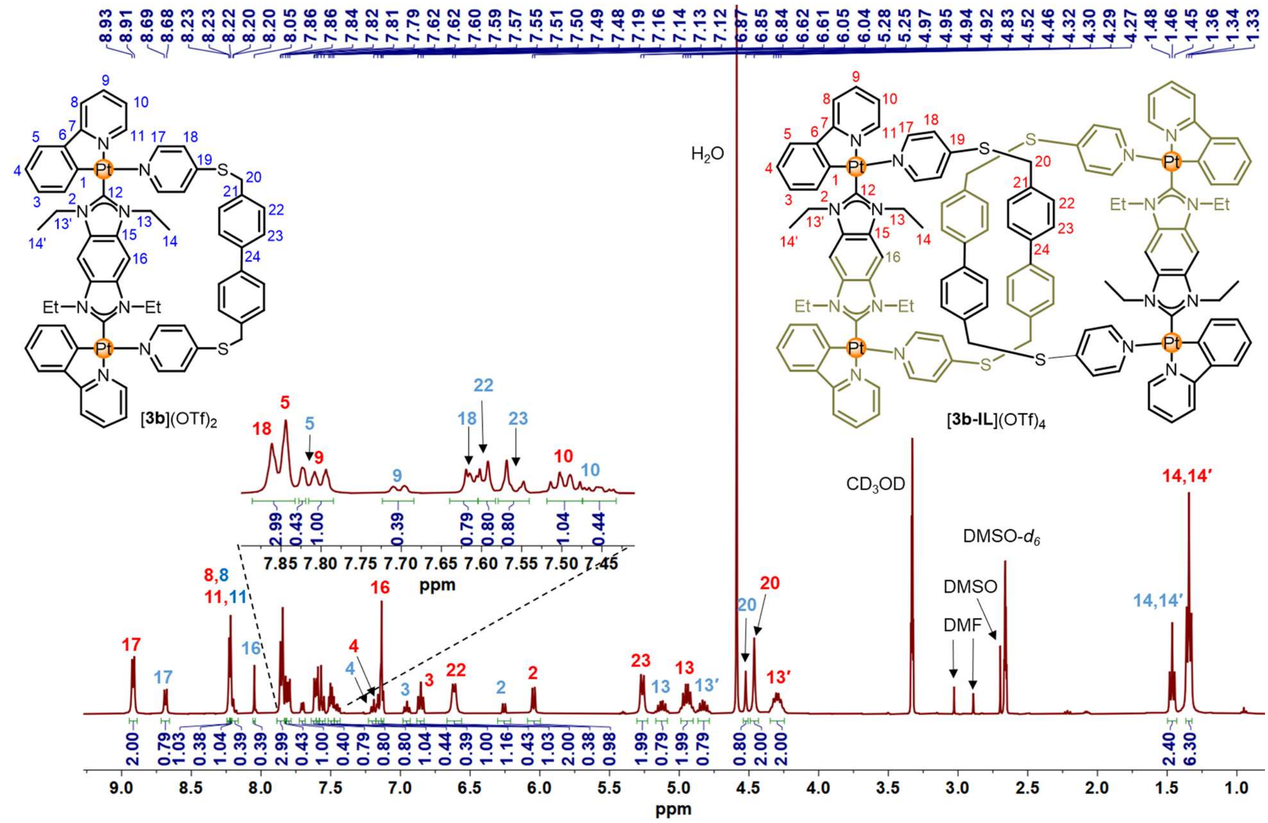

Figure S35. ^{1}H NMR spectrum of the mixture [**3b**](OTf)$_2$ (blue) and [**3b-IL**](OTf)$_4$ (red) (CD$_3$OD/DMSO-d_6 v:v = 4:1, 400 MHz, [4.0 m*M*]. The equilibrium constant (*K*) has been calculated to be approximately 1094.

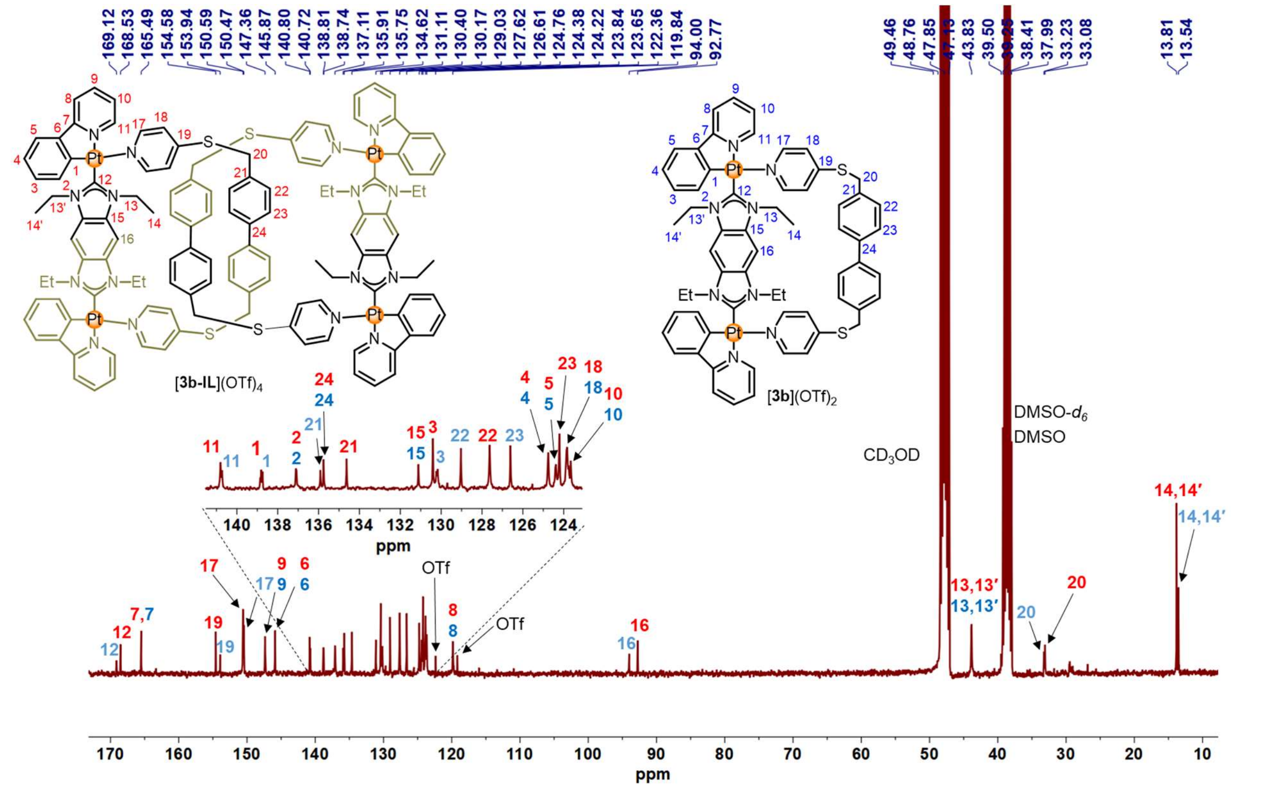

Figure S36. $^{13}C\{^{1}H\}$ NMR spectrum of the mixture [**3b**](OTf)$_2$ (blue) and [**3b-IL**](OTf)$_4$ (red) (CD$_3$OD/DMSO-d_6 v:v = 4:1, 100 MHz, [4.0 m*M*].

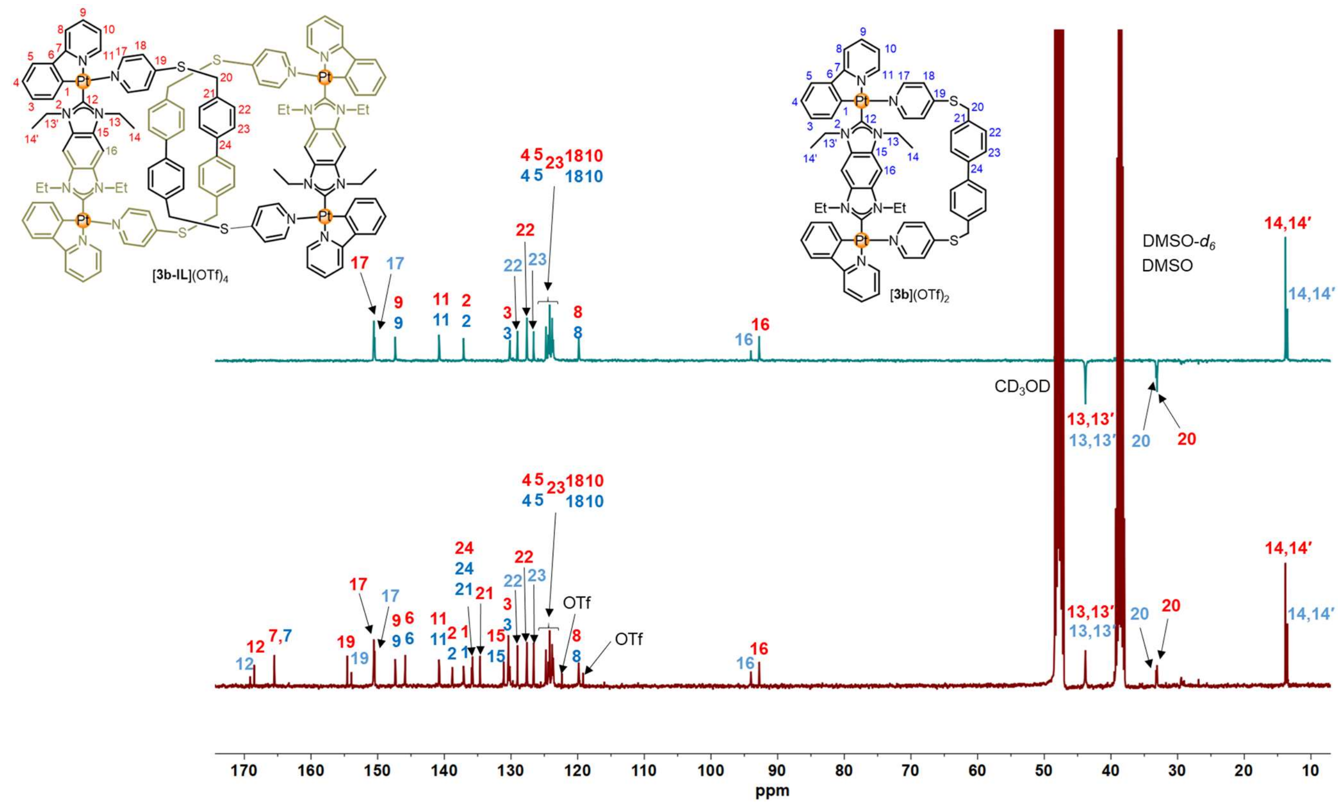

Figure S37. DEPT-135 (top) and $^{13}C\{^1H\}$ NMR spectra (bottom) of the mixture [**3b**]$(OTf)_2$ (blue) and [**3b-IL**]$(OTf)_4$ (red) (CD_3OD/DMSO-d_6 v:v = 4:1, 100 MHz, [4.0 m*M*].

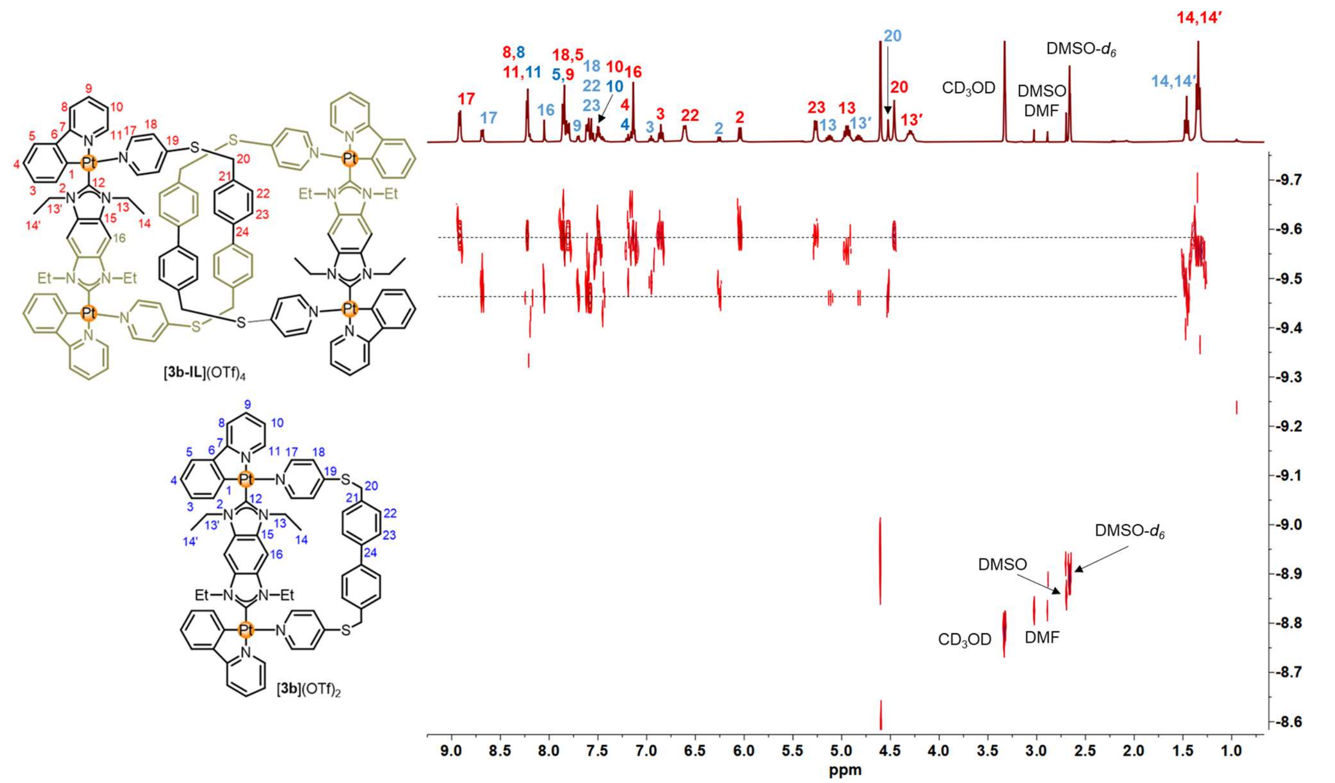

Figure S38. ^1H DOSY spectrum of of the mixture [**3b**]$(OTf)_2$ (blue) and [**3b-IL**]$(OTf)_4$ (red) (CD_3OD/DMSO-d_6 v:v = 4:1, 400 MHz, [4.0 m*M*].

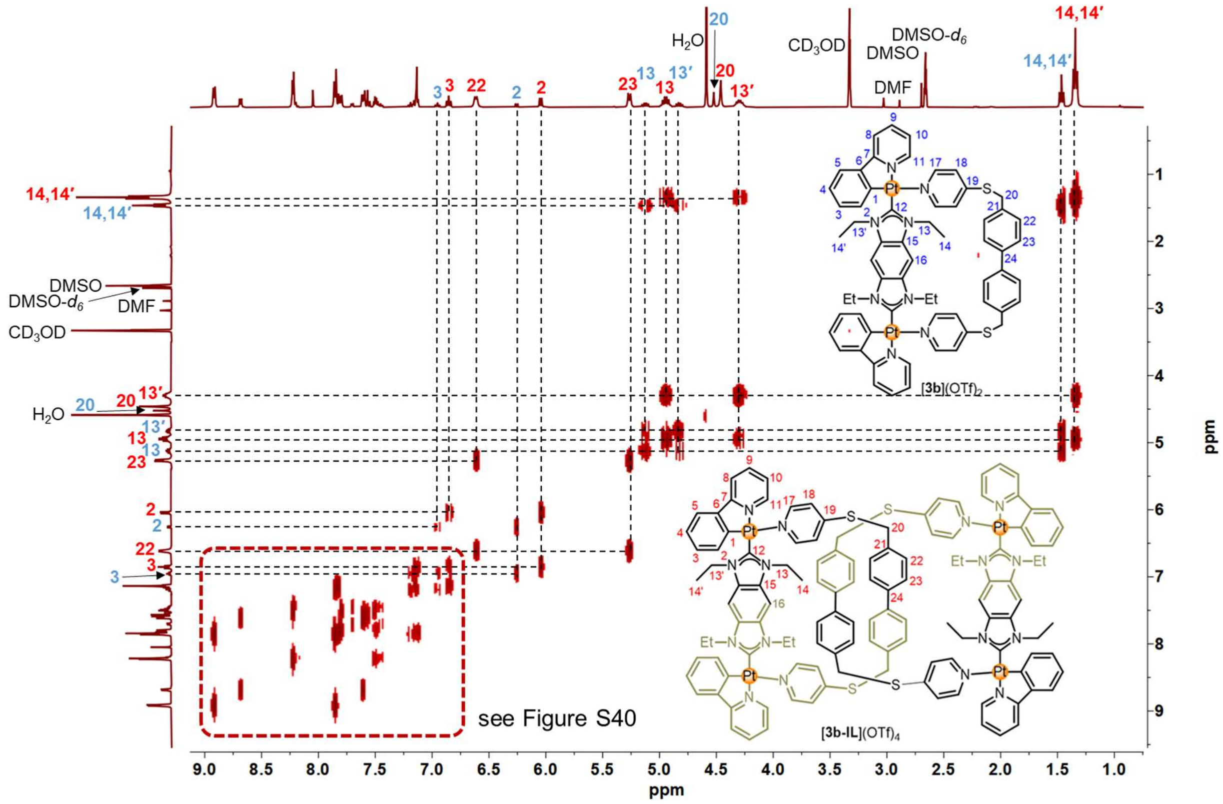

Figure S39. ^1H-^1H COSY NMR spectrum of the mixture [**3b**](OTf)$_2$ (blue) and [**3b-IL**](OTf)$_4$ (red) (CD$_3$OD/DMSO-*d*$_6$ v:v = 4:1, 400 MHz, [4.0 m*M*].

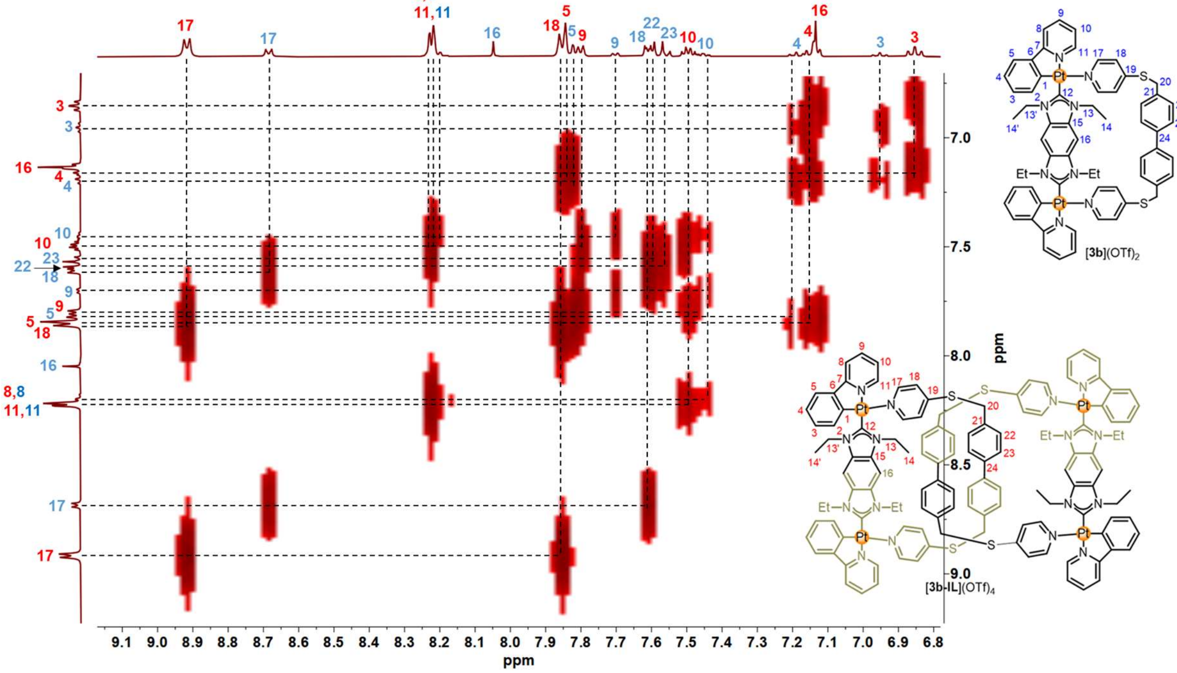

Figure S40. Partial ^1H-^1H COSY NMR spectrum of the mixture [**3b**](OTf)$_2$ (blue) and [**3b-IL**](OTf)$_4$ (red) (CD$_3$OD/DMSO-*d*$_6$ v:v = 4:1, 400 MHz, [4.0 m*M*]. The ^1H-^1H COSY spectrum failed to reveal the correlation between H8 and H9. However, the assignment of these protons was clearly established using the ^1H-^{13}C HSQC and ^1H-^{13}C HMBC spectra (Figure S42 and S44)

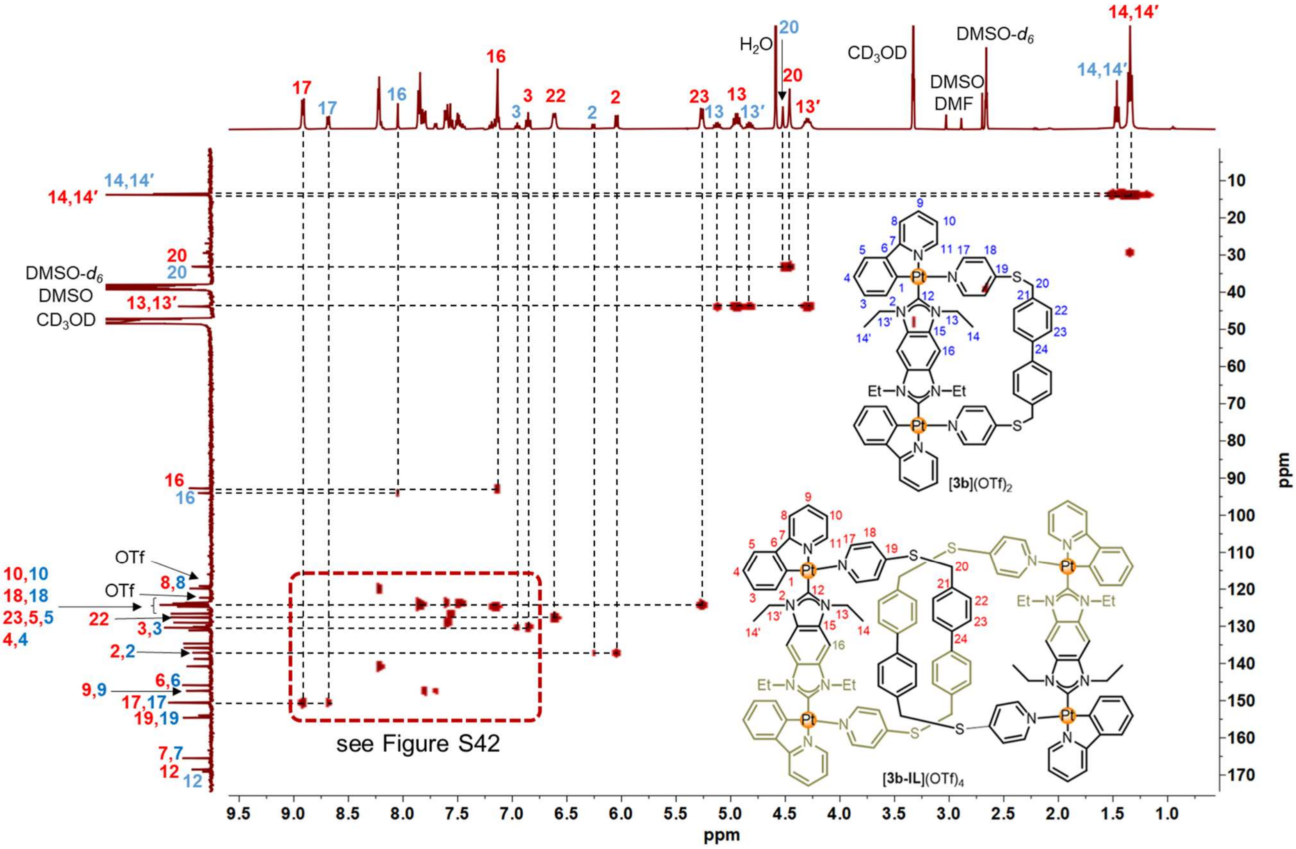

Figure S41. ^1H-^{13}C HSQC spectrum of the mixture [**3b**](OTf)$_2$ (blue) and [**3b-IL**](OTf)$_4$ (red) (CD$_3$OD/DMSO-d_6 v:v = 4:1, [4.0 m*M*].

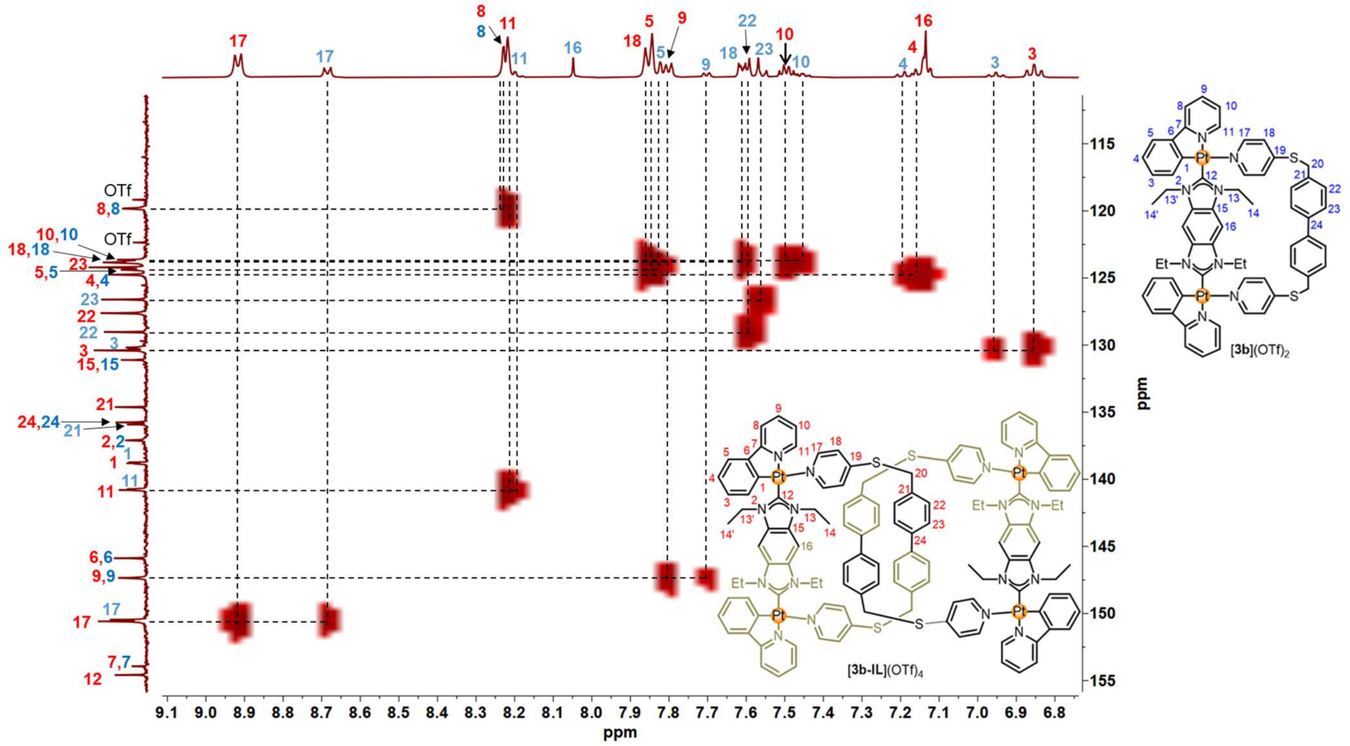

Figure S42. Partial ^1H-^{13}C HSQC spectrum of the mixture [**3b**](OTf)$_2$ (blue) and [**3b-IL**](OTf)$_4$ (red) (CD$_3$OD/DMSO-d_6 v:v = 4:1, [4.0 m*M*].

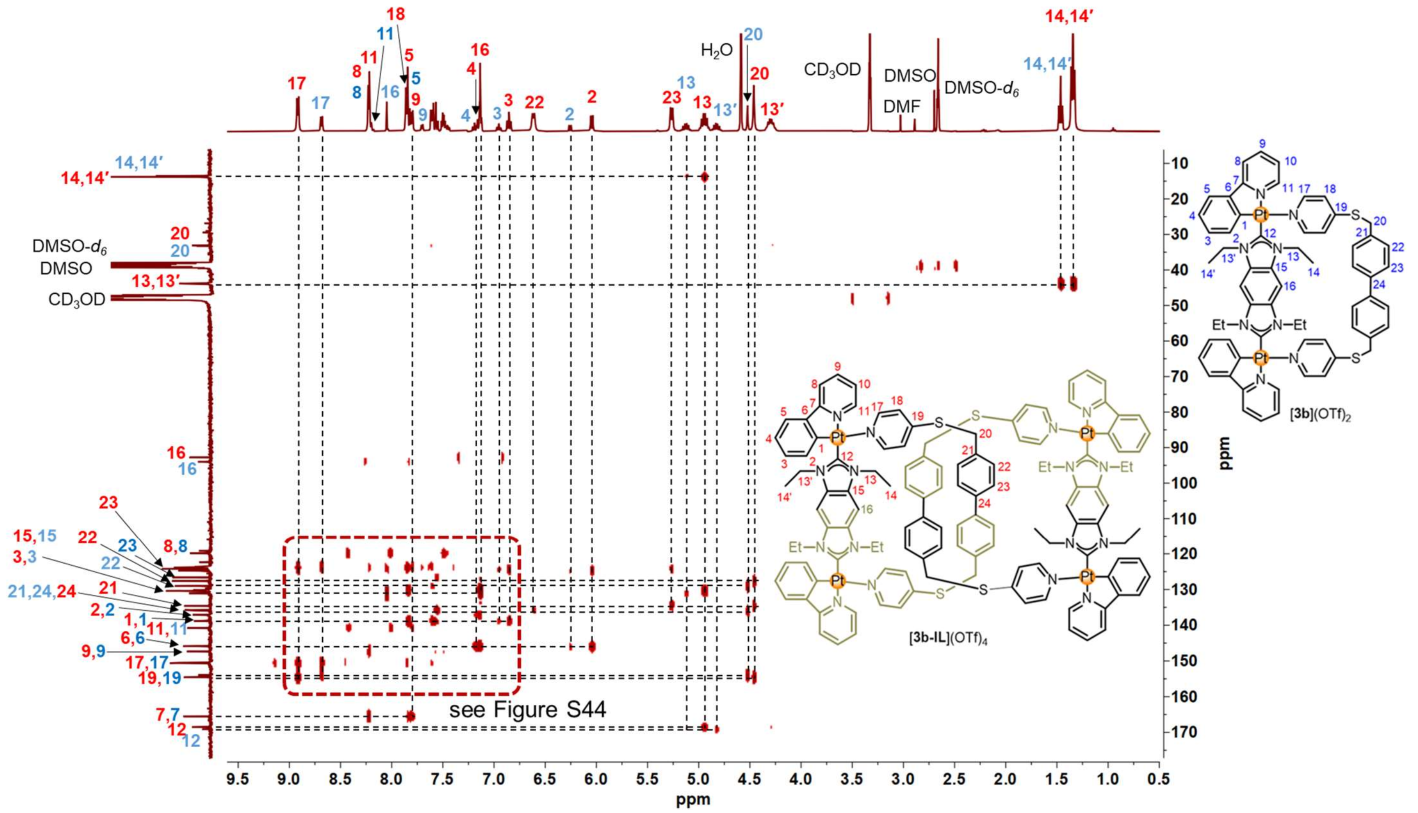

Figure S43. ^{1}H-^{13}C HMBC spectrum of the mixture [**3b**](OTf)$_2$ (blue) and [**3b-IL**](OTf)$_4$ (red) (CD$_3$OD/DMSO-*d*$_6$ v:v = 4:1, [4.0 m*M*].

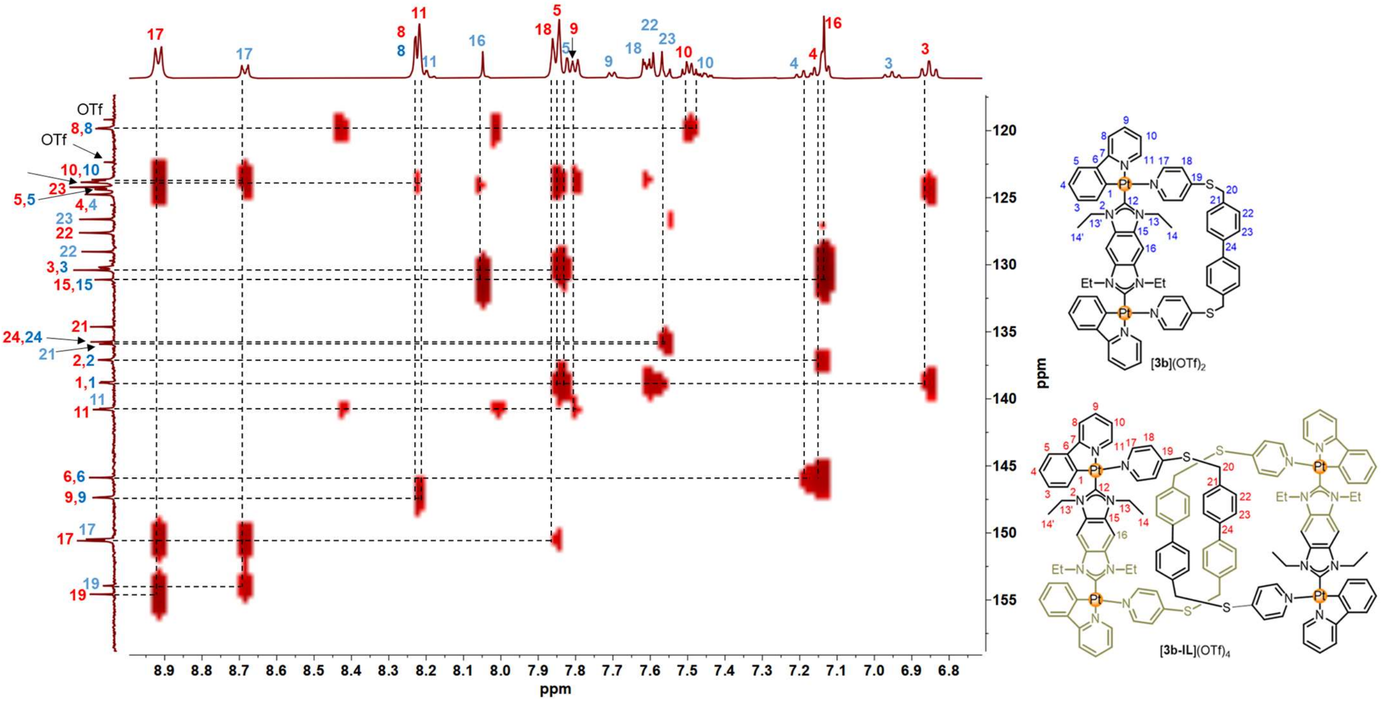

Figure S44. Partial ^{1}H-^{13}C HMBC spectrum of the mixture [**3b**](OTf)$_2$ (blue) and [**3b-IL**](OTf)$_4$ (red) (CD$_3$OD/DMSO-*d*$_6$ v:v = 4:1, [4.0 m*M*].

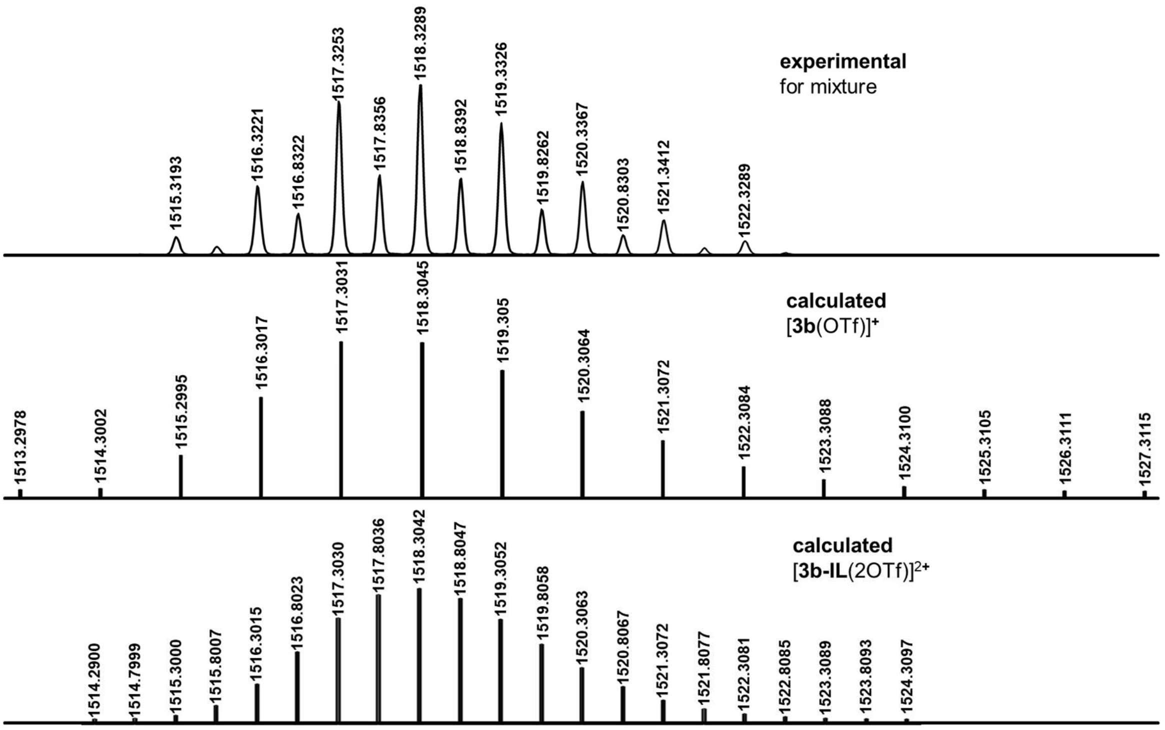

Figure S45. Section of the experimental (top) and calculated (2 x bottom) HRMS (ESI positive ions) spectra (positive ions) of the mixture [**3b**]$(OTf)_2$ and [**3b-IL**]$(OTf)_4$. Selected peaks can be assigned to both [**3b**(OTf)]$^+$ and [**3b-IL**$(2OTf)_2$]$^{2+}$ at essentially identical *m/z* values.

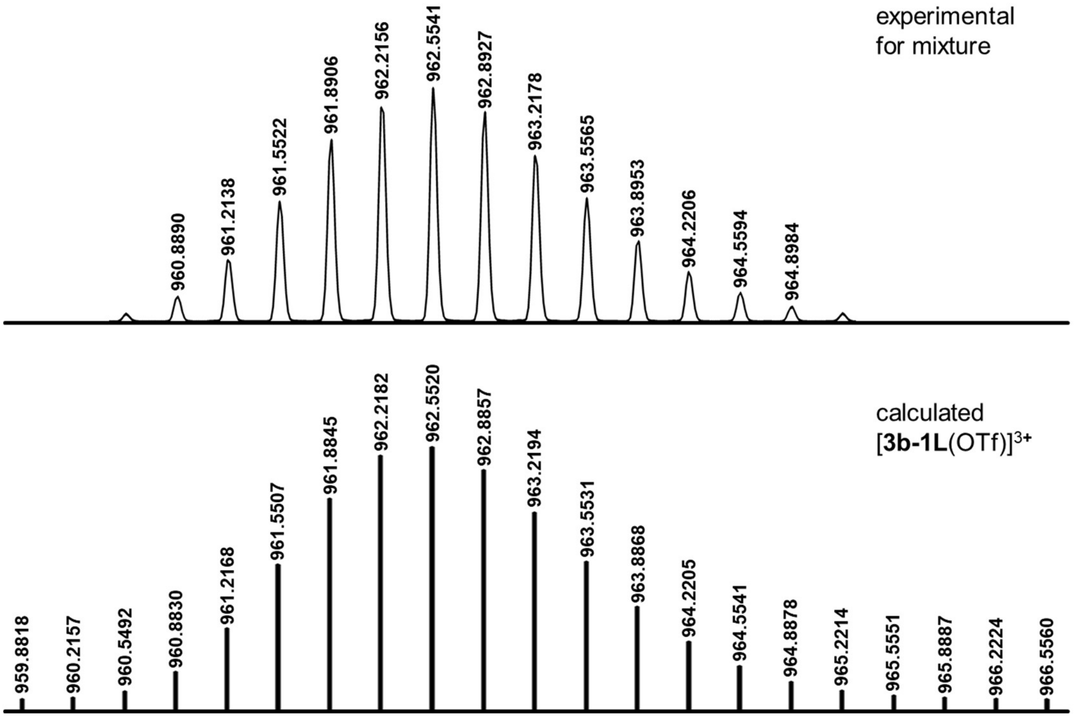

Figure S46. Section of the experimental (top) and calculated (bottom) HRMS (ESI positive ions) spectra (positive ions) of the mixture [**3b**]$(OTf)_2$ and [**3b-IL**]$(OTf)_4$. The peak at *m/z* = 962.5541 clearly shown the presence of [**3b-IL**(OTf)]$^{3+}$.

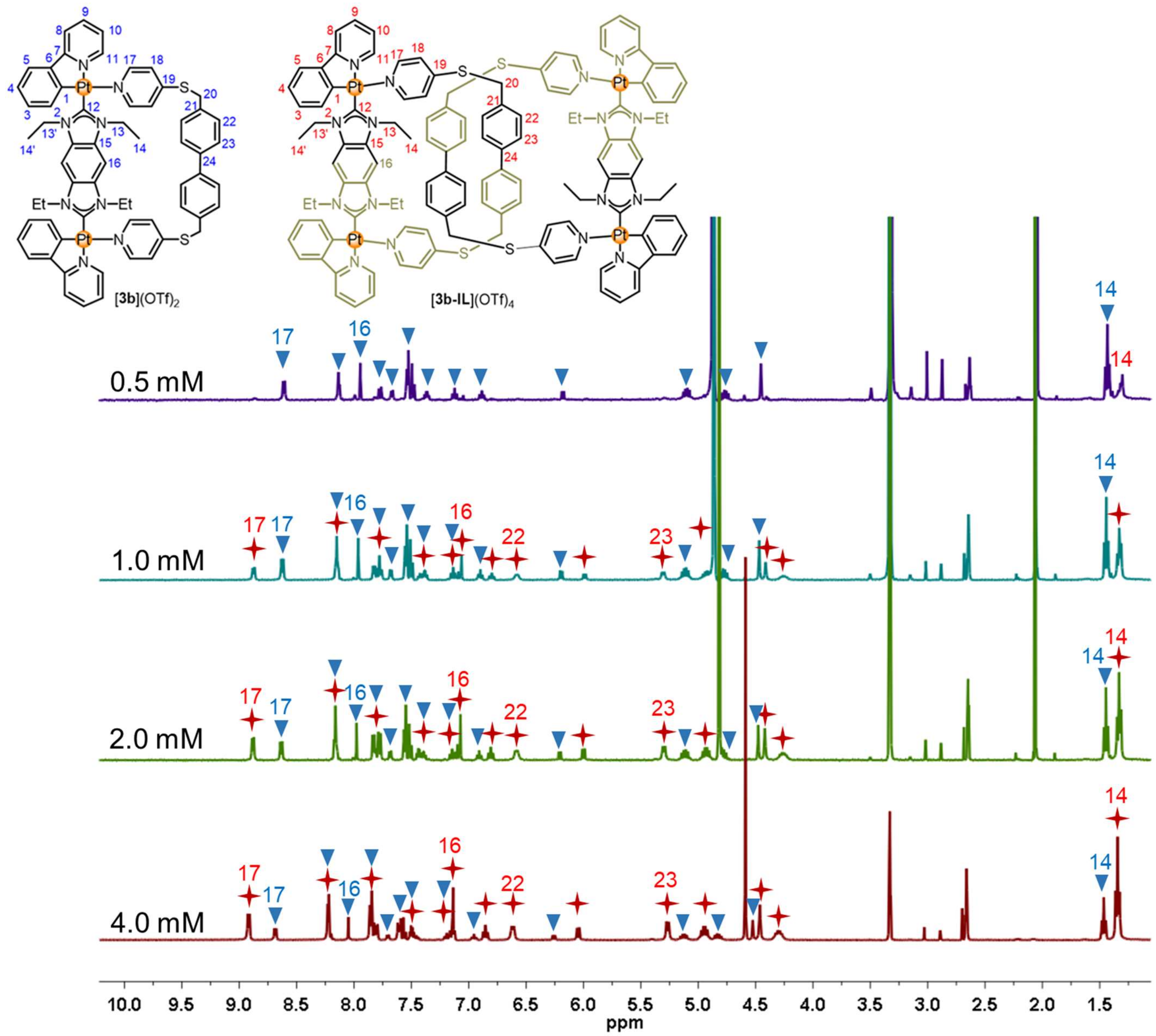

Figure S47. ^{1}H NMR spectra of mixtures of [**3b**](OTf)$_2$ (▼) + [**3b-IL**](OTf)$_4$(✦) (CD_3OD/DMSO-d_6 v:v = 4:1, 400 MHz, [4.0, 2.0, 1.0, 0.5 m*M*]) showing an increasing amount of [**3b-IL**](OTf)$_4$ in the mixture with increasing concentration.

3. Photophysical performance

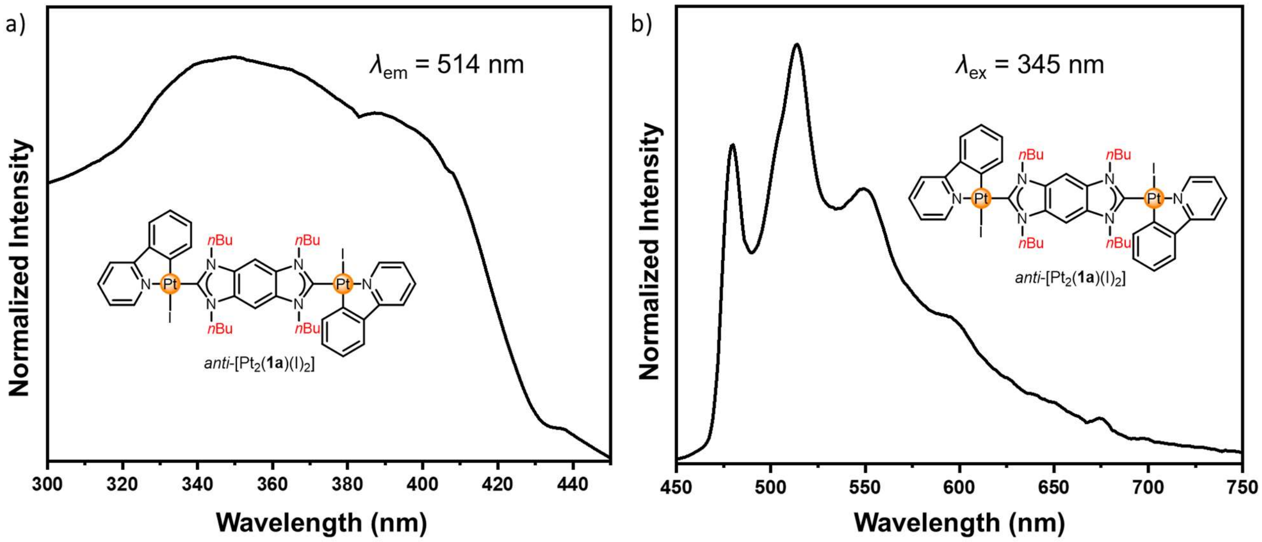

Figure S48. Excitation and emission spectra of *anti*-[Pt_2(**1a**)(I)$_2$] (solid state), λ_{ex} = 345 nm, λ_{em} = 514 nm.

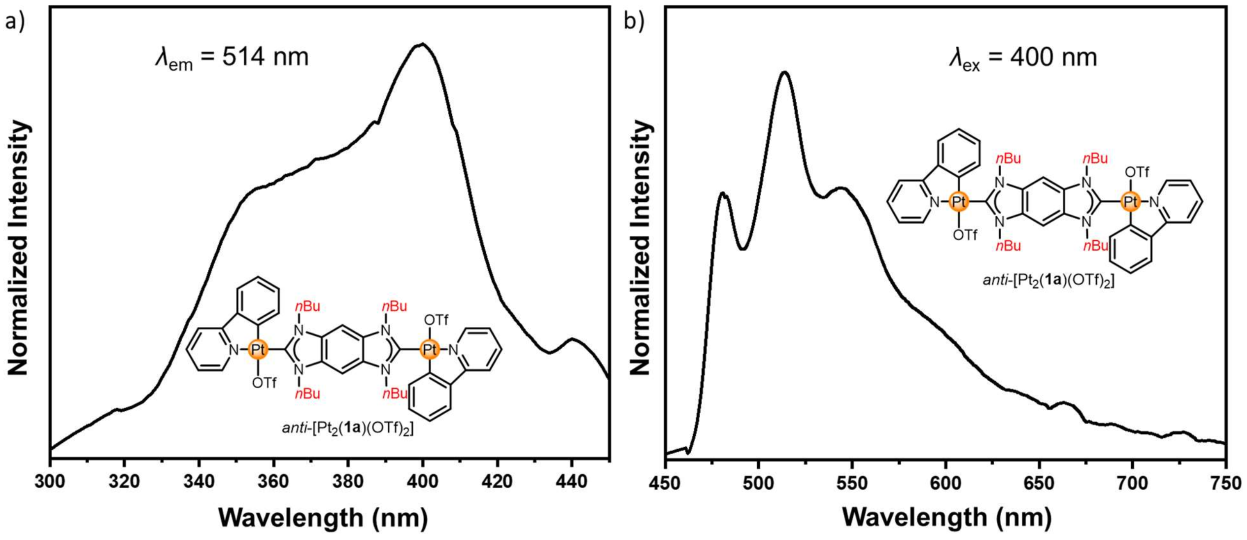

Figure S49. Excitation and emission spectra of *anti*-[Pt_2(**1a**)(OTf)$_2$] (solid state), λ_{ex} = 400 nm, λ_{em} = 514 nm.

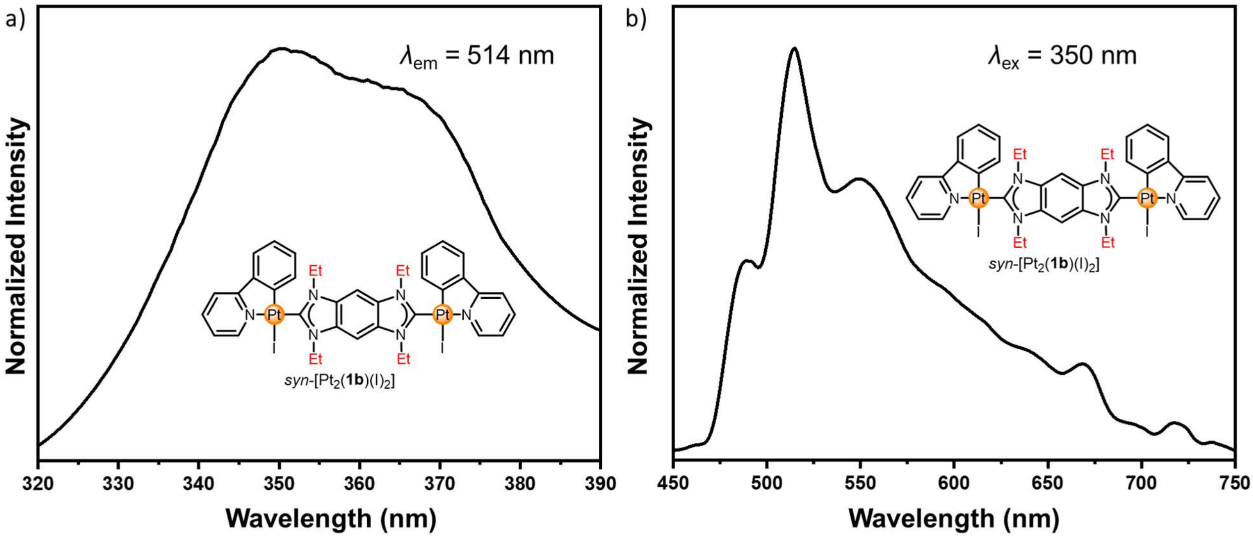

Figure S50. Excitation and emission spectra of *syn*-[Pt_2(**1b**)($I)_2$] (solid state), λ_{ex} = 350 nm, λ_{em} = 514 nm.

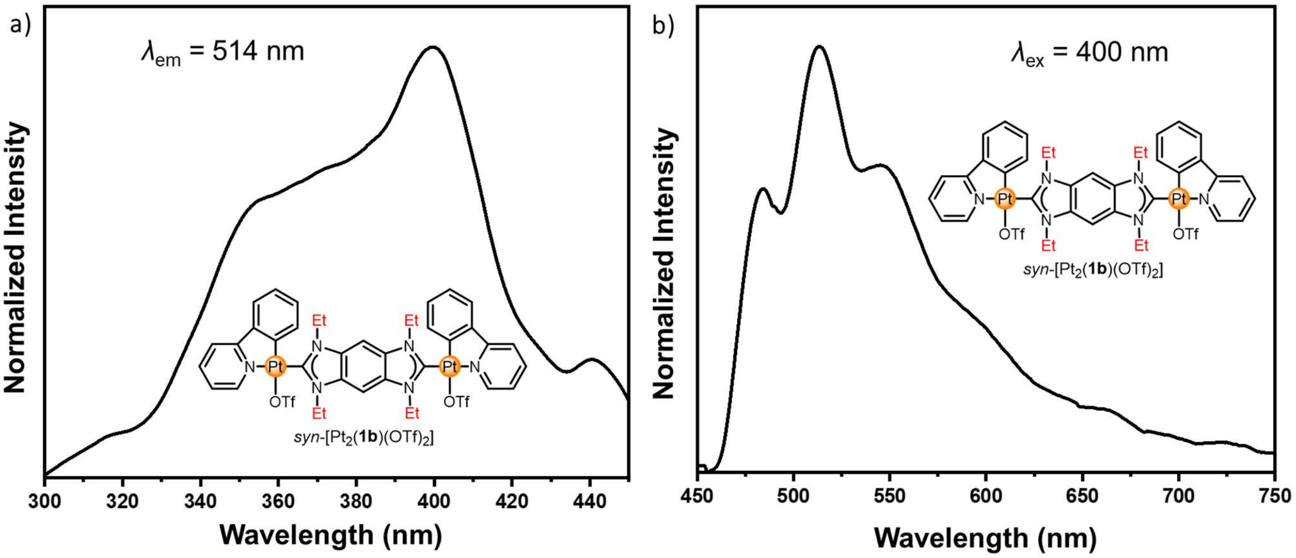

Figure S51. The excitation and emission spectra of *syn*-[Pt_2(**1b**)($OTf)_2$] (solid state), λ_{ex} = 400 nm, λ_{em} = 514 nm.

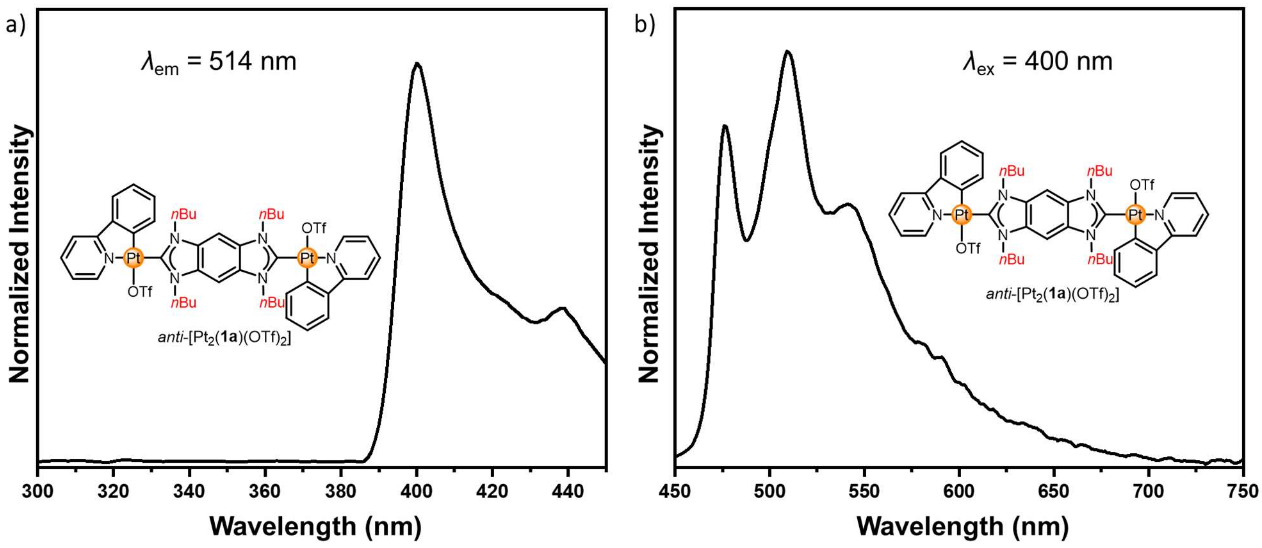

Figure S52. Excitation and emission spectra of *anti*-[Pt_2(**1a**)$(OTf)_2$] (CH_3OH, [3 m*M*]), λ_{ex} = 400 nm, λ_{em} = 514 nm.

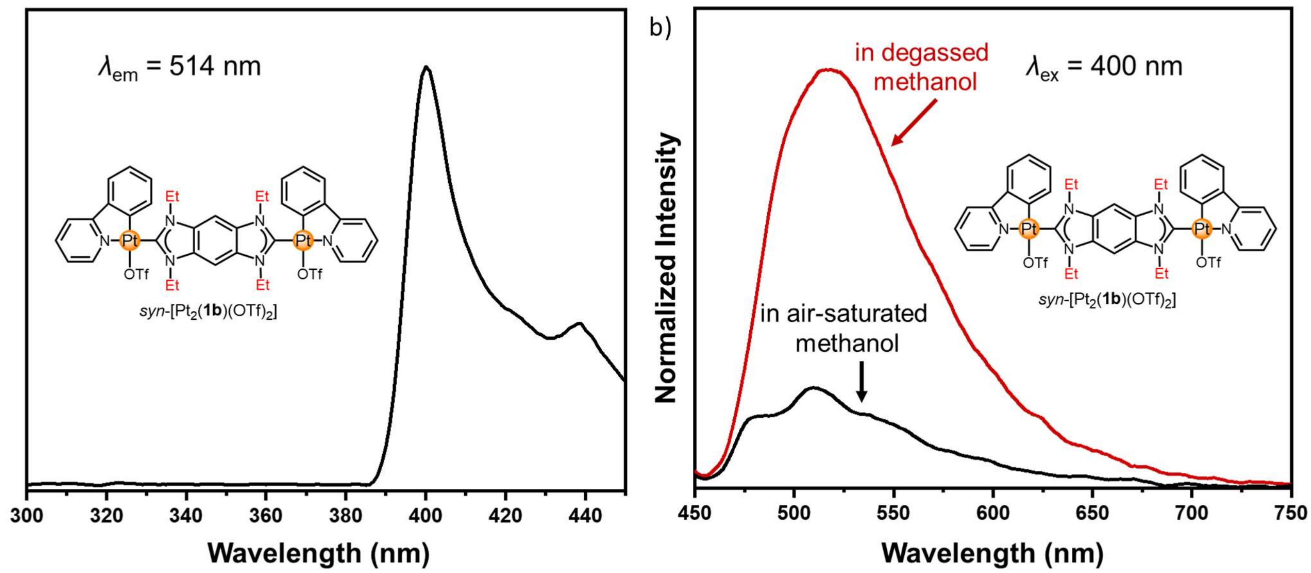

Figure S53. Excitation and emission spectra of *syn*-[Pt_2(**1b**)$(OTf)_2$] (CH_3OH, [3 m*M*]), λ_{ex} = 400 nm, λ_{em} = 514 nm: black line air saturated solution, red line degassed solution).

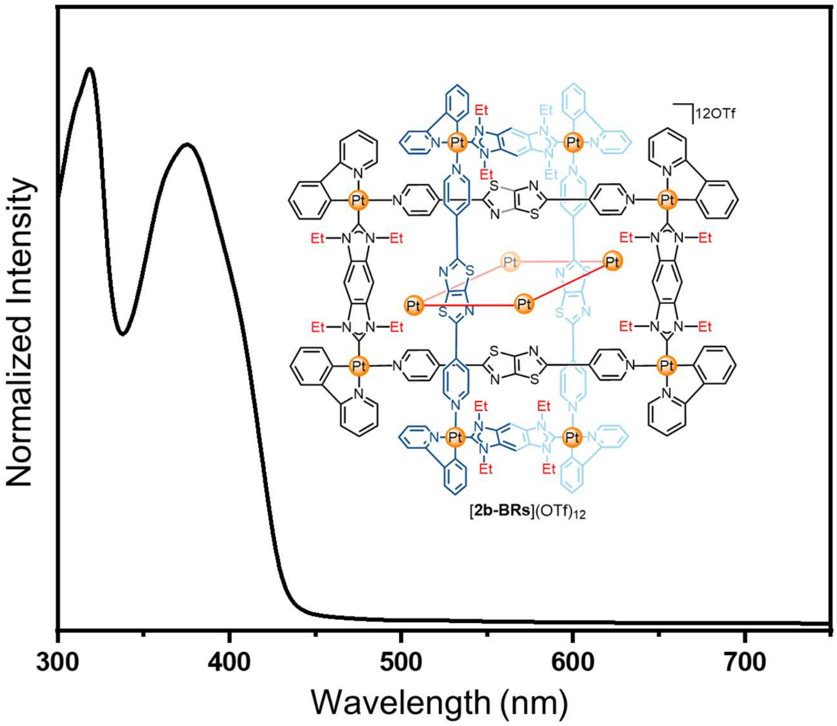

Figure S54. Ultraviolet-visible absorption spectrum of [**2b-BRs**]$(OTf)_{12}$ (CH_3OH/DMSO v:v = 4:1, [0.3 m*M*]).

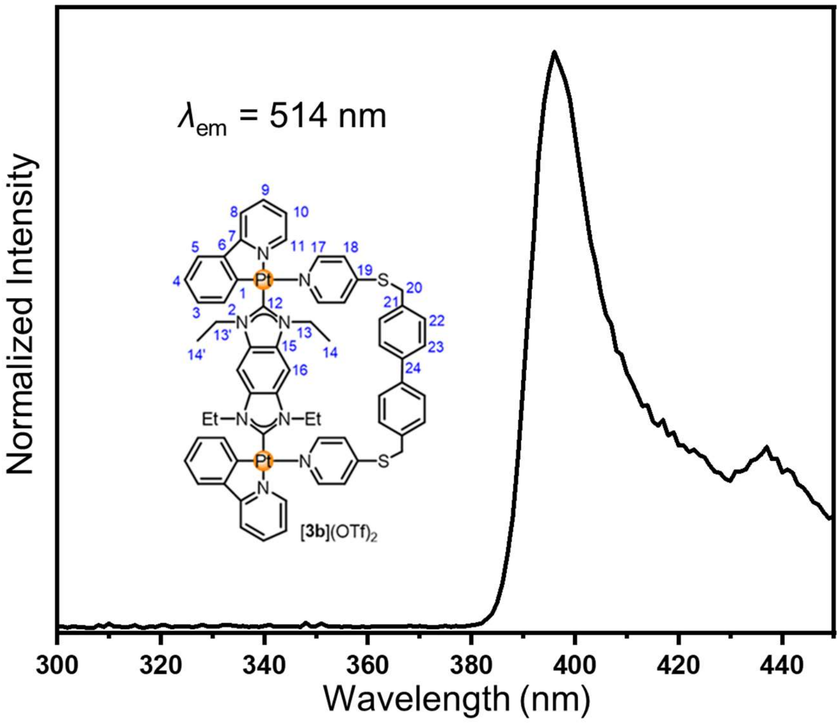

Figure S55. Excitation spectrum of [**3b**]$(OTf)_2$ (CH_3OH/DMSO v:v = 4:1, [0.5 m*M*]), λ_{em} = 514 nm.

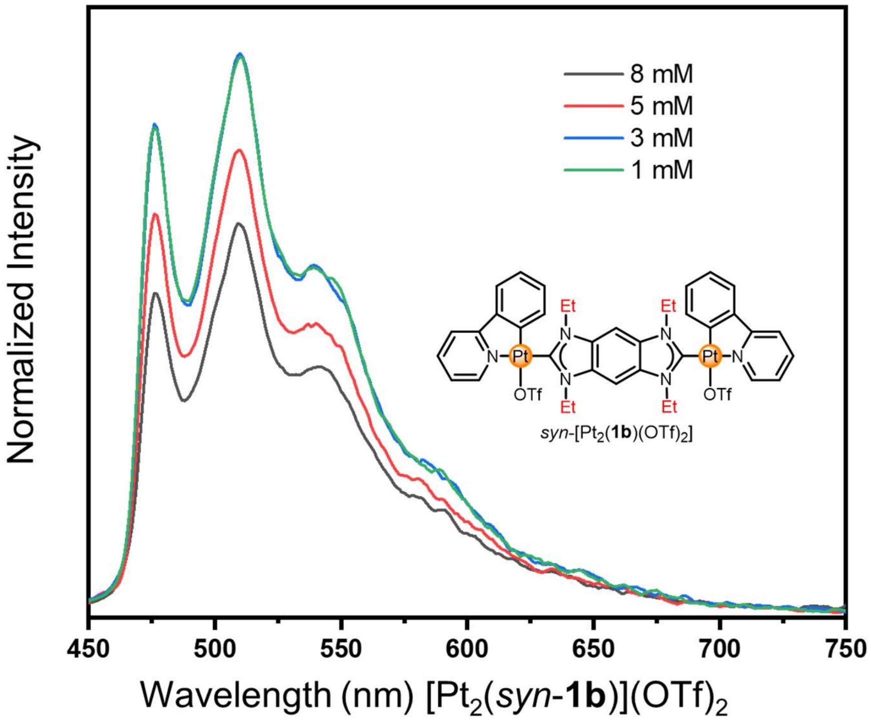

Figure S56. Emission spectra of *syn*-[Pt_2(**1b**)$(OTf)_2$] (CH_3OH, [8.0 m*M*, 5.0 m*M*, 3.0 m*M*, 1.0 m*M*], λ_{ex} = 400 nm) showing diminishing emission at increasing concentrations.

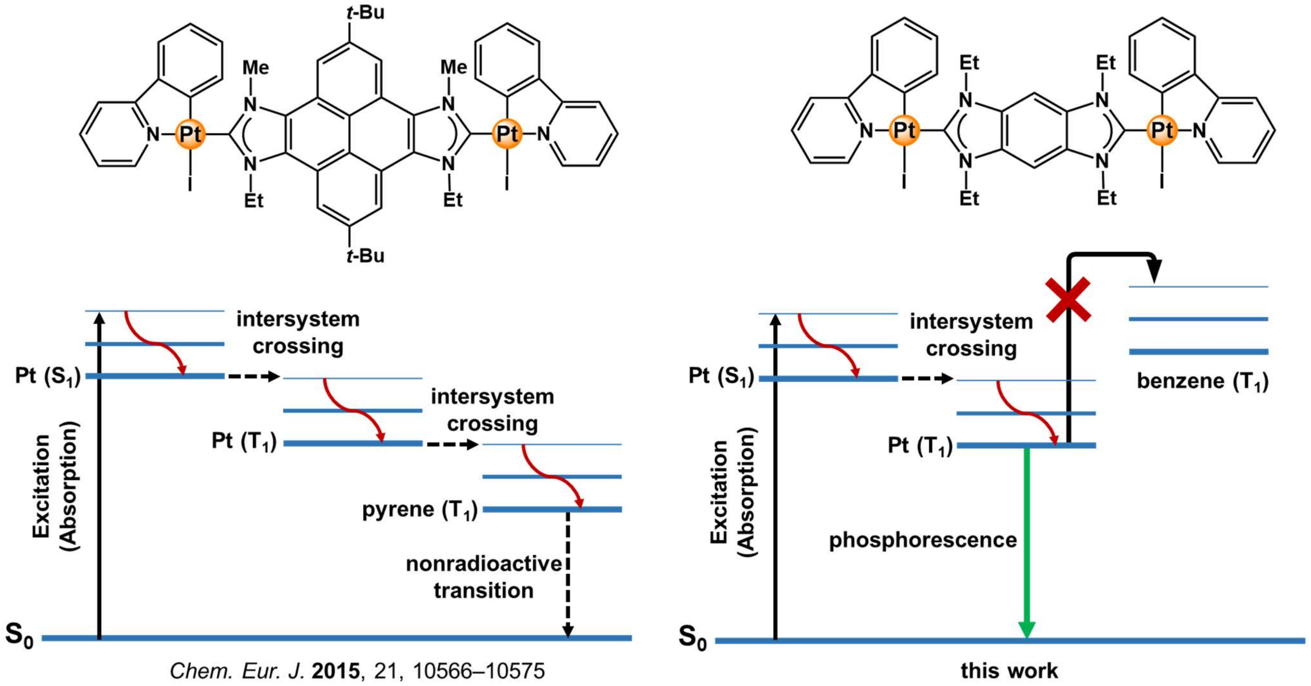

Figure S57. Schematic Jablonski diagrams illustrating the proposed photochemical pathways.[4]

4. X-ray diffraction studies

Yellow crystals of *anti*-[Pt_2(**1a**)(I)$_2$] and *syn*-[Pt_2(**1b**)(I)$_2$]·*n*-C_6H_{14}·2.5CH_2Cl_2·1.75H_2O were obtained by the slow diffusion of diethyl ether into a dichloromethane/DMSO solution (v:v = 4:1) of the compounds at ambient temperature. A thin hexane buffer layer (~0.5 mL) was placed between the complex solution (dichloromethane/DMSO) and the diethyl ether layer to slow down the diffusion of the diethyl ether into the solution of the complexes.

Yellow crystals of [**2b-BRs**](OTf)$_{12}$·40MeOH·25H_2O and [**3b-IL**](OTf)$_4$·10MeOH·6H_2O were obtained by slow diffusion of isopropyl ether into a methanol/DMSO/DMF solution (ca. v:v:v = 5:1:1) of the compounds at ambient temperature.

Diffraction data for *anti*-[Pt_2(**1a**)(I)$_2$], **BRs**](OTf)$_{12}$·40MeOH·25H_2O and [**3b-IL**](OTf)$_4$·10MeOH·6H_2O were collected at 193K, 193K and 157K, respectively, using a Bruker-CCD SMART APEX diffractometer and Ga-K$_\alpha$ (λ = 1.34138 Å) radiation. Diffraction data for *syn*-[Pt_2(**1b**)(I)$_2$]·*n*-hexane·2.5CH_2Cl_2·1.75H_2O were collected at 203K using a Bruker Bruker-CCD SMART APEX diffractometer using Mo-Kα radiation (λ = 0.71073 Å).

Indexing was performed using APEX 2 (difference vectors method). Data integration and reduction were performed using SaintPlus 6.01. Absorption correction was performed by the multiscan method implemented in SADABS. Structure solutions were found with SHELXT[S5] and were refined with SHELXL-97[S6] using first isotropic and later anisotropic thermal parameters for all non-hydrogen atoms.

The asymmetric unit of *anti*-[Pt_2(**1a**)(I)$_2$] contains ½ formula unit. The complex resides on a crystallographic inversion center located at the midpoint of the phenyl group of the benzobiscarbene ligand.

The asymmetric unit of *syn*-[Pt_2(**1b**)(I)$_2$](I)$_2$·*n*-C_6H_{14}·2.5 CH_2Cl_2·1.75H_2O contains four formula units (space group $Pna2_1$). The solvent molecules in the asymmetric unit (total of 4 *n*-hexane, 10 dichloromethane and 7 water molecules) could not be restrained properly. Therefore, the SQUEEZE algorithm was used to omit them. A total of 59 ISOR, 6 DELU, 2 DANG and 30 DFIX instructions were used to restrain the ligands leading to a total of 394 restraints in the refinement.

The asymmetric unit of [**2b-BRs**](OTf)$_{12}$·40MeOH·25H_2O contains two formula units (space group $P2_1/c$). The asymmetric unit contains disordered anions and solvent molecules

(22 $CF_3SO_3^-$ anions, 8 CH_3OH and 50 H_2O molecules) which could not be restrained properly. Therefore, the SQUEEZE algorithm was used to omit them. Several N-ethyl groups of the benzobiscarbene ligands are disordered. They were divided into two parts (57:43, 90:10, 76:24, 72:28, 77:23, 71:29 and 60:40). A total of 166 ISOR, 3 DANG, 2 DELU, 18 SIMU and 173 DFIX instructions were used to restrain anions and ligands leading to a total of 1191 restraints in the refinement. Due to the exceptionally large unit cell volume (100909 Å^3) and the complexity of the supramolecular structure, the diffraction intensity at high angles was inherently weak. The current resolution (0.95 Å) is sufficient to unambiguously determine the overall molecular structure and connectivity. The Borromean structure is further fully corroborated by ESI-MS and NMR spectroscopy.

The asymmetric unit of [**3b-IL**]$(OTf)_4$·10MeOH·6H_2O contains one formula unit (space group $P2_1/n$). The asymmetric unit contains disordered anion and solvent molecules (1 $CF_3SO_3^-$ anion, 10 CH_3OH and 6 H_2O molecules) which could not be restrained properly. Therefore, SQUEEZE algorithm was used to omit them. Two ethyl group were disordered and they were divided into two parts (50:50). 15 ISOR, 1 SIMU, 1 DELU and 6 DFIX instructions were used to restrain anions and ligands leading to a total of 103 restraints in the refinement.

Crystallographic data have been deposited in the Cambridge Crystallographic Data Centre under accession numbers CCDC 2530878 (for *anti*-$[Pt_2($**1a**$)(I)_2]$) CCDC 2530877 (for *syn*-$[Pt_2($**1b**$)(I)_2]$·*n*-C_6H_{14}·2.5CH_2Cl_2·1.75H_2O), CCDC 2530879 (for [**2b-BRs**]$(OTf)_{12}$·40MeOH·25H_2O) and CCDC 2530880 (for [**3b-IL**]$(OTf)_4$·10MeOH·6H_2O).

Table S1. Crystal data and structure refinement for *anti*-[Pt_2(**1a**)(I)$_2$]

Empirical formula	$C_{46}H_{54}N_6I_2Pt_2$
Formula weight	1334.93
Temperature/K	193
Crystal system	orthorhombic
Space group	*Pbca*
a/Å	9.8772(5)
b/Å	16.5044(9)
c/Å	28.6324(14)
α/°	90
β/°	90
γ/°	90
Volume/Å^3	4667.6(4)
Z	4
ρ_{calc}/g·/cm^3	1.900
μ/mm^{-1}	14.932
F(000)	2536
Crystal size/mm^3	0.22 × 0.17 × 0.17
Radiation	GaKα (λ = 1.34139)
2Θ range for data collection/°	9.47 to 120.0
Index ranges	$-12 \le h \le 12$, $-19 \le k \le 21$, $-36 \le l \le 36$
Reflections collected	25800
Independent reflections	5227 [R_{int} = 0.0656]
Data/restraints/parameters	5227/50/293
Goodness-of-fit on F2	1.087
Final R indexes [I>=2σ(I)]	R_1 = 0.0583, wR_2 = 0.1470
Final R indexes [all data]	R_1 = 0.0732, wR_2 = 0.1605
Largest diff. peak/hole / e·Å^{-3}	2.69/–1.67

$R_1 = \Sigma||F_0|-|F_c||$ (based on reflections with $F_0^2>2\sigma F^2$). $wR_2 = [\Sigma[w(F_0-F_c^2)^2]/\Sigma[w(F_0)^2]]^{1/2}$, $w=1/[\sigma^2(F_0^2)+(0.095P)^2]$, $P = [\max(F_0^2,0)+2F_c^2]/3$ (also with $F_0^2>2\sigma F^2$)

Table S2. Crystal data and structure refinement for *syn*-[Pt_2(**1b**)$(I)_2$]·*n*-C_6H_{14}·2.5CH_2Cl_2·1.75H_2O

Empirical formula	$C_{46.5}H_{60.5}N_6Cl_5I_2O_{1.75}Pt_2$
Formula weight	1552.74
Temperature/K	203
Crystal system	orthorhombic
Space group	$Pna2_1$
a/Å	30.439(3)
b/Å	13.0900(13)
c/Å	49.613(4)
α/°	90
β/°	90
γ/°	90
Volume/Å^3	19768(3)
Z	16
ρ_{calc}/g·cm^{-3}	2.087
μ/mm^{-1}	7.220
F(000)	11880
Crystal size/mm^3	0.25 × 0.1 × 0.08
Radiation	MoKα (λ = 0.71073)
2Θ range for data collection/°	3.64 to 50.50
Index ranges	$-36 \le h \le 36$, $-15 \le k \le 15$, $-57 \le l \le 59$
Reflections collected	123224
Independent reflections	35712 [R_{int} = 0.1606]
Data/restraints/parameters	35712/394/1746
Goodness-of-fit on F^2	0.967
Final *R* indexes [I>=2σ(I)]	R_1 = 0.0572, wR_2 = 0.1167
Final *R* indexes [all data]	R_1 = 0.1103, wR_2 = 0.1388
Largest diff. peak/hole / e·$^-$Å^{-3}	0.98/–1.07
Flack parameter	0.316(8)

$R_1 = \Sigma||F_0|-|F_c||$ (based on reflections with $F_0^2>2\sigma F^2$). $wR_2 = [\Sigma[w(F_0-F_c^2)^2]/\Sigma[w(F_0)^2]]^{1/2}$, $w=1/[\sigma^2(F_0^2)+(0.095P)^2]$, $P = [\max(F_0^2,0)+2F_c^2]/3$ (also with $F_0^2>2\sigma F^2$)

Table S3. Crystal data and structure refinement for [**2b-BRs**]$(OTf)_{12}\cdot 40MeOH\cdot 25H_2O$

Empirical formula	$C_{364}H_{486}N_{60}F_{36}O_{101}Pt_{12}S_{24}$
Formula weight	11112.62
Temperature/K	193
Crystal system	monoclinic
Space group	$P2_1/c$
a/Å	39.792(6)
b/Å	68.980(9)
c/Å	36.773(5)
α/°	90
β/°	91.350(5)
γ/°	90
Volume/Å^3	100909(25)
Z	8
ρ_{calc}/g·cm^{-3}	1.463
μ/mm^{-1}	5.382
F(000)	44336
Crystal size/mm^3	0.35 × 0.25 × 0.2
Radiation	GaKα (λ = 1.34139)
2Θ range for data collection/°	4.86 to 90.22
Index ranges	$-41 \le h \le 41$, $-72 \le k \le 72$, $-38 \le l \le 38$
Reflections collected	453235
Independent reflections	123825 [R_{int} = 0.0569]
Data/restraints/parameters	123825/1394/7473
Goodness-of-fit on F^2	1.047
Final R indexes [I>=2σ (I)]	R_1 = 0.0741, wR_2 = 0.2284
Final R indexes [all data]	R_1 = 0.1114, wR_2 = 0.2616
Largest diff. peak/hole / e Å^{-3}	4.01/–1.30

$R_1 = \Sigma||F_0|-|F_c||$ (based on reflections with $F_0^2>2\sigma F^2$). $wR_2 = [\Sigma[w(F_0-F_c^2)^2]/\Sigma[w(F_0)^2]]^{1/2}$, $w=1/[\sigma^2(F_0^2)+(0.095P)^2]$, $P = [\max(F_0^2,0)+2F_c^2]/3$ (also with $F_0^2>2\sigma F^2$)

Table S4. Crystal data and structure refinement for [**3b-IL**]$(OTf)_4 \cdot 10MeOH \cdot 6H_2O$)

Empirical formula	$C_{138}H_{168}N_{16}F_{12}O_{28}Pt_4S_8$
Formula weight	3763.71
Temperature/K	157
Crystal system	monoclinic
Space group	$P2_1/n$
a/Å	19.217(2)
b/Å	42.957(5)
c/Å	19.232(2)
α/°	90
β/°	98.382(4)
γ/°	90
Volume/Å^3	15707(3)
Z	4
ρ_{calc}/g·cm^{-3}	1.592
μ/mm^{-1}	5.606
F(000)	7520
Crystal size/mm^3	0.45 × 0.3 × 0.15
Radiation	GaKα (λ = 1.34139)
2Θ range for data collection/°	5.4 to 119
Index ranges	$-23 \leq h \leq 24$, $-55 \leq k \leq 55$, $-24 \leq l \leq 24$
Reflections collected	113486
Independent reflections	34710 [R_{int} = 0.0449]
Data/restraints/parameters	34710/103/1597
Goodness-of-fit on F^2	1.019
Final R indexes [I>=2σ(I)]	R_1 = 0.0704, wR_2 = 0.1766
Final R indexes [all data]	R_1 = 0.0862, wR_2 = 0.1873
Largest diff. peak/hole / e·Å^{-3}	2.23/ –2.20

$R_1 = \Sigma||F_0|-|F_c||$ (based on reflections with $F_0^2>2\sigma F^2$). $wR_2 = [\Sigma[w(F_0-F_c^2)^2]/\Sigma[w(F_0)^2]]^{1/2}$, $w=1/[\sigma^2(F_0^2)+(0.095P)^2]$, $P = [\max(F_0^2,0)+2F_c^2]/3$ (also with $F_0^2>2\sigma F^2$)

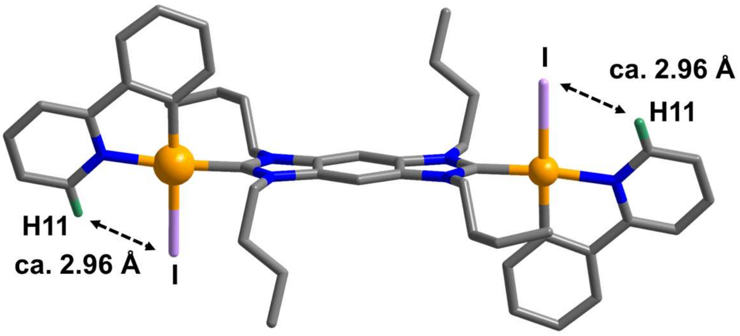

Figure S58. Molecular structure of *anti*-[Pt_2(**1a**)(I)$_2$]. The molecule resides on a crystallographic inversion center (color code: C, grey; N, blue; Pt, orange; I, violet, hydrogen atoms except for H11 are omitted).

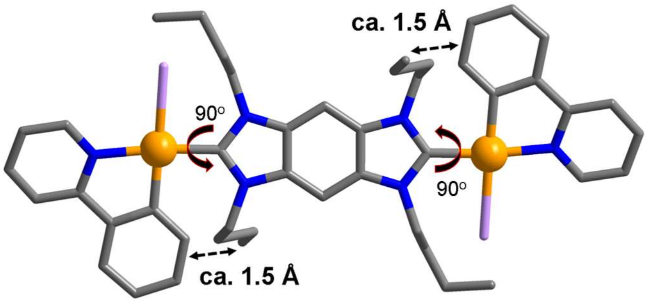

Figure S59. Structural model of *anti*-[Pt_2(**1a**)(I)$_2$]. Rotation of the Pt(ppy) moieties by 90° (to a coplanar arrangement with the benzobiscarbene core) results in a prohibitively short distance (~1.5 Å) between one of the ppy carbon atom and one of the alkyl carbon atoms.

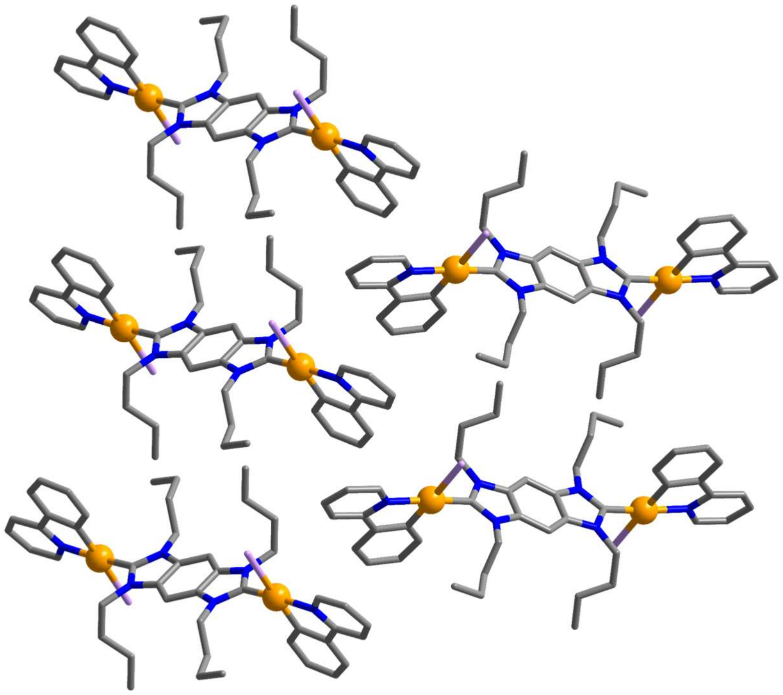

Figure S60. Packing of the *anti*-[Pt_2(**1a**)(I)$_2$] complexes in the crystal lattice showing no interactions between the complexes.

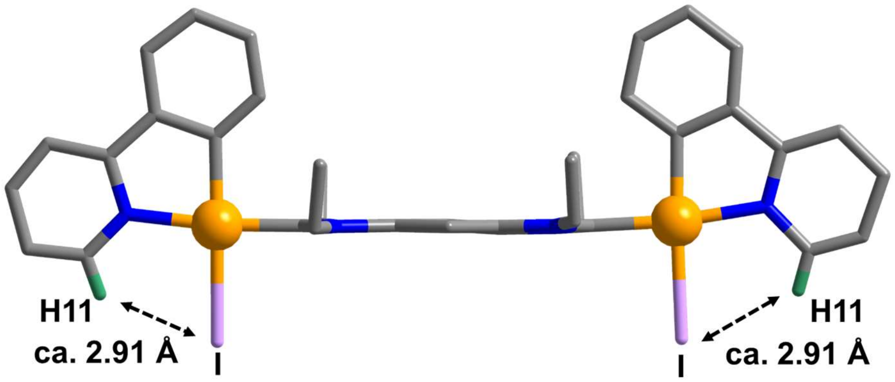

Figure S61. Molecular structure of *syn*-[Pt_2(**1b**)$(I)_2$] in *syn*-[Pt_2(**1b**)$(I)_2$]·*n*-C_6H_{14}·2.5CH_2Cl_2·1.75H_2O (color code: C, grey; N, blue; Pt, orange; I, violet, hydrogen atoms except for H11 are omitted).

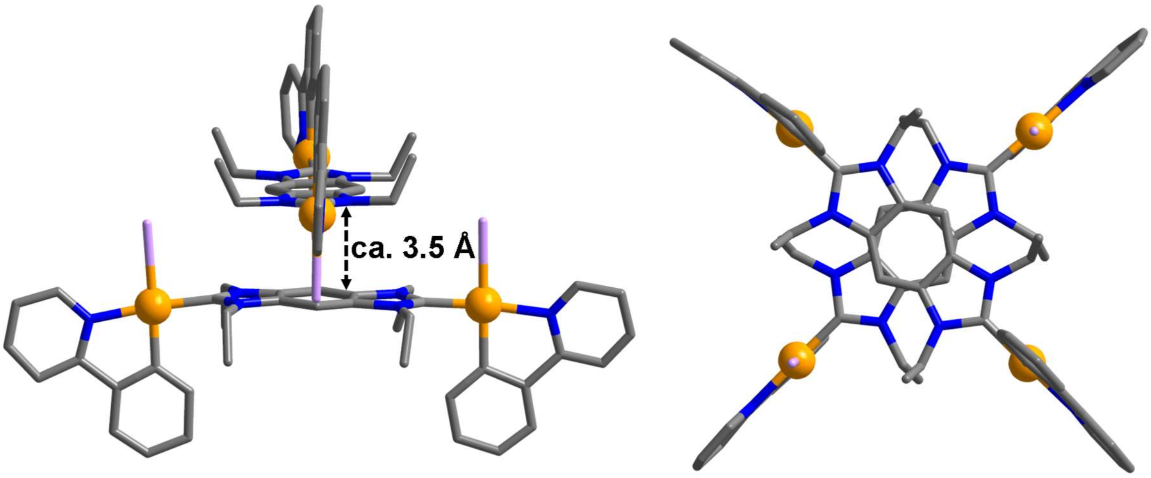

Figure S62. Packing of the *syn*-[Pt_2(**1b**)$(I)_2$] complexes in the crystal lattice showing the essentially coplanar arrangement of the aromatic portions of the bridging ligands.

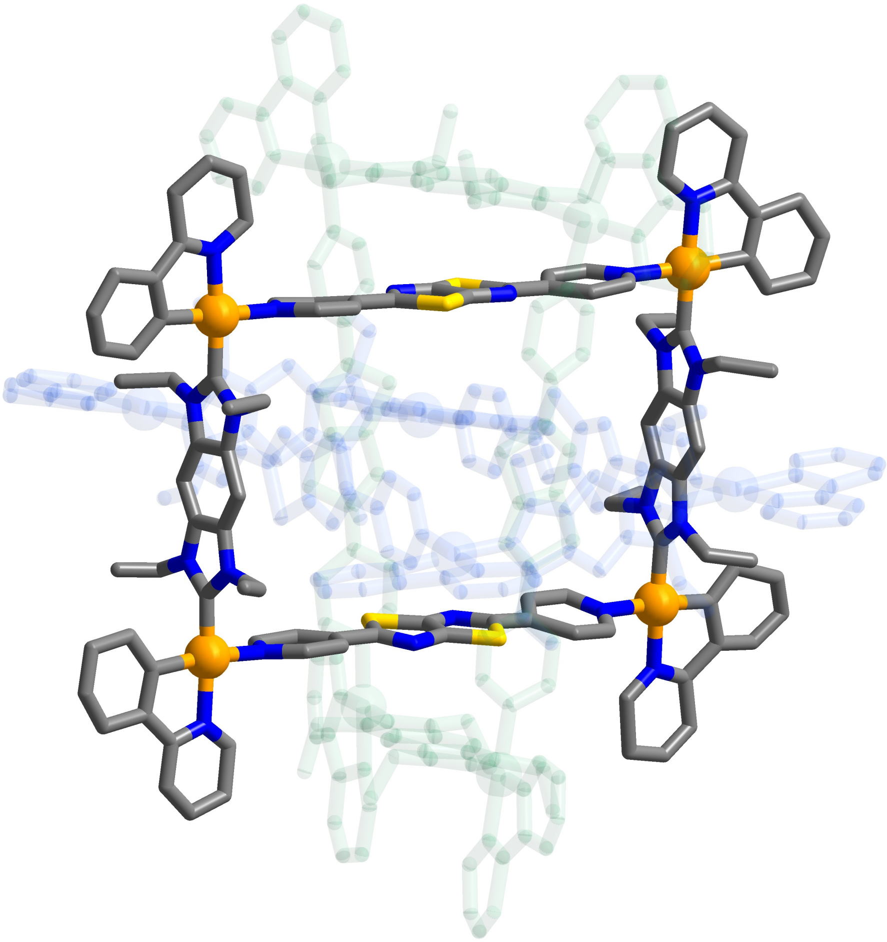

Figure S63. Molecular structure of [**2b-BR**]$(OTf)_{12}$ (color code: C, grey; N, blue; Pt, orange; S, yellow, hydrogen atoms and 12 triflate anions are omitted).

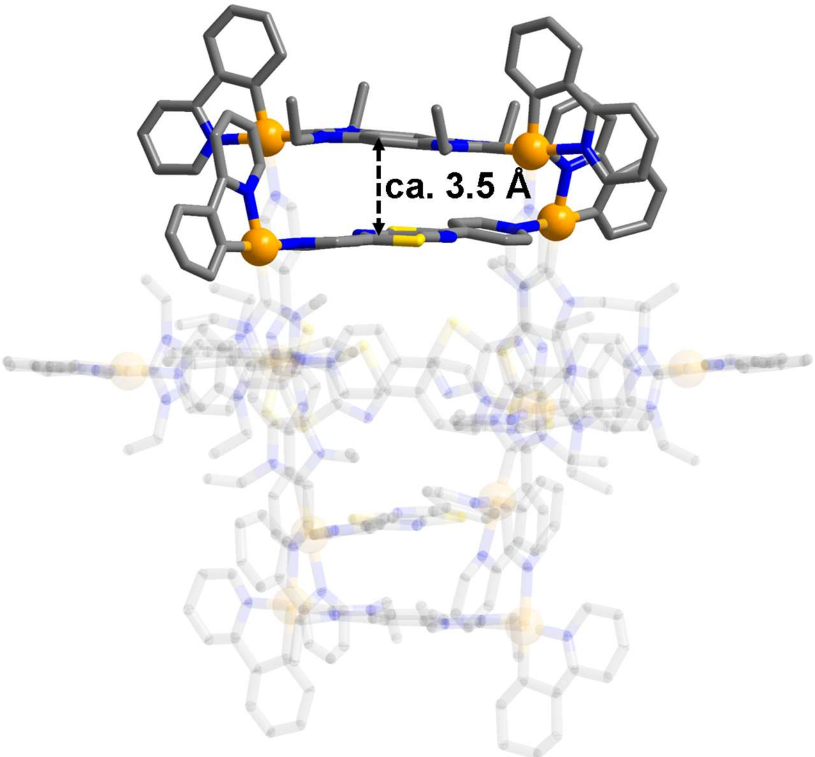

Figure S64. Molecular structure of [**2b-BR**]$(OTf)_{12}$. The distance between benzobiscarbene ligands and thiazolothiazole moiety measures approximately 3.5 Å.

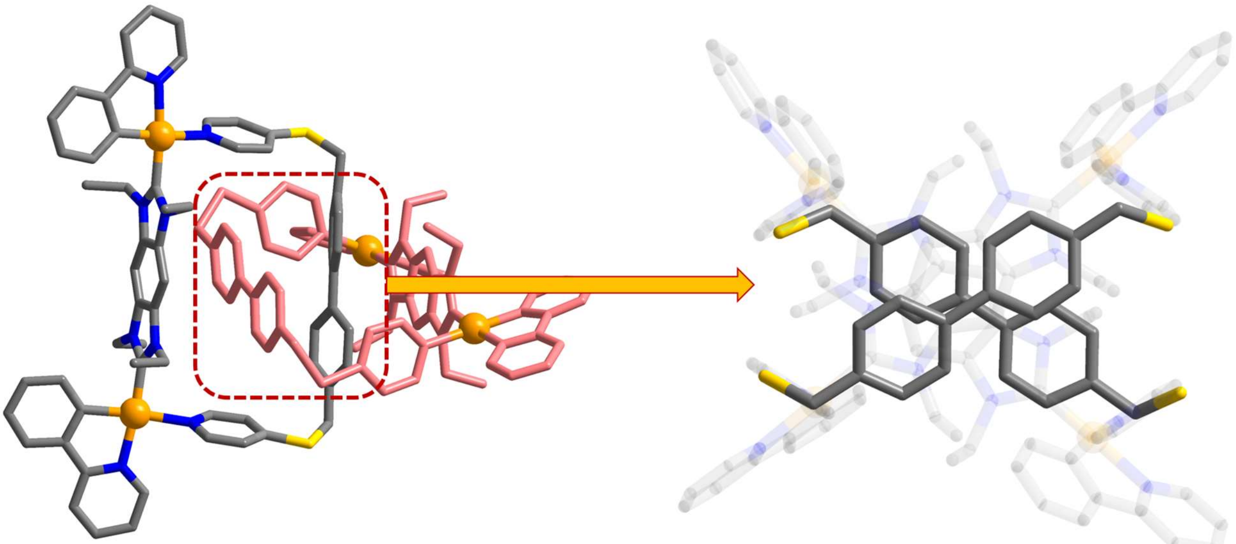

Figure S65. Left: molecular structure of [**3b-IL**]$(OTf)_4$, (N, blue; C, gray; Pt, orange; S, yellow; hydrogen atoms and counter anions have been omitted for clarity). Right: two biphenyl groups from different bipyridyl ligands are arranged almost perpendicular and no $\pi\cdots\pi$ interaction is possible.

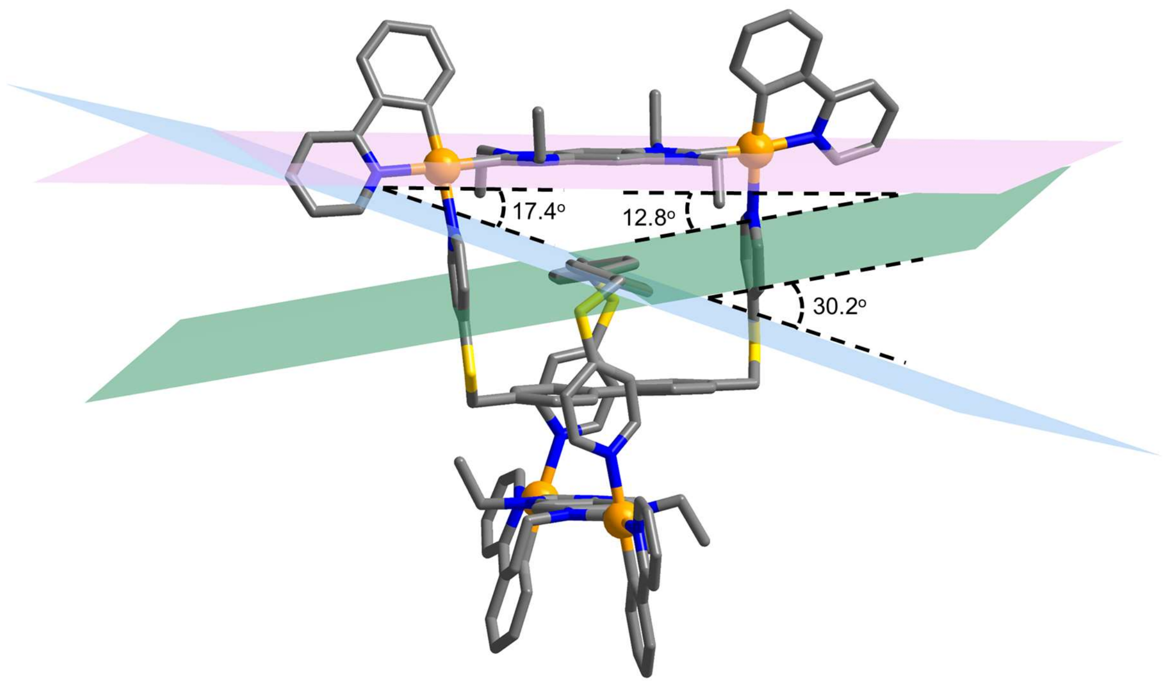

Figure S66. Sections of the molecular structure of [**3b-IL**](OTf)$_4$, (N, blue; C, gray; Pt, orange; S, yellow; hydrogen atoms and counter anions have been omitted for clarity). The two phenyl rings of the diphenyl ligand are rotated relative to each other by 30.2°. The plane of bis-NHC ligand is not oriented parallel with any planes of the biphenyl groups (dihedral angles 17.4° and 12.8°).

5. Reference

[S1] H. Geng, K.-J. Luo, G. Zou, H.-F. Wang, H.-L Ni, W.-H. Yu, Q. Lia, Y.-H. Wang, *New J. Chem.* **2016**, *40*, 10371–10377.

[S2] Y. Lu, P. D. Dutschke, J. Kinas, A. Hepp, G.-X. Jin, F. E. Hahn, *Angew. Chem. Int. Ed.* **2023**, *62,* e202217681; *Angew. Chem.* **2023***, 135,* e202217681.

[S3] H.-J. Feng, W.-X. Gao, Y.-J. Lin, G.-X. Jin, *Chem. Asian J.* **2019**, *14*, 2712–2718.

[S4] S. Ibáñez, A. Guerrero, M. Poyatos, E. Peris, *Chem. Eur. J.* **2015**, *21*, 10566–10575.

[S5] G. M. Sheldrick, *Acta. Cryst*. **2015**, *A71*, 3–8.

[S6] G. M. Sheldrick, *Acta. Cryst.* **2015**, *C71*, 3-8.
